# Divergent Synthesis of Möbius and Hückel N‐Heterocycloarenes From a Common Macrocyclic Backbone

**DOI:** 10.1002/anie.2790811

**Published:** 2026-07-07

**Authors:** Han Chen, Huishu Ma, Daiyue Yang, Zheng Zhou, Xiao Chen, Zhifeng Huang, Qian Miao

**Affiliations:** ^1^ Department of Chemistry The Chinese University of Hong Kong Shatin Hong Kong China; ^2^ Shanghai‐Hong Kong Joint Laboratory in Chemical Synthesis Shanghai Institute of Organic Chemistry Chinese Academy of Sciences Shanghai China; ^3^ School of Materials Science and Engineering Tongji University Shanghai China; ^4^ Shenzhen Research Institute The Chinese University of Hong Kong Shenzhen Guangdong China; ^5^ Shanghai‐Hong Kong Joint Laboratory in Chemical Synthesis The Chinese University of Hong Kong Shatin Hong Kong China

**Keywords:** cycloarenes, divergent synthesis, N‐hetero polycyclic arenes, Scholl reaction, strain

## Abstract

We report the divergent synthesis of a Möbius N‐heterocycloarene (**1**) and a Hückel N‐heterocycloarene (**2**) from a common macrocyclic precursor. The topology is governed by the annulation reaction: the Scholl reaction yields the Möbius topology, while InCl_3_‐mediated alkyne annulation gives the Hückel topology. In acyclic models, both reactions favor C4/C5 over C2/C7 bond formation, but this regioselectivity reverses in the constrained macrocycle. The Scholl reaction forms three bonds at C2, one at C7, and two at C5—one inducing the 180° twist for the Möbius topology and the other accompanied by a skeletal rearrangement, whereas alkyne annulation exclusively forms C2/C7 bonds to afford the Hückel topology. Crystal structure analysis and computational studies show uneven contortion in **1** and localized aromatic ring currents in both **1** and **2**. Compound **2** adopts a saddle‐shaped geometry with high conformational flexibility and binds tetrafluoroborate via C–H···F hydrogen bonds in solution.

## Graphical abstract

In the divergent synthesis of Möbius and Hückel N‐heterocycloarenes from a common macrocyclic precursor, the annulation reaction dictates topology: Scholl reaction gives Möbius topology, while InCl_3_‐mediated alkyne annulation gives Hückel topology. The Möbius N‐heterocycloarene is unevenly contorted as revealed by crystal structure, and the Hückel N‐heterocycloarene binds tetrafluoroborate with C–H···F hydrogen bonds in solution

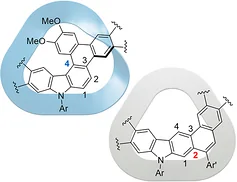

## 1 Introduction

**FIGURE 1:**
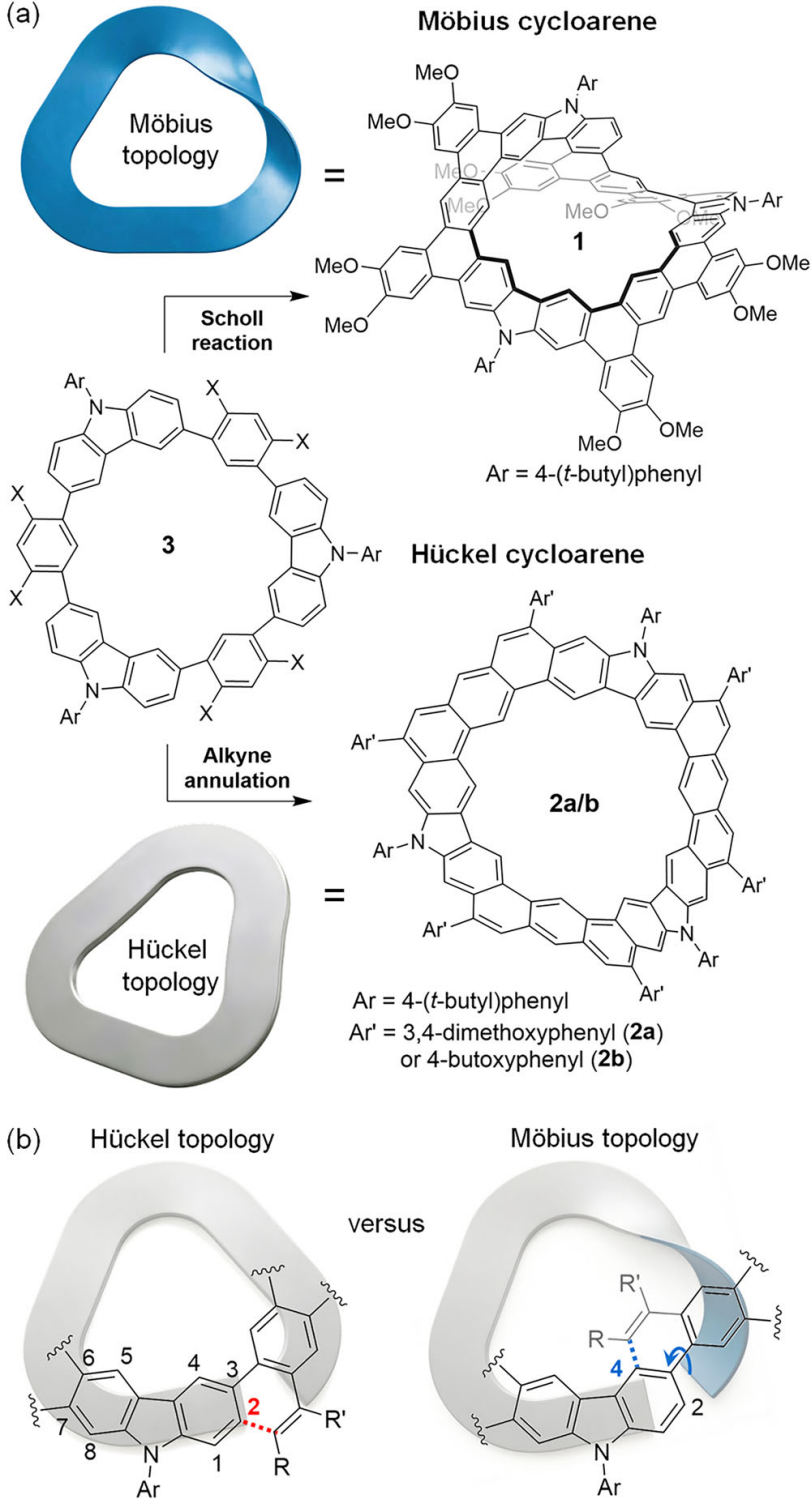
(a) Structures of Möbius and Hückel N‐heterocycloarenes and their synthetic precursor. (b) Schematic showing the regioselective C–C bond formation on a 3,6‐carbazolylene unit and the resulting topological outcomes.

Cycloarenes are macrocyclic polycyclic aromatic hydrocarbons composed of fully fused benzene rings and featuring inward‐pointing C–H bonds [1, 2, 3]. The first and most iconic member of this class is kekulene, synthesized by Staab and Diederich in 1978 [4]. Early studies showed that π‐electron delocalization [5] in these systems follows Clar's aromatic sextet model [6], as evidenced by deshielded inner protons in ^1^H NMR spectra, rather than global annulene‐type aromaticity [7]. Replacing one or more benzenoid rings in cycloarenes with heterocycles results in heterocycloarenes [8, 9]. Beyond their historical role in aromaticity studies, cycloarenes and heterocycloarenes have attracted significant interest owing to their macrocyclic conjugated π‐systems and intrinsic molecular cavities. These features offer a compelling combination of synthetic challenge, structural elegance, and promising applications in organic electronics, supramolecular chemistry, and optical materials [10]. They also serve as molecular models for topological defects in graphene [2] and may represent repeating units in periodic graphene nanomeshes [11].

(Hetero)cycloarenes feature conjugated inner and outer perimeters, and the relative lengths of these two perimeters determine their overall geometry. When the inner perimeter is contracted, an otherwise planar (hetero)cycloarene adopts a bowl‐ or cone‐shaped π‐system [12], as exemplified by [3]chrysaorole [8], and tribenzo[3]chrysaorole [13]. Conversely, expansion of the inner perimeter induces a saddle‐shaped distortion, as observed in septulene [14] and octulene [11]. These out‐of‐plane deformations cause the *p*‐orbitals that constitute the π‐system to deviate from ideal perpendicular alignment with respect to the macrocyclic mean plane, positioning such contorted (hetero)cycloarenes structurally between strictly cylindrical carbon nanobelts and fully planar cycloarenes. No matter whether a (hetero)cycloarene is flat or distorted like a bowl or saddle, its inner and outer rims are usually distinct, that is, they share no atoms. This situation can be changed by introducing a 180° twist, which gives rise to a Möbius loop (Figure 1). Due to the twist, a Möbius loop is characterized by a one‐sided, non‐orientable surface and possesses only a single rim. The intriguing geometry of Möbius loops has long fascinated mathematicians, artists, designers, and architects [15]. In chemistry, interest in Möbius topology began in 1964 when Heilbronner predicted that [4n]annulenes adopting a Möbius topology would display aromaticity [16], in contrast to the conventional expectations of Hückel's rule. Zimmerman subsequently extended the concepts of Möbius aromaticity and antiaromaticity to monocyclic orbital arrays featuring one or an odd number of sign‐inverted overlaps between adjacent orbitals [17]. This framework provides an alternative interpretation of pericyclic reaction transition states, complementing the Woodward–Hoffmann rules through the lens of Hückel and Möbius aromaticity [18]. Despite their structural interest and recent synthetic advances [19, 20, 21, 22, 23, 24, 25]. π‐conjugated molecules with Möbius topology or Möbius aromaticity remain challenging synthetic targets. Their construction is hampered by significant ring strain and molecular contortion, which disrupt *p*‐orbital overlap and can offset stabilization gained from Möbius aromaticity.

In this study, a Hückel (hetero)cycloarene is defined as a flat or nearly flat π‐system featuring *p*‐orbitals aligned perpendicular to the macrocyclic mean plane. This distinguishes it from a conjugated nanobelt [26], which exhibits radial *p*‐orbital alignment [27]. A Möbius (hetero)cycloarene is defined as a macrocycle formed by introducing a 180° twist into a Hückel (hetero)cycloarene, differentiating it from a Möbius nanobelt [28, 29], which arises from twisting a conjugated nanobelt. However, upon introducing the 180° twist, the resulting geometries of both Möbius (hetero)cycloarenes and Möbius nanobelts become highly similar because they are dominated by the topological distortion. Notably, Möbius (hetero)cycloarenes fully realize the Möbius stripe topology, characterized by both a one‐sided surface and a single‐edge conjugation pathway.

Herein, we report a Möbius N‐heterocycloarene (**1**) and a Hückel N‐heterocycloarene (**2**), synthesized from precursors that share the same [3]cyclo‐3,6‐carbazolylene‐1,3‐phenylene backbone (**3**), as shown in Figure 1a. The topological outcome is governed by the choice of the annulation reaction: the Möbius N‐heterocycloarene was accessed via a Scholl reaction, whereas the Hückel N‐heterocycloarene was obtained using an alkyne annulation. Detailed below are the syntheses of **1** and **2**, along with a comparative study of their molecular structures and electronic properties using experimental and computational methods.

## 2 Results and Discussion

A typical synthetic approach to Hückel (hetero)cycloarenes is stitching a single‐stranded macrocyclic precursor through an annulation reaction, either in a fold‐out or a fold‐in manner. In this study, we used [3]cyclo‐3,6‐carbazolylene‐1,3‐phenylene (**3**) derivatives as the single‐stranded macrocyclic precursors and envisioned that the topological outcome in the resulting N‐heterocycloarene would be dictated by the regioselectivity of C–C bond formation on the 3,6‐carbazolylene units in the annulation reaction, as shown in Figure 1b. Specifically, while forming all six new bonds at the C2 and C7 positions would yield a Hückel topology, introducing a single bond at the C4 (or C5) position alongside five bonds at C2/C7 would induce the requisite 180° twist for a Möbius architecture. This approach differs from reported syntheses of carbon nanobelts [26, 28] and their S‐ or O‐embedded analogues [29, 30], in which the nanobelt topology (Hückel or Möbius) is determined by the number of units (even or odd, respectively) in the macrocyclic precursors.

**SCHEME 1:**
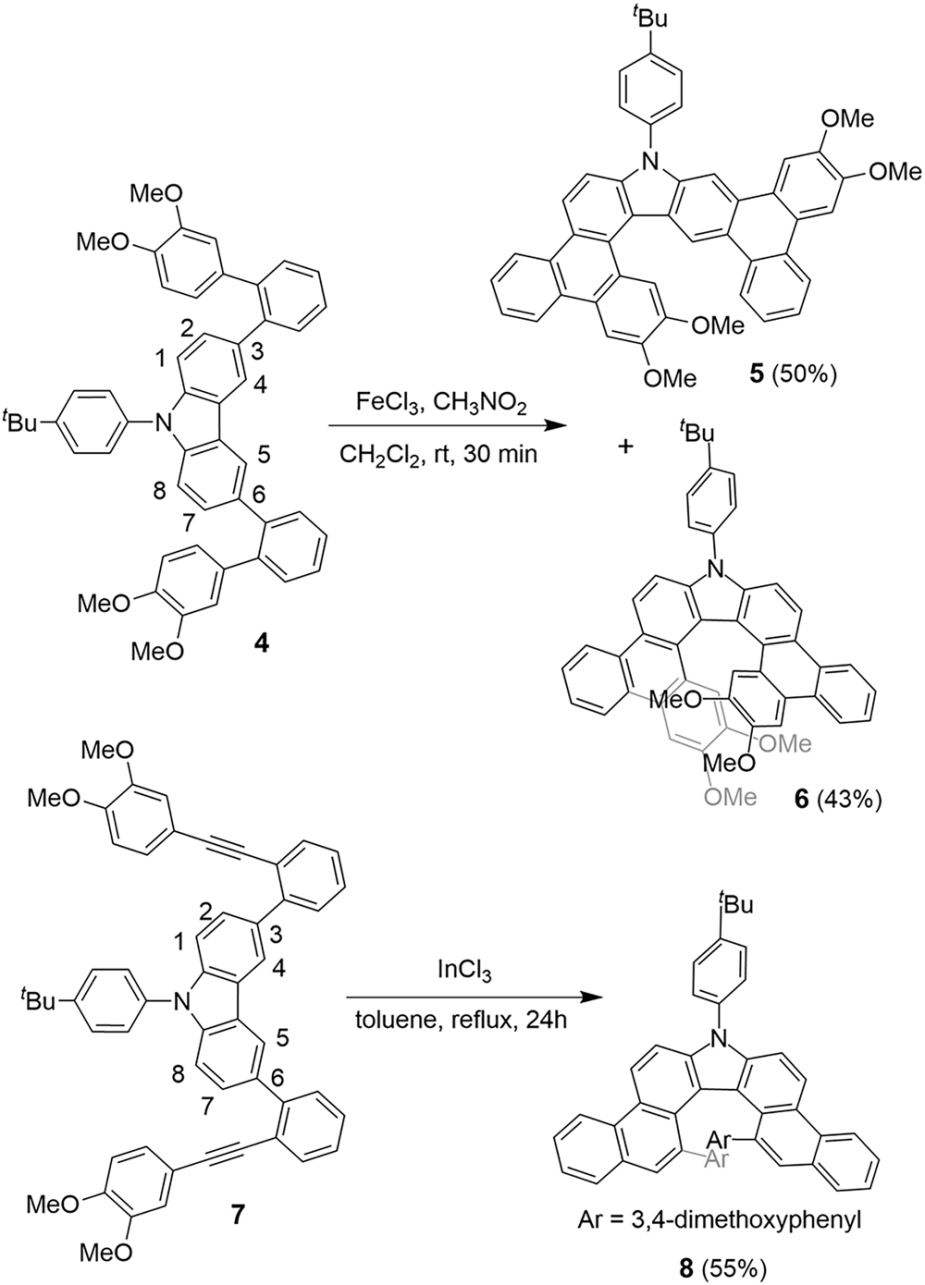
Scholl reaction of **4** and InCl_3_‐mediated alkyne annulation of **7**.

To understand which of these two bonding modes is intrinsically favored, we first examined two annulation methods, Scholl reaction [31, 32] and Lewis acid‐mediated alkyne annulation reaction, on acyclic model compounds that replicate the 3,6‐carbazolylene‐1,3‐phenylene motif but lack macrocyclic strain. The regioselectivity observed in these model systems would serve as a baseline for interpreting the outcomes of the macrocyclic precursors. Previously reported Scholl reactions of 3,6‐carbazolylene derivatives enable exclusive bond formation at the C4 and C5 positions. In this study, however, we found that the regioselectivity can be tuned using compound **4**, which bears two 3,4‐dimethoxyphenyl substituents. As illustrated in Scheme 1, the Scholl reaction of **4** with FeCl_3_ in CH_3_NO_2_ afforded compound **5** (50%), featuring new bonds formed on C2 and C5, alongside compound **6** (43%), formed via bond formation on C4 and C5. Similar product distributions were observed when 2, 3‐dichloro‐5, 6‐dicyano‐1, 4‐benzoquinone (DDQ) and CH_3_SO_3_H were used for the Scholl reaction. The structure of **5** was unambiguously identified with single crystal X‐ray crystallography [33], and the C_2_‐symmetric structure of **6** was determined using NMR spectroscopy. In contrast, the InCl_3_‐mediated alkyne annulation of compound **7** afforded compound **8** (55%) via bond formation at C4 and C5, as shown in Scheme 1. This reaction also produced a minor product (yield < 5% based on mass of impure material) that exhibited a smaller Rf value than **7** on thin‐layer chromatography. Mass spectrometry identified it as a constitutional isomer of **7**, likely featuring C2/C7 bond formation, but it could not be isolated in pure form for structural determination by NMR spectroscopy. These results indicate that both the Scholl reaction and the Lewis acid‐mediated alkyne annulation favor bond formation at C4/C5 in the acyclic model compounds, although the 3,4‐dimethoxyphenyl substituents increase the preference for C2/C7 coupling relative to previously reported Scholl reactions of 3,6‐carbazolylene derivatives. The lower yield of the alkyne annulation product (**8**) compared to the Scholl reaction products (**5 **and** 6**) can be attributed to steric repulsions between the two aryl groups in **8**, which make **8** more difficult to access.

**FIGURE 2:**
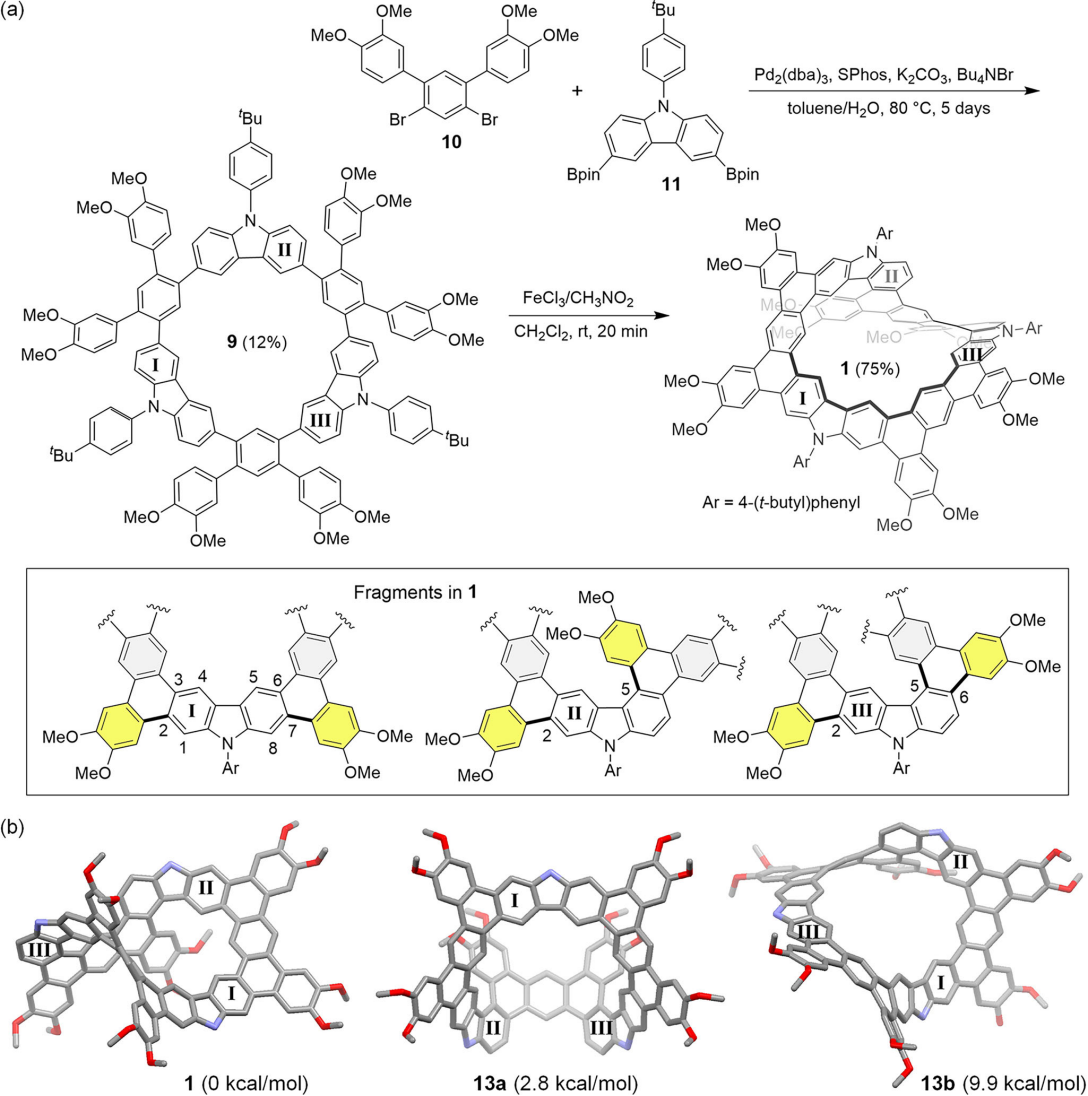
(a) Synthesis of Möbius N‐heterocycloarene **1**; (b) structures of **1** and its constitutional isomers **13a** and **13b** optimized at the B3LYP /6‐31g(d) level of DFT (the 4‐*t*‐butylphenyl groups and hydrogen atoms are removed for clarity).

The two annulation methods were then applied to the macrocyclic precursors. The Scholl reaction precursor **9** (Figure 2a) was constructed via a Suzuki coupling between dibromide **10** and diboronate **11**, which were prepared using modified literature procedures for analogous compounds [34]. From the crude product of macrocyclization, **9** was isolated in a yield of 12%, while other cyclic oligomers were not isolated. Subsequent screening of Scholl reaction conditions (including FeCl_3_/CH_3_NO_2_, DDQ/CH_3_SO_3_H and DDQ/CF_3_SO_3_H) revealed that treating **9** with 27.5 equivalents of FeCl_3_ in CH_3_NO_2_ at room temperature for 20 min was optimal, affording Möbius N‐heterocycloarene **1** in a yield of 75% via the formation of six C–C bonds. To elucidate the connectivity in **1**, the three carbazole units are labeled **I**, **II**, and **III** (Figure 2a). Single‐crystal x‐ray crystallography reveals distinct bonding patterns: carbazole **I** forms bonds at C2 and C7; carbazole **II** at C2 and C5; and carbazole **III** at C2 and C5, together with an unexpected skeletal rearrangement. The Möbius topology arises directly from how these attachment points govern the spatial orientation of the 3,4‐dimethoxyphenyl substituents (highlighted in yellow). In carbazole **II**, the macrocyclic linkage at C5 forces the attached 3,4‐dimethoxyphenyl group into the interior of the cavity. This inward positioning induces the requisite 180° twist for Möbius topology. In contrast, carbazole **III** undergoes a skeletal rearrangement involving the migration of the original C6–aryl bond to C5 and the formation of a new bond between C6 and the 3,4‐dimethoxyphenyl group. Crucially, this reorganization positions the 3,4‐dimethoxyphenyl group outside the macrocycle without introducing a twist. Consequently, the overall topological twist in **1** is 180°. The C–C bond formation in **1** indicates that embedding the 3,6‐carbazolylene units into the constrained macrocycle **9** imposes prohibitive strain on multiple C4/C5 linkages, forcing four bonds to form at C2/C7. We attribute the driving force for the rearrangement to strain release. This interpretation is supported by comparing the stability of **1** with its constitutional isomers **13a** and **13b** (Figure 2b), in which carbazole **III** forms bonds at C5 and C7, respectively, without rearrangement. Density functional theory (DFT) calculations indicate that **13a** and **13b** are higher in Gibbs free energy than **1** by 2.8 kcal/mol and 9.9 kcal/mol, respectively.

**SCHEME 2:**
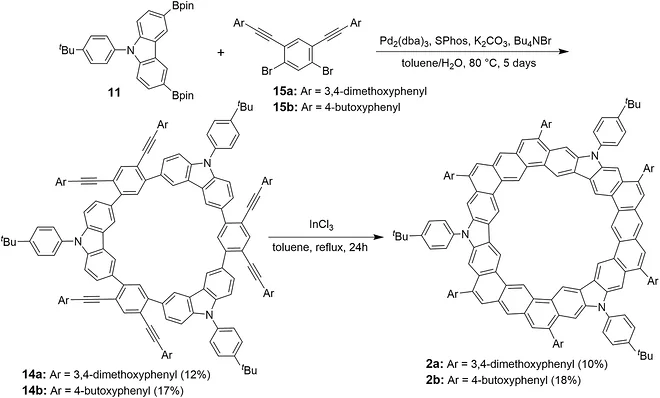
Synthesis of Hückel N‐heterocycloarenes **2a/b**.

The macrocyclic precursor for alkyne annulation, **14a**, was synthesized via a Suzuki coupling between diboronate **11** and dibromide **15** (Scheme 2) following the same procedure used for the synthesis of **9**. From the crude macrocyclization mixture, **14a** was isolated in 12% yield, while the cyclic tetramer and pentamer were also detected by mass spectrometry but not isolated. Treatment of **14a** with InCl_3_ in refluxing toluene afforded Hückel N‐heterocycloarene **2a** in 10% yield via the formation of three C–C bonds at the C2 positions and three C–C bonds at the C7 positions of the carbazole units. The ^1^H NMR spectrum of **2a** in the aromatic region exhibits six singlets and four doublets, consistent with a three‐fold symmetric structure, and these signals were assigned using NOESY 2D NMR spectra (Figures S38–S40 in the Supporting Information). However, purification by column chromatography followed by gel permeation chromatography on a preparative recycling HPLC system failed to remove trace impurities from **2a**, as evidenced by the ^1^H NMR spectrum, presumably due to the low solubility of **2a**. To address this issue, we synthesized another macrocyclic precursor, **14b**, bearing butyl substituents to enhance solubility. The InCl_3_‐mediated alkyne annulation of **14b** under the same conditions afforded N‐heterocycloarene **2b** in a higher yield (18%) and in pure form after the same purification procedures. The formation of **2a**/**2b** via six C–C bonds at the C2/C7 positions of the carbazole units indicates that the intrinsic regioselectivity of the InCl_3_‐mediated alkyne annulation is reversed by the constrained macrocyclic precursor, and to a greater extent than in the Scholl reaction, because positioning the 3,4‐dimethoxyphenyl or 4‐butoxyphenyl groups inside the macrocycle would result in intolerable repulsions and strain. However, unlike the Scholl reaction of the acyclic model compound, which already exhibits dual bonding modes (C2/C7 and C4/C5), the alkyne annulation reaction has a stronger intrinsic preference for bond formation at C4/C5. This makes bonding at C2/C7 less efficient, resulting in lower yields of **2a**/**2b**.

**FIGURE 3:**
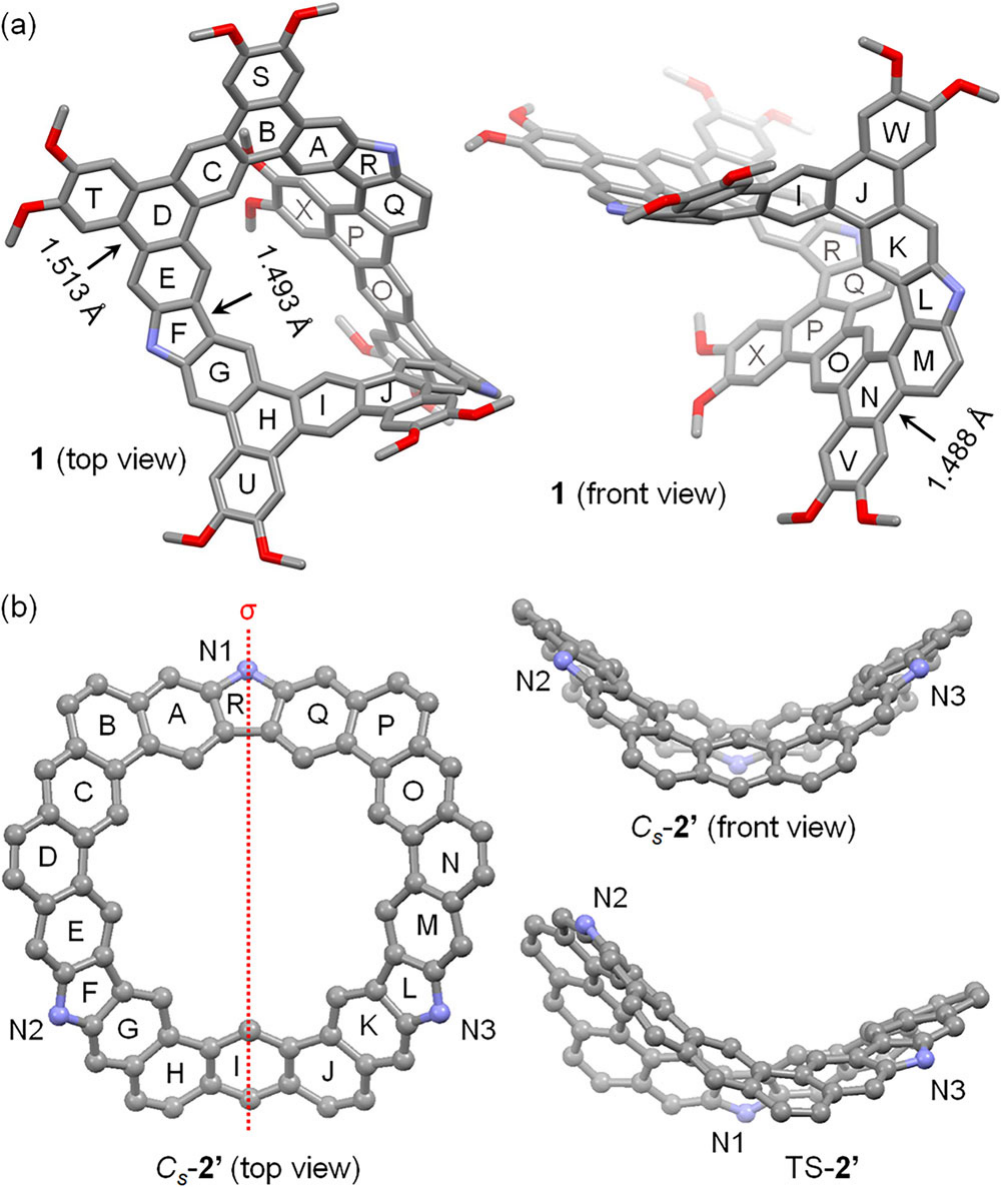
(a) Structure of (*P*)‐**1** in the crystal with the 4‐*t*‐butylphenyl groups and hydrogen atoms removed for clarity; (b) DFT‐calculated global minimum structure of **2’** (*C_s_*‐**2’**) and the transition state (TS‐**2’**) in the conformational change of **2’** with hydrogen atoms removed.

Single crystals of compound **1** were obtained by slow evaporation of a solution in hexane/CH_2_Cl_2_/CH_3_OH. X‐ray crystallography [31] revealed that the compound exists as a pair of enantiomers within the racemic crystal. Figure 3a shows the top and front views of (*P*)‐**1** with all benzenoid and pyrrole rings labeled. To understand how the 180° twist in the Möbius loop is distributed along the curved π‐backbone of **1**, the non‐planarity of each ring in (*P*)‐**1** was analyzed using this crystal structure. The non‐planarity of a ring is defined as the average distance of its atoms from a calculated best‐fit plane [35]. Thus, a more curved ring has a larger non‐planarity value. Table 1 summarizes the non‐planarity values for individual rings in (*P*)‐**1**, indicating that the contortion in the Möbius loop is not equally shared among rings A–R. Rings H, I, and J show the greatest contortion, with non‐planarity values ranging from 0.086 to 0.108, followed by rings M–Q (0.054–0.076). In contrast, rings E and F are nearly flat, with values of 0.013 and 0.018, respectively. As expected, rings S–X are almost flat, with small non‐planarity values (0.007–0.013), as they are not essential parts of the Möbius loop.

**TABLE 1:** Non‐planarity^a^ and HOMA^b^ values for individual rings in (*P*)‐**1**.

Ring	Non‐planarity	HOMA	Ring	Non‐planarity	HOMA
A	0.043	0.719	M	0.054	0.703
B	0.037	0.238	N	0.064	0.110
C	0.044	0.647	O	0.054	0.850
D	0.045	−0.376	P	0.067	−0.120
E	0.013	0.847	Q	0.061	0.901
F	0.018	—	R	0.032	—
G	0.024	0.823	S	0.012	0.838
H	0.094	0.317	T	0.008	0.866
I	0.086	0.819	U	0.013	0.735
J	0.108	−0.063	V	0.007	0.913
K	0.046	0.790	W	0.008	0.858
L	0.034	—	X	0.008	0.765

^a^ Non‐planarity values were calculated by constructing a best‐fit plane through the individual rings and measuring the average distance of the carbon atoms from this plane.

^b^ HOMA = 1 – (*α*/*n*)∑(*R*_opt_ – *R*_i_)^2^, where *n* is the number of bonds taken into the summation, *α* = 257.7 is an empirical constant chosen to give HOMA = 0 for the hypothetical Kekulé structures with alternation of single and double bonds and HOMA = 1 for the system with all bond lengths equal to the optimal value *R*_opt_ = 1.388 Å, and *R_i_* is the individual bond length in the ring.

Using bond lengths measured from the crystal structure, we also analyzed the local aromaticity of individual carbocycles in (*P*)‐**1** via the harmonic oscillator model of aromaticity (HOMA) [36, 37, 38], which assesses aromaticity based on bond length equalization. Table 1 summarizes the HOMA values, showing that rings B, H, and N have low positive HOMA values, while rings D, J, and P have negative values. These values indicate that these rings have low aromaticity or are non‐aromatic, as a result of long C–C bonds. In particular, as shown in Figure 3a, rings D and N exhibit C–C bond lengths of 1.513 and 1.487 Å, respectively, which are even longer than the typical non‐conjugated single bond between two *sp* ^2^‐hybridized carbon atoms (1.47–1.48 Å) [39]. Another notably long C–C bond (1.493 Å) is found in ring F. In contrast, the other six‐membered carbocycles have high HOMA values in the range 0.647–0.913, indicating that these rings are aromatic. These results are consistent with predictions based on Clar's rule: rings A, C, E, G, I, K, M, O, Q, S, T, U, V, W, and X are aromatic sextets, while rings B, D, H, J, P, and N are “empty.” No clear correlation between planarity and aromaticity is observed in **1**: both planar (e.g., E) and contorted (e.g., I) rings can have high HOMA values, and similarly, both aromatic (e.g., I) and non‐aromatic (e.g., P) rings can exhibit high non‐planarity.

Because our attempts to grow crystals of **2a** and **2b** suitable for single‐crystal x‐ray diffraction were unsuccessful, the molecular geometry and stereodynamics of **2a** and **2b** were studied using DFT calculations at the B3LYP/6‐31G(d) level on a simplified model molecule (**2’**), in which all aryl substituents were replaced by hydrogen atoms. Geometry optimization reveals that **2’** adopts a saddle‐shaped geometry at the global minimum, which possesses *C_s_* symmetry with the symmetry plane passing through the nitrogen atom N1 (Figure 3b). Through a transition state (TS‐**2’** in Figure 3b) that has a slightly twisted saddle geometry, *C_s_*‐**2’** converts into two other conformers that have the same geometry and Gibbs free energy, with their symmetry planes passing through nitrogen atoms N2 and N3, respectively. This transition state has an energy barrier of only 2.8 kcal/mol, corresponding to a rate constant of 5.5 × 10^10^ S^−1^ at 25 °C, as estimated using the Eyring equation with a transmission coefficient of unity. This extremely fast conformational change occurs through a pseudorotation, which involves a continuous wavelike motion analogous to that of [7]circulene [40, 41]. The three‐fold symmetry observed in the NMR spectra of **2a** and **2b** should therefore be attributed to rapid conformational interconversion, which yields a time‐averaged structure with three‐fold symmetry.

**FIGURE 4:**
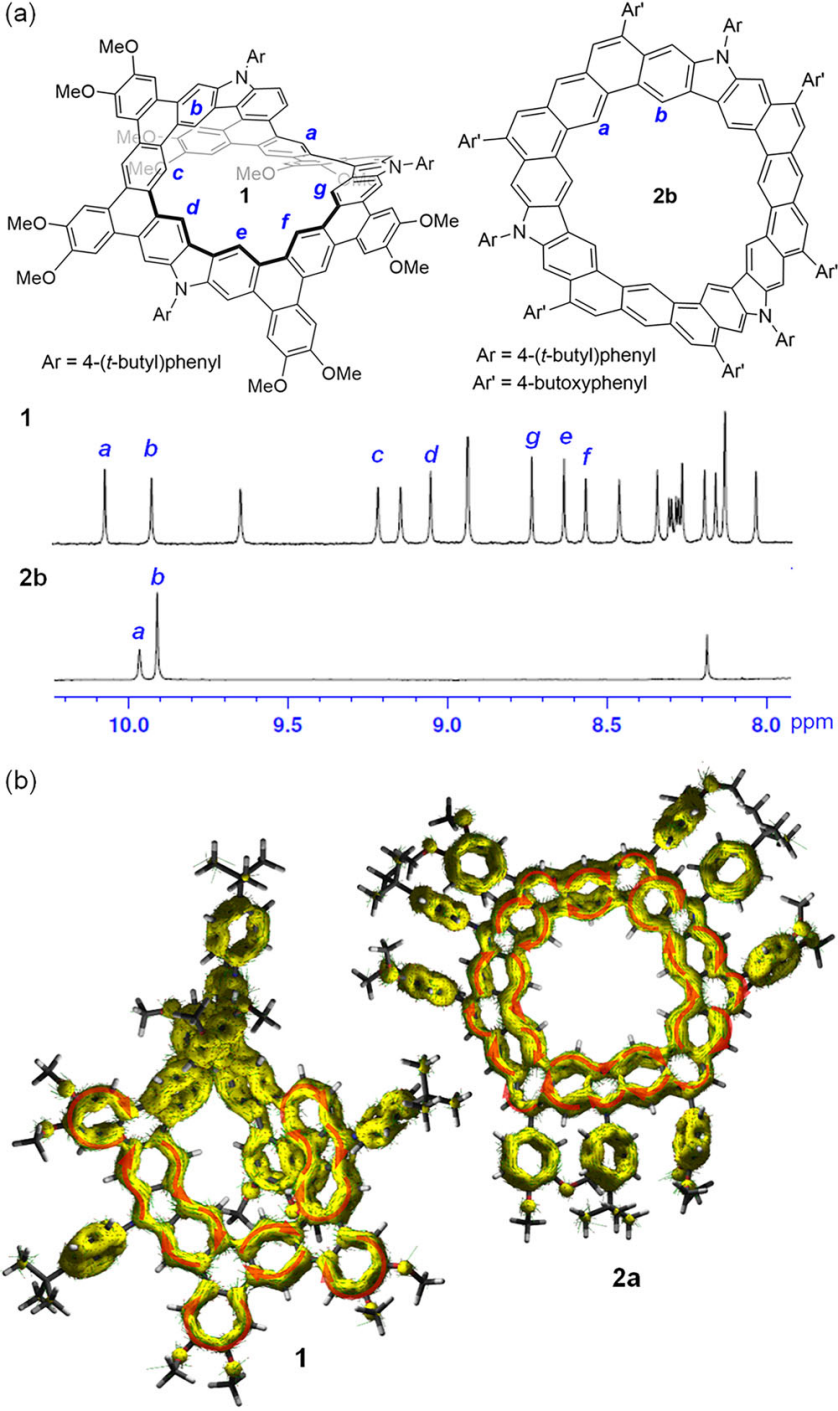
(a) Partial ^1^H NMR spectra of **1** and **2b** (molecular structures shown with labels); (b) calculated ACID maps of **1** and **2a** (isovalue = 0.04) based on π‐electron contributions under an external magnetic field oriented perpendicular to the plane and pointing outward.

One notable observation from the ^1^H NMR spectra of N‐heterocycloarenes **1** and **2a**/**b** is that the chemical shifts of protons *c*–*g* in **1** are shifted upfield by 0.7–1.5 ppm relative to protons *a* and *b* in **2a**/**b** (Figure 4a), even though these protons have similar local chemical environments. To investigate whether these upfield shifts are related to global ring currents along the macrocycles, the aromaticity of **1** and **2a** was studied theoretically using nucleus‐independent chemical shift (NICS) and anisotropy of the induced current density (ACID) calculations, both of which assess aromaticity based on magnetic criteria. In **1**, rings B, D, H, J, N, and P (ring labeling as in Figure 3a) exhibit small NICS(0) values, ranging from −0.15 to −0.5 ppm, whereas the other benzenoid rings exhibit more negative NICS(0) values from −8.0 to −10.4 ppm, and the pyrrole rings show values from −7.3 to −8.0 ppm. These results indicate that rings B, D, H, J, N, and P are nonaromatic while the remaining rings are aromatic, consistent with the HOMA values derived from bond lengths in the crystal structure and with the aromatic sextets predicted by Clar's rule. Similarly, in **2a** (ring labeling in Figure 3b), rings B, D, H, J, N, and P are weakly aromatic, with NICS(0) values ranging from –4.3 to –3.8 ppm; and the other benzenoid rings are aromatic, with more negative NICS(0) values (–10.0 to –10.6 ppm), again in agreement with Clar's rule. The pyrrole rings (F, L, R) in **2a** have NICS(0) values of –6.7 to –6.8 ppm. In contrast, the NICS(0) values at the macrocycle centers of **1** and **2a** are 0.6 and 2.5 ppm, respectively, indicating the absence of global ring currents. Figure 4b presents the ACID maps of **1** and **2a**, calculated with the external magnetic field perpendicular to the paper plane. These maps show that diamagnetic ring currents are mainly localized in the carbazole units and in benzenoid rings (C, I, and O in both compounds, as well as S–X in **1**). Nevertheless, weak ring‐current flows are also observed between these localized aromatic units (see additional ACID maps with different isovalues in Figures S52), implying that the π‐electrons are globally delocalized along the macrocycle to a small extent, similar to that reported for expanded kekulenes [42, 43]. Because the NICS and ACID calculations reveal localized ring currents on the carbazole and benzene units, we attribute the upfield chemical shifts in **1** not to global ring currents in **1** or **2a/b**, but to the highly contorted geometry of **1**, which positions protons c–f in the shielding region of rings O–R.

**FIGURE 5:**
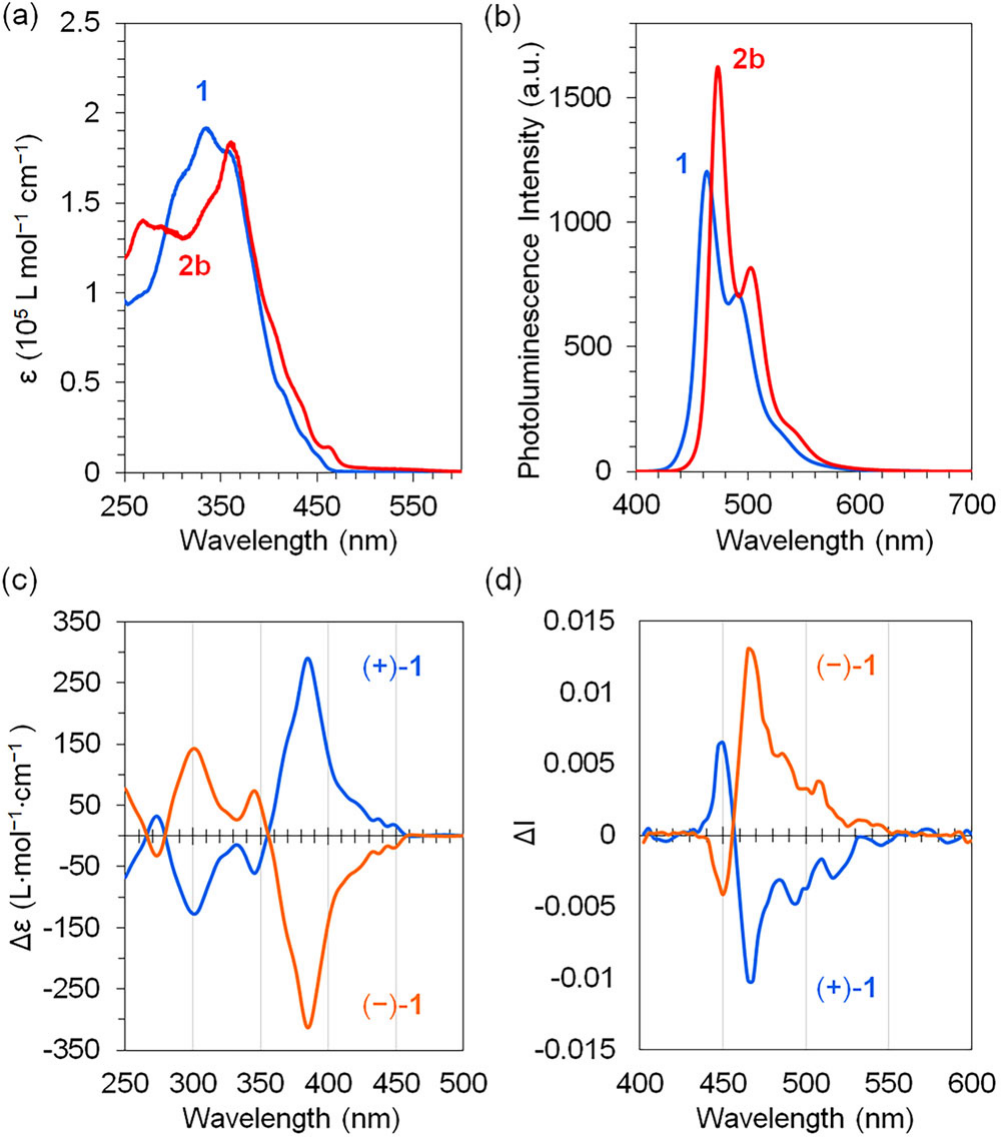
(a) UV‐vis absorption of **1** and **2b** in CH_2_Cl_2_; (b) photoluminescence spectra of **1** and **2b** in CH_2_Cl_2_ excited at 334 and 360 nm, respectively; (c) CD spectra of (+)‐**1** and (−)‐**1** in CH_2_Cl_2_; (d) CPL emission spectra of (+)‐**1** and (−)‐**1** in CH_2_Cl_2_ excited at 300 nm (All the solutions have the same concentration of 1 × 10^−5^ mol/L).

The photophysical properties of Möbius and Hückel N‐heterocycloarenes (**1** and **2b**, respectively) were studied using UV‐vis absorption and photoluminescence spectroscopy, while the chiroptical properties of **1** were investigated using circular dichroism (CD) and circularly polarized luminescence (CPL) spectroscopy. Figure 5a,b compares the absorption and photoluminescence spectra of **1** and **2b**, demonstrating that they have similar absorption and emission properties. This similarity indicates that the twist of the π‐backbone in **1** has a minor effect on the overall electronic properties, and that the electronic structures of **1** and **2b** are mainly controlled by local π‐conjugation rather than global delocalization through the macrocycles, in agreement with the localized aromatic rings found from HOMA and NICS analysis. Using chiral HPLC, compound **1** was resolved into two enantiomers, which exhibit electronic circular dichroism in the wavelength range of 250−450 nm (Figure 5c). On the basis of DFT‐calculated CD spectra (Figure S52), (+)‐**1** and (−)‐**1** are assigned to (*P*)‐**1** and (*M*)‐**1**, respectively. The CD spectrum of (−)‐**1** exhibits a maximum |Δ*ε*| value of 296 L·mol^−1^·cm^−1^ at 388 nm. The corresponding absorption dissymmetry factor, *g*_abs_ = |Δ*ε*|/*ε*, is calculated as 3.0 × 10^−3^. The optically pure forms of **1** also exhibit circularly polarized luminescence (CPL) activity in the wavelength range of 440–550 nm (Figure 5d), with a luminescence dissymmetry factor (*g*_lum_) of approximately 2 × 10^−3^. The dissymmetry factors (*g*_abs_ and *g*_lum_) of **1** are on the typical order of 10^−3^ for chiral π‐conjugated molecules [43, 44].

**FIGURE 6:**
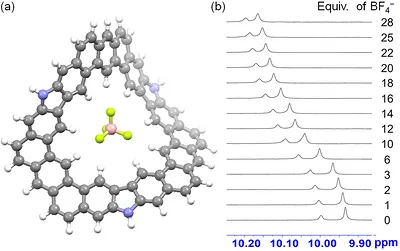
(a) Structure of **2’**⊃BF_4_^−^ optimized at the ωB97XD/6‐31+G(d,p) level of DFT in vacuo. (b) Changes in the ^1^H NMR spectrum of **2b** (2 × 10^−3^ M) CD_2_Cl_2_/CS_2_ (volume ratio 2:1) during titration with tetrabutylammonium tetrafluoroborate.

Compound **2a/b** has a large cavity surrounded by nine C(*sp* ^2^)‐H bonds pointing approximately toward its center. In principle, it can therefore act as a host for anions, similar to octulene, which can accommodate a chloride anion [11]. DFT calculations suggest that model compound **2’** can bind tetrafluoroborate (BF_4_^−^) through C–H···F hydrogen bonds (Figure 6a), with nine H···F contacts ranging from 2.26 to 2.38 Å, which are shorter than the sum of the van der Waals radii of hydrogen and fluorine (2.56 Å). The ability of **2b** to bind BF_4_^−^ was investigated using NMR titration. Upon addition of 28 equivalents of tetrabutylammonium tetrafluoroborate to a solution of **2b** (2 × 10^−3^ M in CD_2_Cl_2_/CS_2_, volume ratio 2:1), the protons *a* and *b* (inside the cavity, see Figure 4a) shifted downfield by approximately 0.2 ppm (Figure 6b), indicating that **2b** binds BF_4_^−^ through hydrogen bonds, with a fast exchange between the free host and the complex on the NMR time scale. Fitting the data to a 1:1 binding model using BindFit [45, 46] gave a binding constant of 11 M^−1^ with an error of 4.0%. This low binding constant is likely due to strong solvation by CD_2_Cl_2_ and CS_2_, which were necessary to enhance solubility and prevent aggregation of **2b**. For comparison, octulene was reported to bind chloride in benzene containing 1% CD_2_Cl_2_ with a binding constant of 2.2 × 10^4^ M^−1^ [11].

## 3 Conclusion

In conclusion, this study presents the divergent synthesis of a Möbius N‐heterocycloarene (**1**) and a Hückel N‐heterocycloarene (**2a/b**) from a common single‐stranded macrocycle, [3]cyclo‐3,6‐carbazolylene‐1,3‐phenylene. This macrocycle is shared by precursors **9** and **14a/b**, which undergo different annulation reactions to afford **1** and **2a/b**, respectively. Critically, the topological outcome is dictated by the regioselectivity of C–C bond formation on the carbazolylene units. This contrasts with prior syntheses of carbon nanobelts [26, 28] and their S‐ or O‐embedded analogues [29, 30], where topology (Hückel or Möbius) is determined by the number of units (even or odd, respectively) in the macrocyclic precursor. The Scholl reaction of **9** afforded **1** by forming three C–C bonds at the C2 positions (one per carbazole unit), one bond at a C7 position, one bond at a C5 position that introduces the 180° twist required for the Möbius loop topology, and another bond at a different C5 position that is accompanied by a skeletal rearrangement. In contrast, the InCl_3_‐mediated alkyne annulation of **14a** and **14b** afforded **2a** and **2b**, respectively, by forming three C–C bonds at C2 and three C–C bonds at C7 of the carbazole units. Notably, both the Scholl reaction and the Lewis acid‐mediated alkyne annulation favored bond formation at the C4/C5 positions in acyclic model compounds. Therefore, the preference for C–C bond formation at the C2 and C7 positions in the synthesis of **1** and **2a/b** is driven by the constrained macrocyclic precursors, which reverse the intrinsic regioselectivity by imposing prohibitive strain.

Analysis of the crystal structure of **1** reveals that the contortion in the Möbius loop is not equally distributed among the 18 rings, and no clear correlation between planarity and aromaticity is observed. DFT calculations indicate that the N‐heterocycloarene backbone in **2a/b** adopts a saddle‐shaped geometry with *C*_s_ symmetry and exhibits high flexibility with rapid conformational change. NICS and ACID calculations reveal localized ring currents on the carbazole and benzene units in both **1** and **2a/b**, consistent with the aromatic sextets predicted by Clar's rule. As a result, the observed upfield chemical shifts in **1** relative to **2a/b** are attributed to the highly contorted geometry of **1**, which positions the upfield‐shifted protons within the shielding region of remote rings. The enantiopure form of **1** exhibits absorption and emission dissymmetry factors on the order of 10^−3^, which is typical for chiral π‐conjugated molecules. With a hydrogen‐surrounding cavity, compound **2b** demonstrates the ability to bind tetrafluoroborate with C–H···F hydrogen bonds in solution.

## Author Contributions

**Han Chen**: investigation, validation, formal analysis, writing – original draft, conceptualization. **Huishu Ma**: investigation, validation, formal analysis, writing – original draft. **Daiyue Yang**: investigation. **Zheng Zhou**: investigation, validation, formal analysis. **Xiao Chen**: investigation. **Zhifeng Huang**: resources. **Qian Miao**: conceptualization, formal analysis, supervision, funding acquisition, visualization, project administration, resources, writing – review and editing, writing – original draft.

## Conflicts of Interest

The authors declare no conflicts of interest.

## Supporting information




**Supporting File 1**: anie73496‐sup‐0001‐SuppMat.pdf.

## Data Availability

The data that support the findings of this study are available in the Supporting Information of this article.
