# Supplementary material for: Divergent Synthesis of Möbius and Hückel N‐Heterocycloarenes From a Common Macrocyclic Backbone
Source: Angew Chem Int Ed Engl. 2026 Jul 7;65(37):e2790811. doi: 10.1002/anie.2790811 (PMC13548944; doi:10.1002/anie.2790811)
Supplement: Supplementary file 1 [file ANIE-65-e2790811-s001.pdf]

Supporting Information for

Divergent Synthesis of Möbius and Hückel N-Heterocycloarenes from a Common Macrocyclic Backbone

Han Chen,[a, #] *Huishu Ma,*[a, #] *Daiyue Yang,*[b] *Zheng Zhou,*[c,*] *Xiao Chen,*[a] *Zhifeng Huang,*[a, d] *Qian Miao*[a, e,*]

[a] Department of Chemistry and [e] Shanghai-Hong Kong Joint Laboratory in Chemical Synthesis, The Chinese University of Hong Kong, Shatin, New Territories, Hong Kong, China

[b] Shanghai-Hong Kong Joint Laboratory in Chemical Synthesis, Shanghai Institute of Organic Chemistry, Chinese Academy of Sciences, 230032 Shanghai, China

[c] School of Materials Science and Engineering, Tongji University, Shanghai, 201804, China

[d] Shenzhen Research Institute, The Chinese University of Hong Kong, No.10, 2nd Yuexing Road, Nanshan, Shenzhen 518057, Guangdong Province, China

[e] Shanghai-Hong Kong Joint Laboratory in Chemical Synthesis, The Chinese University of Hong Kong, Shatin, New Territories, Hong Kong, China

[#] H. Chen and H. Ma contributed equally to this work.

* E-mail of the corresponding authors: zhouzheng@tongji.edu.cn, miaoqian@cuhk.edu.hk

Table of Contents

1. Synthesis
2. NMR spectra
3. High-resolution mass spectra
4. Photophysical properties
5. Chiral resolution
6. X-ray crystallography data
7. Computational studies
8. NMR titration experiments
9. References

1. Synthesis

General: The reagents and starting materials employed were commercially available and used without any further purification or made following reported methods as indicated. Unless otherwise noted, all reactions were performed with dry solvents under an atmosphere of nitrogen in dried glassware with standard vacuum-line techniques. Anhydrous and oxygen-free THF and CH_2Cl_2 were purified by an Innovative Technology Pure-Solv PS-MD- 4 system. Gel permeation chromatography (GPC) were performed on a JAI LC-9160 II NEXT automatic recycling preparative HPLC system with a UV/VIS detector. NMR spectra were recorded on a Bruker AVANCE III 400MHz spectrometer (^{1}H NMR: 400 MHz, ^{13}C NMR: 101 MHz). Abbreviations: s = singlet, d = doublet, t = triplet, m = multiplet. Chemical shift values (δ) are expressed in parts per million using residual solvent protons (^{1}H NMR, δ_H = 5.32 for CD_2Cl_2, δ_H = 7.26 for $CDCl_3$, ^{13}C NMR, δ_c = 53.84 for CD_2Cl_2, δ_C = 77.16 for $CDCl_3$) as internal standard. Mass spectra were recorded on Thermo Q Exactive Focus Orbitrap Mass spectrometer or a Bruker Autoflex speed MALDI-TOF spectrometer. UV-vis absorption spectra were recorded on a Shimadzu UV-3600 Plus UV-VIS-NIR Spectrophotometer. Fluorescence spectra were taken on a HITACHI F-4500 spectrofluorometer. Unless otherwise noted, melting points, without correction, were measured using a Nikon Polarized Light Microscope ECLIPSE 50i POL equipped with an INTEC HCS302 heating stage.

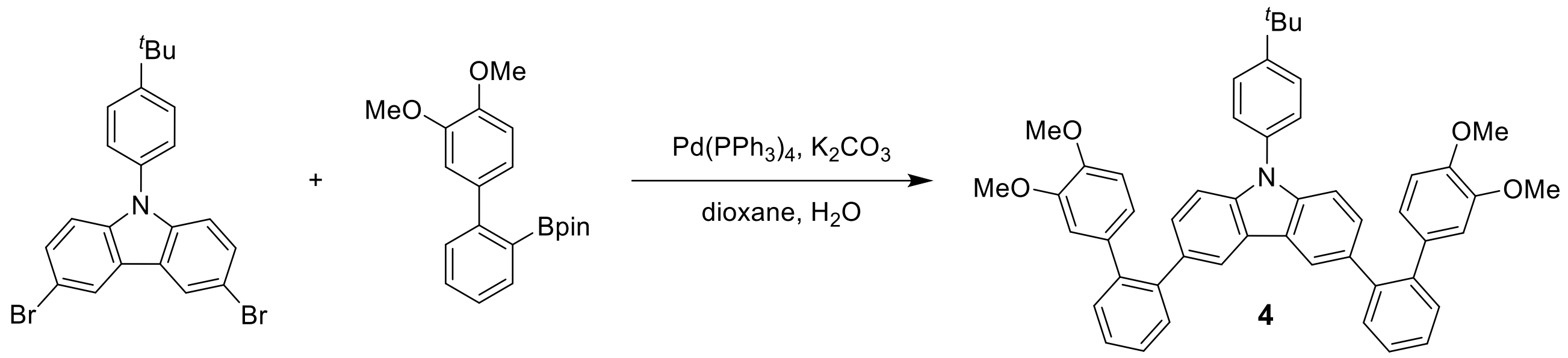

Compound 4

To a mixture of 3,6-dibromo-9-(4-*tert*-butylphenyl)-9H-carbazole[1] (100 mg, 0.22 mmol), 2-(3',4'-dimethoxy-1,1'-biphenyl-2-yl)-4,4,5,5-tetramethyl-1,3,2-dioxaborolane[2] (186 mg, 0.54 mmol), $Pd(PPh_3)_4$ (26 mg, 0.022 mmol) and K_2CO_3 (304 mg, 2.2 mmol) under a nitrogen atmosphere were added 5 ml of dioxane and 5 ml of water, both of which were bubbled with a flow of N_2 for 20 minutes prior to use. The reaction flask was then sealed and the reaction mixture was heated at 100°C overnight. The reaction mixture was cooled to room temperature, concentrated under a reduced pressure. The residue was then diluted with dichloromethane, washed with brine and the organic layer was dried with anhydrous Na_2SO_4. After concentrated under a reduced pressure, the crude product was purified by column chromatography on silica gel using hexane/CH_2Cl_2 (1/1 to 1/5, v/v) as eluent for step-gradient elution to give **4** (112 mg, 71%) as white solids. mp: 153-155 °C ^{1}H NMR (400 MHz, $CDCl_3$) δ (ppm): 7.98 (d, *J* = 1.1 Hz, 2H), 7.57-7.40 (m, 12H), 7.22 (d, *J* = 8.5 Hz, 2H), 7.07 (dd, *J* = 8.5, 1.6 Hz, 2H) 6.75 (d, *J* = 8.3 Hz, 2H), 6.60 (d, *J* = 1.8 Hz, 2H), 3.82 (s, 6H), 3.45 (s, 6H), 1.40 (s, 9H). ^{13}C NMR

(101 MHz, $CDCl_3$) δ (ppm): 150.53, 148.08, 147.66, 141.23, 140.45, 140.05, 134.85, 134.46, 133.79, 131.20, 130.48, 128.39, 127.38, 127.28, 126.83, 126.41, 123.32, 122.07, 121.17, 113.77, 110.78, 109.40, 55.81, 55.58, 34.87, 31.52. HRMS (APCI): calcd. for $C_{50}H_{45}NO_4$ ($[M+H]^+$): 724.34214, found: 724.34127.

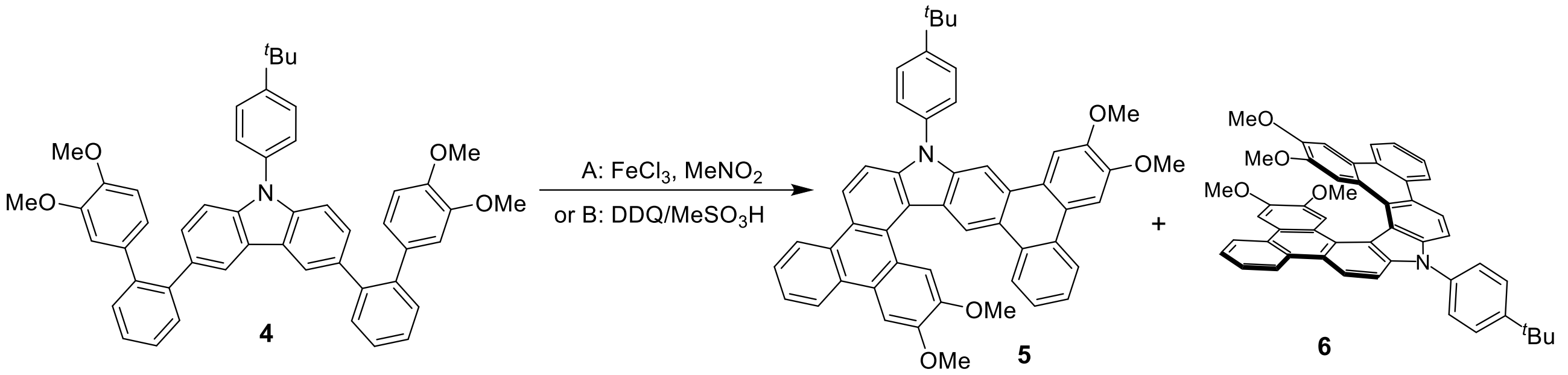

Method A: Compound **4** (10 mg, 14 μmol) was added to a reaction flask and the flask was purged with nitrogen three times. Anhydrous dichloromethane (10 mL) was then added to the reaction flask. $FeCl_3$ (22mg, 136 μmol) dissolved in 0.1 mL of nitromethane was quickly added to the above solution. After stirring for 30 minutes at room temperature, triethylamine was added to quench the reaction and the reaction mixture was poured into water. The organic layer was washed with brine and dried with anhydrous Na_2SO_4. After concentrated under reduced pressure, the mixture was purified by column chromatography on silica gel with hexane/CH_2Cl_2/diethyl ether (5:2:1, v/v) as eluent to isolate compound **5** (5.0 mg, 50%) and compound **6** (4.3 mg, 43%).

Method B: To a solution of **4** (10 mg, 14 μmol) and 2,3-dichloro-5,6- dicyanobenzoquinone (7 mg, 0.03 mmol) in 10 ml of anhydrous dichloromethane was added methanesulfonic acid (0.1 ml, 1.54 mmol) under an atmosphere of nitrogen. The reaction mixture was kept stirred at room temperature under the same atmosphere of nitrogen for 30 minutes and then quenched with triethylamine. The mixture was purified according to the same procedure as described above. Pure compound **5** (4.8 mg, 48%) and compound **6** (4.7 mg, 47%) were isolated.

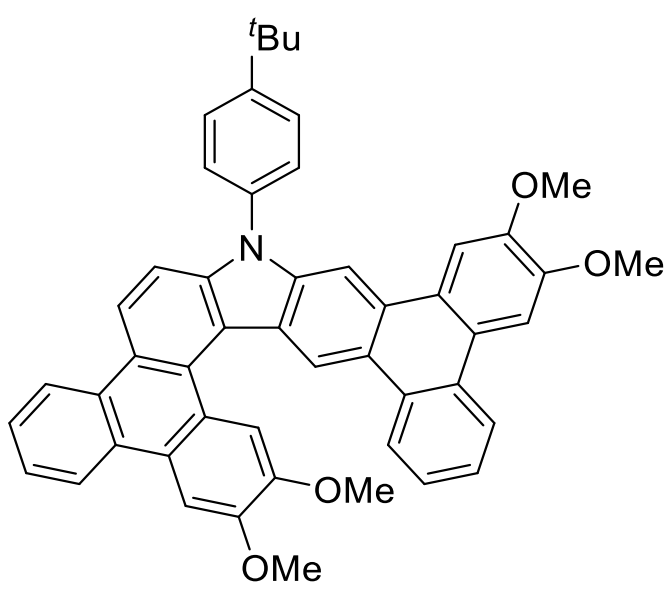

Compound 5

Pale yellow solids. mp: >300 °C ^1H NMR (400 MHz, $CDCl_3$) δ (ppm): 10.05 (s, 1H), 8.97 (s, 1H), 8.63-8.59 (m, 4H), 8.49 (d, *J* = 7.5 Hz, 1H), 8.36 (s, 1H), 8.15 (s, 1H), 7.99 (s, 1H), 7.84 (s, 1H), 7.75-7.60 (m, 9H), 4.28 (s, 3H), 4.14 (s, 3H), 4.03 (s, 3H), 3.79 (s, 3H), 1.56 (s, 9H). ^{13}C NMR (101 MHz,

$CDCl_3$) δ 151.47, 149.76, 149.61, 149.35, 148.00, 143.42, 141.52, 134.79, 130.29, 130.21, 128.94, 128.76, 128.30, 127.52, 127.46, 127.18, 127.01, 126.61, 126.31, 126.02, 125.52, 124.53, 124.34, 124.27, 124.05, 123.53, 123.03, 122.86, 122.73, 122.39, 122.01, 118.01, 117.23, 110.06, 109.71, 105.63, 104.96, 104.90, 102.23, 56.33, 56.22, 56.13, 55.98, 35.11, 31.64. HRMS (APCI): calcd. for $C_{50}H_{41}NO_4$ ($[M+H]^+$): 720.31084, found: 720.30950.

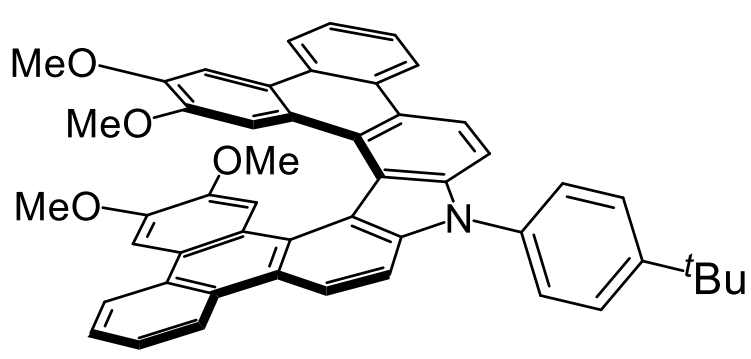

Compound 6

Pale yellow solids. mp: 173-175 °C ^{1}H NMR (400 MHz, $CDCl_3$) δ (ppm): 8.74 (d, J = 9.0 Hz, 2H), 8.71-8.69 (m, 2H), 8.59-8.56 (m, 2H), 7.86 (s, 1H), 7.79 (d, J = 8.8 Hz, 2H), 7.77-7.70 (m, 4H), 7.67-7.65 (m, 4H), 7.20 (s, 2H), 4.04 (s, 6H), 2.40 (s, 6H), 1.52 (s, 9H). ^{13}C NMR (101 MHz, $CDCl_3$) δ (ppm): 151.72, 148.92, 146.37, 141.17, 134.46, 129.82, 129.02, 127.95, 127.19, 126.96, 126.25, 125.94, 125.37, 123.81, 123.61, 123.29, 123.04, 121.36, 117.62, 109.97, 108.34, 103.62, 55.85, 54.06, 35.13, 31.65. HRMS (APCI): calcd. for $C_{50}H_{41}NO_4$ ($[M+H]^+$): 720.31084, found: 720.30960.

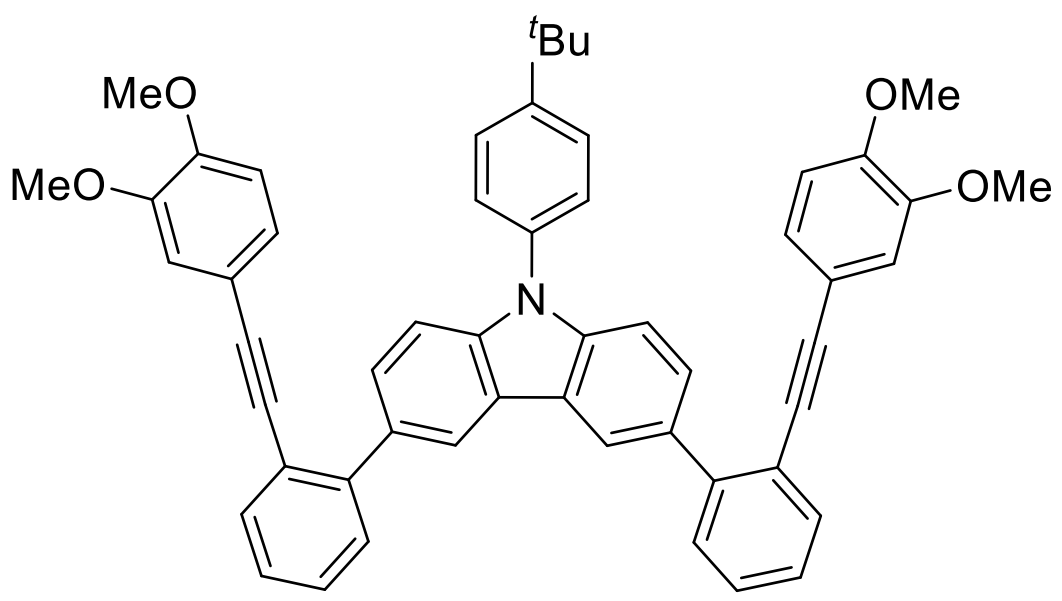

Compound 7

To a flask containing 144 mg (0.46 mmol) of 4-[2-(2-Bromophenyl)ethynyl]-1,2-dimethoxybenzene[3], 100 mg (0.18 mmol) of **11**, 753 mg (5.45 mmol) of K_2CO_3 and 17 mg (0.05 mmol) of tetra-*n*-butylammonium bromide were added 16 mL of toluene and 1 mL of H_2O. The reaction mixture was then bubbled with a flow of nitrogen for 15 minutes prior to use. 25 mg (0.26 mmol) of $Pd_2(dba)_3$ and 22 mg (0.05 mmol) of SPhos were then added to the reaction mixture. The reaction mixture was further bubbled with nitrogen for 30 minutes and the flask was sealed and heated at 80°C for 24 h. The reaction mixture was cooled to room temperature and concentrated under a reduced pressure, and the residue was dissolved in dichloromethane and washed with brine. The organic layer was dried with anhydrous Na_2SO_4 and concentrated under a reduce pressure. The crude mixture was purified by column chromatography on silica gel with hexane/ethyl acetate (5:1, v/v) as eluent to give **8** (106 mg, 75%) as yellow solids. mp: 134-137 °C ^{1}H NMR (400 MHz, CD_2Cl_2) δ (ppm): 8.59 (d, J = 1.8 Hz, 2H), 7.78

(dd, J = 8.5, 1.8 Hz, 2H), 7.69-7.65 (m, 4H), 7.59-7.55 (m, 6H), 7.43 (td, J = 7.6, 1.5 Hz, 2H), 7.34 (td, J = 7.6, 1.4 Hz, 2H), 6.94 (dd, J = 8.3, 1.9 Hz, 2H), 6.70 (d, J = 1.9 Hz, 2H), 6.67 (d, J = 8.3 Hz, 2H), 3.73 (s, 6H), 3.41 (s, 6H), 1.45 (s, 9H). ^{13}C NMR (101 MHz, CD_2Cl_2) δ (ppm): 151.23, 149.98, 149.11, 144.43, 141.29, 135.10, 132.93, 132.92, 130.08, 128.77, 128.20, 127.30, 127.08, 126.67, 124.73, 123.46, 122.19, 121.58, 115.69, 114.25, 111.43, 109.78, 92.74, 88.68, 56.02, 55.62, 35.12, 31.54. HRMS (APCI): calcd. for $C_{54}H_{45}NO_4$ ($[M+H]^+$): 772.34214, found: 772.34055.

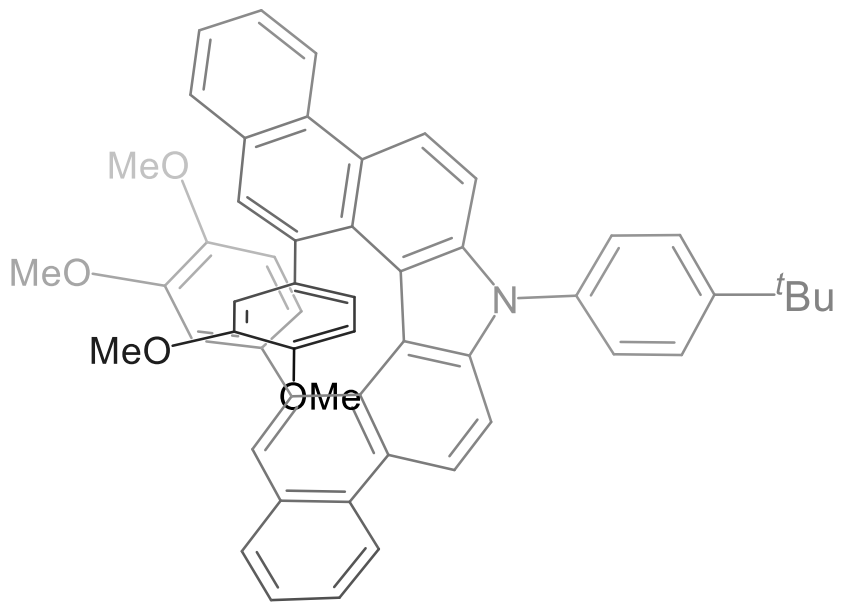

Compound 8

To a flask containing 15.4 mg (0.02 mmol) of **7** and 2.8 mg (0.012 mmol) of $InCl_3$ under a N_2 atmosphere was added 7 mL of toluene. The reaction mixture was heated under reflux for 24 hours. The reaction mixture was cooled to room temperature and concentrated under a reduced pressure. The crude product was purified by preparative thin-layer chromatography (silica gel) using hexane/ethyl acetate (4:1, v/v) as the eluent. The final fraction was collected to obtain compound **8** as a yellow solid (8.5 mg, 55%). mp: 151-153 °C ^{1}H NMR (400 MHz, CD_2Cl_2 + CS_2) δ (ppm): 8.58-8.55 (m, 4H), 7.87 (d, J = 7.8 Hz, 2H), 7.75 (d, J = 8.5 Hz, 2H), 7.70 (d, J = 8.8 Hz, 2H), 7.64-7.59 (m, 6H), 7.53 (t, J = 7.3 Hz, 2H), 7.47 (s, 2H), 6.27 (br, 2H), 5.70 (br, 2H), 2.96 (s, 6H), 2.72 (br, 6H), 1.50 (s, 9H). ^{13}C NMR (101 MHz, CD_2Cl_2 + CS_2) δ (ppm): 151.62, 147.65, 138.78, 137.90, 134.85, 134.04, 131.35, 129.78, 128.22, 127.59, 127.49, 127.45, 127.08, 126.52, 126.29, 125.58, 122.84, 122.61, 121.70, 120.05, 113.61, 111.34, 110.31, 35.17, 31.64, 30.22. HRMS (APCI): calcd. for $C_{54}H_{45}NO_4$ ($[M+H]^+$): 772.34214, found: 772.34138.

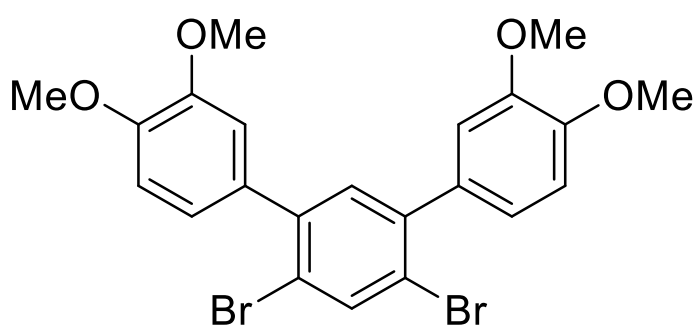

4',6'-dibromo-3,3'',4,4''-tetramethoxy-1,1':3',1''-terphenyl (10)

To a mixture of 1,5-dibromo-2,4-diiodobenzene [4] (2.00 g, 4.1 mmol), (3,4-methoxyphenyl)boronic acid (1.49 g, 8.2 mmol), $Pd(dppf)_2Cl_2$ (150 mg, 0.21 mmol) and K_2CO_3 (5.67 g, 41 mmol) under a N_2 atmosphere were added 40 ml of THF and 25 ml of water, both of which were bubbled with a flow of N_2 for 20 minutes prior to use. The reaction flask was then sealed and the reaction mixture was heated

at 70°C overnight. The reaction mixture was cooled to room temperature, concentrated under a reduced pressure. The residue was then diluted with dichloromethane, washed with brine and the organic layer was dried with anhydrous Na_2SO_4. After concentrated under a reduced pressure, the crude product was purified by flash column chromatography on silica gel using hexane/ethyl acetate (3/1, v/v) as eluent. The product was further triturated with methanol, filtered and dried in air to obtain compound **10** as a white solid (1.07 g, 51%). mp: 165-166 °C ^{1}H NMR (400 MHz, $CDCl_3$) δ (ppm): 7.97 (s, 1H), 7.32 (s, 1H), 6.97-6.92 (m, 6H), 3.93 (s, 6H), 3.90 (s, 6H). ^{13}C NMR (101 MHz, $CDCl_3$) δ (ppm): 148.95, 148.50, 141.64, 136.82, 133.42, 132.57, 121.79, 121.75, 112.77, 110.81, 56.12, 56.04. HRMS (APCI): calcd. for $C_{22}H_{20}Br_2O_4$ ($[M+H]^+$): 508.97821, found: 508.97806.

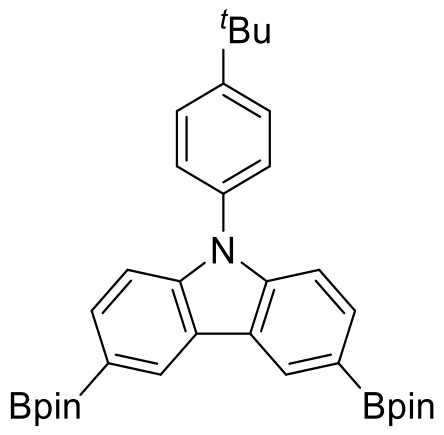

9-(4-*tert*-butylphenyl)-3,6-bis(4,4,5,5-tetramethyl-1,3,2-dioxaborolan-2- yl)-9*H*-carbazole (11)

Under a nitrogen atmosphere, 20 mL of dioxane was injected into a flask containing 3,6-dibromo-9-(4-tert-butylphenyl)-9H-carbazole[1] (1.0 g, 2.18 mmol), bis(pinacolato)diboron (1.67 g, 6.58 mmol), Pd(dppf)Cl_2 (321 mg, 0.44 mmol) and potassium acetate (1.28 g, 13.0 mmol). The resulting mixture was bubbled with a flow of N_2 for 20 minutes and stirred at 90 °C for 24 h. The mixture was cooled to room temperature and extracted with ethyl acetate, washed by water and brine, dried over anhydrous Na_2SO_4. The crude product was purified by column chromatography using hexane/CH_2Cl_2 (2/1 to 1/1, v/v) as eluent for step-gradient elution. The product was further triturated with methanol, filtered and dried in air to obtain 794 mg (1.44 mmol, 66%) of **11** as a white solid. m.p.: 348-350 ºC. ^{1}H-NMR (400 MHz, $CDCl_3$) δ (ppm): 8.72 (s, 2H), 7.85(dd, J = 8.2, 1.0 Hz, 2H), 7.62 (d, J = 8.6 Hz, 2H), 7.47 (d, J = 8.6 Hz, 2H), 7.39 (d, J = 8.2 Hz, 2H), 1.44 (s, 9H), 1.40 (s, 24H). ^{13}C NMR (101 MHz, $CDCl_3$) δ (ppm): 150.82, 143.35, 134.68, 132.30, 128.16, 126.93, 126.66, 123.27, 109.41, 34.95, 31.57, 25.08. HRMS (APCI): calcd. for $C_{34}H_{43}B_2NO_4$ ($[M+H]^+$): 552.34625, found: 552.34468.

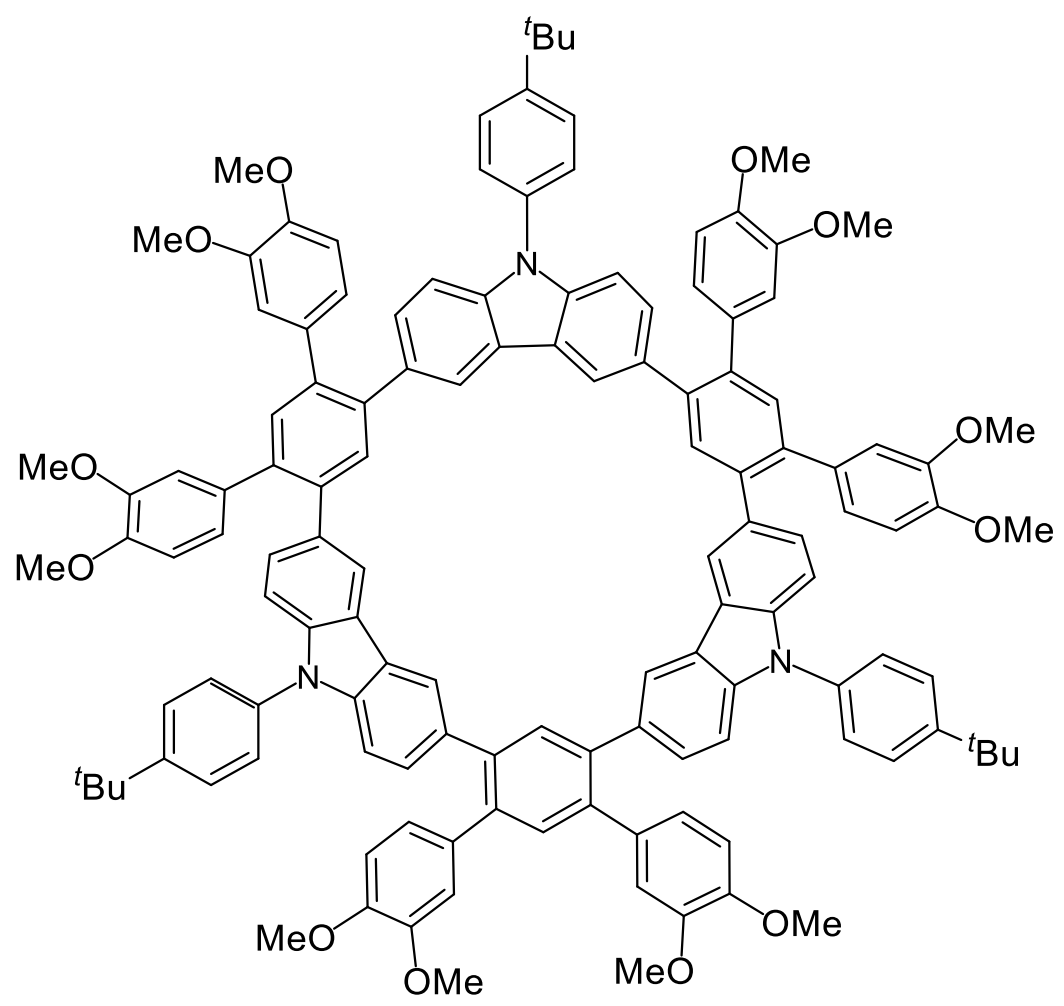

Compound 9

To a flask containing 795 mg (1.44 mmol) of **11**, 735 mg (1.45 mmol) of **10**, 6.0 g (43.5 mmol) of K_2CO_3 and 139 mg (0.43 mmol) of tetra-*n*-butylammonium bromide were added 650 mL of toluene and 38 mL of H_2O. The reaction mixture was then bubbled with a flow of nitrogen for 30 minutes prior to use. 198 mg (0.21 mmol) of $Pd_2(dba)_3$ and 178 mg (0.43 mmol) of SPhos were then added to the reaction mixture. The reaction mixture was further bubbled with nitrogen for 1 hour and the flask was sealed and heated at 80°C for 5 days. The reaction mixture was cooled to room temperature and concentrated under a reduced pressure, and the residue was dissolved in dichloromethane and washed with brine. The organic layer was dried with anhydrous Na_2SO_4 and concentrated under a reduce pressure. The crude mixture was purified by gel permeation chromatography on a preparative recycling HPLC system using $CHCl_3$ as eluent to give **9** (111 mg, 12%) as white solids. mp: >300 °C ^{1}H NMR (400 MHz, $CDCl_3$) δ (ppm): 8.07 (s, 6H), 7.62 (s, 3H), 7.57 (d, *J* = 8.6 Hz, 6H), 7.56 (s, 3H), 7.43 (d, *J* = 8.5 Hz, 6H), 7.23 (d, *J* = 8.5 Hz, 6H), 7.12 (d, *J* = 8.5 Hz, 6H), 6.90 (dd, *J* = 8.2, 1.8 Hz, 6H), 6.82 (d, *J* = 1.7 Hz, 6H), 6.77 (d, *J* = 1.7 Hz, 6H), 3.84 (s, 18H), 3.57 (s, 18H), 1.40 (s, 27H). ^{13}C NMR (101 MHz, $CDCl_3$) δ (ppm): 150.46, 148.28, 147.79, 140.12, 140.01, 138.84, 134.98, 134.87, 134.07, 133.57, 131.93, 128.14, 126.80, 126.31, 123.53, 122.29, 121.19, 113.57, 110.93, 109.28, 55.86, 55.78, 34.85, 31.50. HRMS (APCI): calcd. for $C_{132}H_{117}N_3O_{12}$ ($[M+H]^+$): 1937.87434, found: 1937.87855.

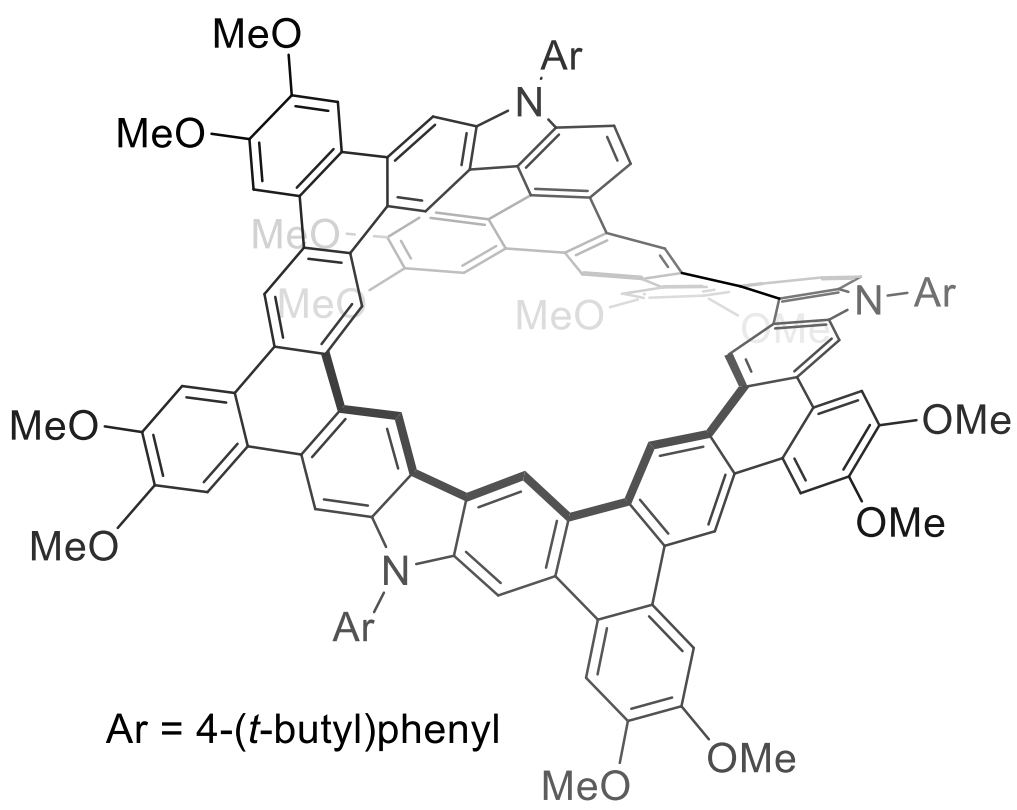

Compound 1

Compound **9** (16 mg, 8 μmol) was added to a reaction flask and the flask was purged with nitrogen three times. Anhydrous dichloromethane (20 mL) was then added to the reaction flask. $FeCl_3$ (36mg, 220 μmol) dissolved in 0.4 mL of nitromethane was quickly added to the above solution. After stirring for 20 minutes at room temperature, triethylamine was added to quench the reaction and the reaction mixture was poured into water. The organic layer was washed with brine and dried with anhydrous Na_2SO_4. After concentrated under a reduced pressure, the mixture was purified by column chromatography on silica gel with hexane/CH_2Cl_2/diethyl ether (1:5:1, v/v) as eluent to isolate pure compound **1** (12 mg, 75%). mp: >300 °C ^{1}H NMR (400 MHz, $CDCl_3$) δ (ppm): 10.08 (s, 1H), 9.99 (s, 1H), 9.66 (s, 1H), 9.28 (s, 1H), 9.17 (s, 1H), 9.10 (s, 1H), 8.98 (s, 1H), 8.93 (s, 1H), 8.80 (s, 1H), 8.70 (s, 1H), 8.53 (s, 1H), 8.48 (s, 1H), 8.33-8.31(m, 3H), 8.25(d, *J* = 8.8 Hz, 1H), 8.22 (s, 1H), 8.19 (s, 1H), 8.15 (s, 1H), 8.08 (s, 1H), 8.06 (s, 1H), 7.90 (s, 1H), 7.87 (s, 1H), 7.83 (s, 1H), 7.76-7.60 (m, 14H), 7.52-7.46(m, 4H), 4.24-4.22 (m, 12H), 4.12 (s, 3H), 4.10 (s, 3H), 4.08 (s, 3H), 4.07 (s, 3H), 4.00 (s, 3H), 3.94 (s, 6H), 3.61 (s, 3H), 1.51 (s, 9H), 1.49 (s, 9H), 1.45 (s, 9H). ^{13}C NMR (101 MHz, $CDCl_3$) δ (ppm): 151.39, 151.05, 150.16, 150.00, 149.88, 149.79, 149.69, 149.46, 149.46, 149.16, 149.08, 148.75, 143.88, 143.81, 143.42, 143.14, 142.05, 141.50, 135.31, 135.20, 135.01, 132.05, 131.78, 130.96, 130.69, 129.84, 129.48, 129.18, 128.82, 128.72, 128.49, 128.26, 127.98, 127.76, 127.75, 127.72, 127.40, 127.34, 127.22, 127.16,126.53, 126.50, 126.35, 126.11, 125.98, 125.62, 125.48, 125.38, 125.31, 125.17, 125.11, 125.06, 124.99, 124.94, 124.72, 124.523, 124.42, 124.40, 124.14, 123.75, 122.88, 122.78, 121.90, 120.75, 120.18, 118.90, 118.63, 118.51, 118.18, 117.73, 116.42, 115.84, 115.07, 114.39, 110.05, 109.98, 107.21, 107.00, 106.74, 106.60, 106.29, 105.99, 105.84 105.74, 105.68, 105.49, 105.26, 104.61, 102.59, 102.02, 56.66, 56.61, 56.56, 56.55, 56.48, 56.38, 56.33, 56.26, 56.21, 56.15, 35.10, 35.03, 31.64, 31.61, 31.58. HRMS (APCI): calcd. for $C_{132}H_{105}N_3O_{12}$ ($[M+H]^+$): 1925.78044, found: 1925.78173.

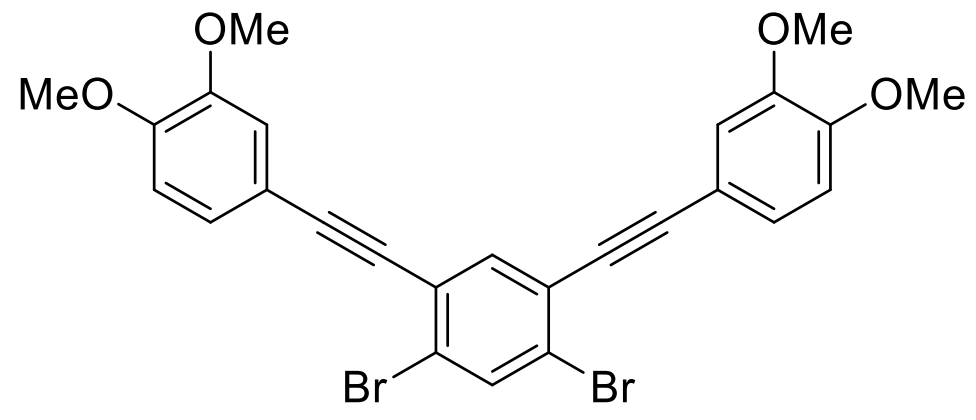

Compound 15a

To a mixture of 1,5-dibromo-2,4-diiodobenzene [4] (244 mg, 0.5 mmol), 4-ethynyl-1,2-dimethoxybenzene[5] (202 mg, 1.25 mmol), $Pd(PPh_3)_2Cl_2$ (3.5 mg, 0.0027 mmol) and CuI (1.9 mg, 0.01 mmol) under a N_2 atmosphere were added 8 ml of THF and 4 ml of Et_3N, both of which were bubbled with a flow of N_2 for 20 minutes prior to use. The reaction flask was then sealed and the reaction mixture was stirred at room temperature for 24 hours. The reaction mixture was concentrated under a reduced pressure. The crude product was purified by flash column chromatography on silica gel using hexane/ethyl acetate (5/1 to 3/1, v/v) as eluent. The collected product was further triturated with cold acetone, filtered and dried in air to obtain compound **15a** as a white solid (222 mg, 80%). mp: 169-171 °C ^{1}H NMR (400 MHz, $CDCl_3$) δ (ppm): 7.87 (s, 1H), 7.70 (s, 1H), 7.20-7.17 (dd, J = 8.3, 1.7 Hz, 2H), 7.04 (d, J = 1.6 Hz, 2H), 6.86 (d, J = 8.4 Hz, 2H), 3.92 (s, 12H). ^{13}C NMR (101 MHz, $CDCl_3$) δ (ppm): 150.23, 148.85, 136.42, 125.10, 124.82, 114.69, 114.29, 111.18, 95.66, 85.52, 56.08, 56.06. HRMS (APCI): calcd. for $C_{26}H_{21}Br_2O_4$ ($[M+H]^+$): 556.97826, found: 556.97775.

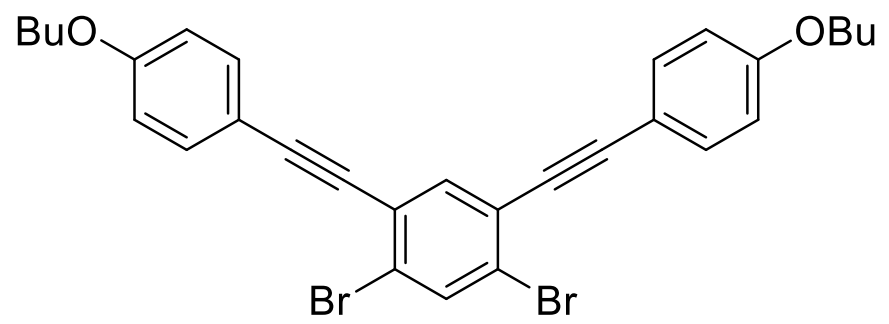

Compound 15b

To a mixture of 1,5-dibromo-2,4-diiodobenzene [4] (1.27 g, 2.61 mmol), 1-butoxy-4-ethynylbenzene[6] (1 g, 5.74 mmol), $Pd(PPh_3)_2Cl_2$ (182 mg, 0.26 mmol) and CuI (49.5 mg, 0.26 mmol) under a N_2 atmosphere were added 40 ml of THF and 20 ml of Et_3N, both of which were bubbled with a flow of N_2 for 30 minutes prior to use. The reaction flask was then sealed and the reaction mixture was stirred at room temperature for 24 hours. The reaction mixture was concentrated under a reduced pressure. The crude product was purified by flash column chromatography on silica gel using hexane/CH_2Cl_2 (6:1, v/v) as eluent. The collected product was further triturated with acetonitrile, filtered and dried in air to obtain compound **15b** as a white solid (754 mg, 50%). mp: 105-107 °C ^{1}H NMR (400 MHz, $CDCl_3$) δ (ppm): 7.85 (s, 1H), 7.67 (s, 1H), 7.49 (d, J = 8.8 Hz, 4H), 6.88 (d, J = 8.8 Hz, 4H), 3.99 (t, J = 6.5 Hz, 4H), 1.82-1.75 (m, 4H), 1.55-1.45 (m, 4H), 0.98 (t, J = 7.4 Hz, 6H). ^{13}C NMR (101 MHz, $CDCl_3$) δ (ppm): 159.93, 136.17, 135.70, 133.39, 125.23, 124.69, 114.76, 114.38, 95.70, 85.66, 67.94, 31.34, 19.36, 13.99. HRMS (APCI): calcd. for $C_{30}H_{28}Br_2O_2$ ($[M+H]^+$): 581.05108, found: 581.05151.

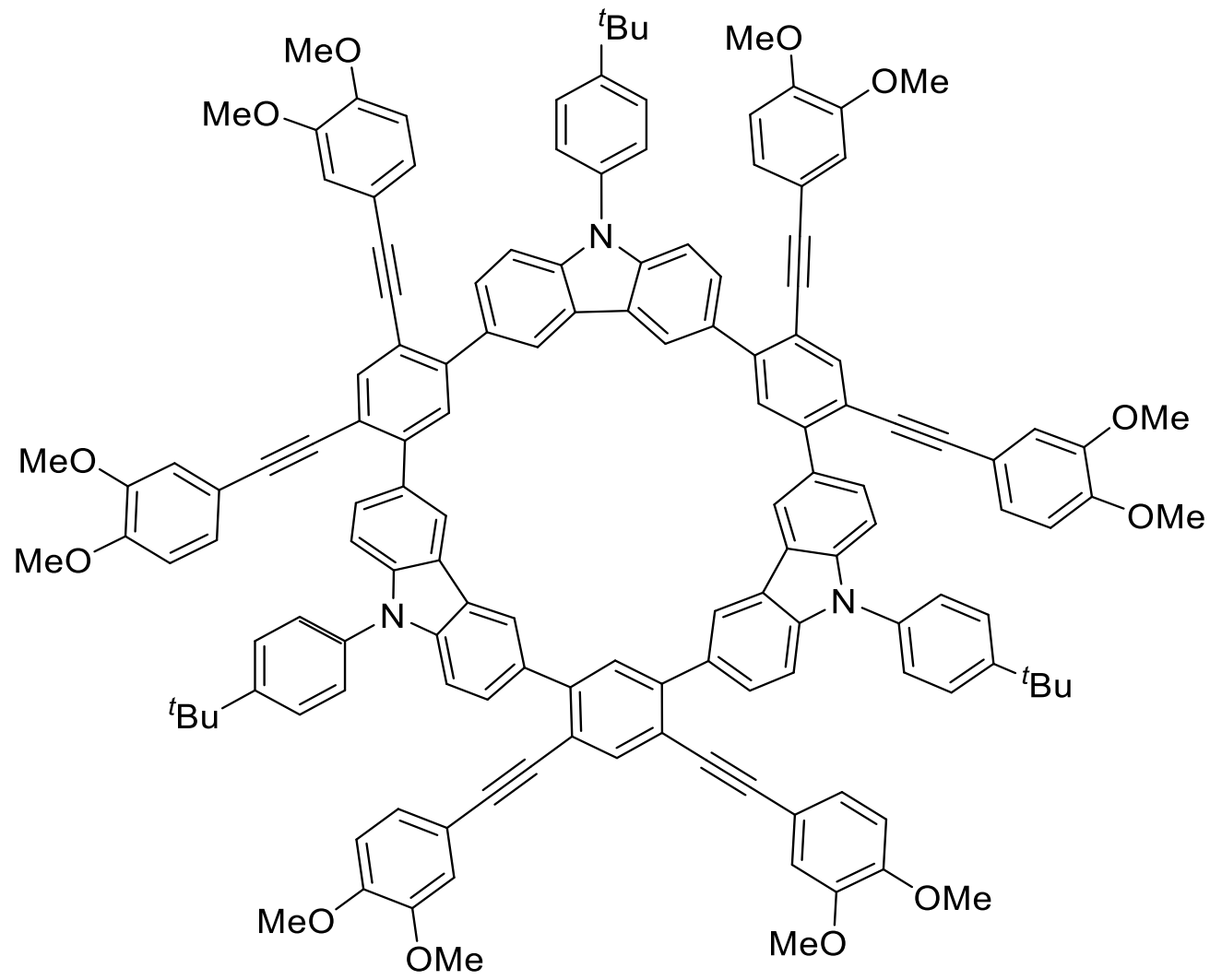

Compound 14a

To a flask containing 770 mg (1.4 mmol) of **11**, 777 mg (1.4 mmol) of **15a**, 5.8 g (42.05 mmol) of K_2CO_3 and 134 mg (0.41 mmol) of tetra-*n*-butylammonium bromide were added 650 mL of toluene and 38 mL of H_2O. The reaction mixture was then bubbled with a flow of nitrogen for 30 minutes. 192 mg (0.20 mmol) of $Pd_2(dba)_3$ and 172 mg (0.41 mmol) of SPhos were then added to the reaction mixture. The reaction mixture was further bubbled with nitrogen for 1 hour and the flask was sealed and heated at 80°C for 5 days. The reaction mixture was cooled to room temperature and concentrated under a reduced pressure, and the residue was dissolved in dichloromethane and washed with brine. The organic layer was dried with anhydrous Na_2SO_4 and concentrated under a reduce pressure. The crude mixture was first purified by column chromatography on silica gel with hexane/CH_2Cl_2/diethyl ether (2:4:1, v/v) as eluent to isolate the crude product. The crude product was further purified by gel permeation chromatography on a preparative recycling HPLC system using $CHCl_3$ as eluent to give **14a** (103 mg, 12%) as white solids. mp: >300 °C ^{1}H NMR (400 MHz, $CDCl_3$) δ (ppm): 8.30 (s, 6H), 7.93 (d, 6H, *J* = 8.52 Hz), 7.90 (s, 3H), 7.64(s, 3H), 7.62 (s, 6H), 7.55-7.50 (m, 12H), 6.95 (d, 6H, *J* = 8.24 Hz), 6.78 (s, 6H), 6.74 (d, 6H, *J* = 8.40 Hz), 3.83 (s, 18H), 3.62 (s, 18H), 1.43 (s, 27H). ^{13}C NMR (101 MHz, $CDCl_3$) δ (ppm): 150.81, 149.44, 148.59, 144.19, 141.01, 135.99, 135.00, 132.74, 132.29, 127.67, 126.96, 126.45, 124.57, 123.64, 121.22, 120.47, 115.77, 114.23, 111.04, 109.16, 93.07, 87.75, 55.97, 55.69, 34.95, 31.55. HRMS (MALDI-TOF): calcd. for $C_{144}H_{117}N_3O_{12}$ ($[M]^+$): 2080.8665, found: 2080.8663.

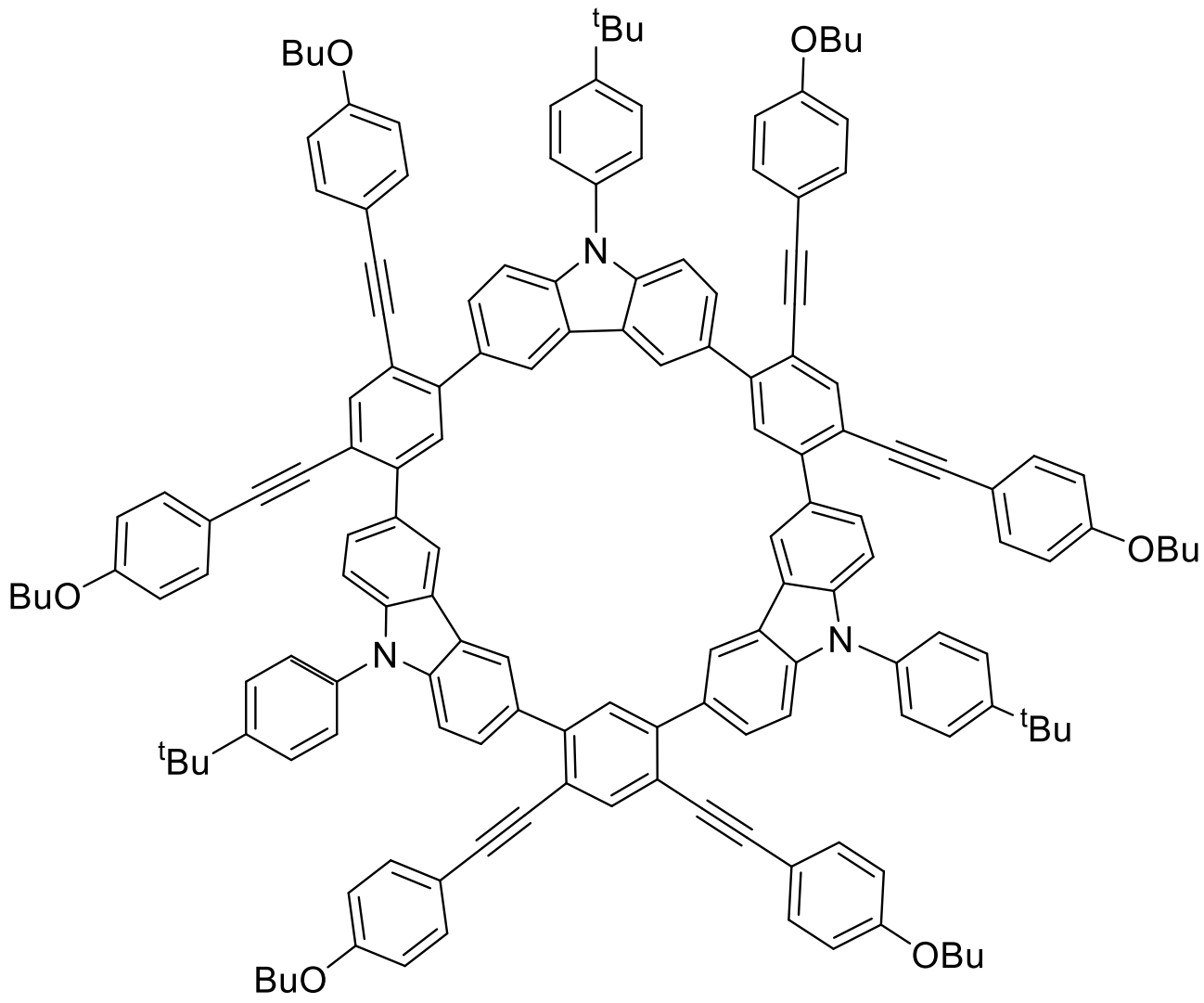

Compound 14b

To a flask containing 300 mg (0.55 mmol) of **11**, 315 mg (0.55 mmol) of **15b**, 2.26 g (16.35 mmol) of K_2CO_3 and 52 mg (0.16 mmol) of tetra-*n*-butylammonium bromide were added 250 mL of toluene and 15 mL of H_2O. The reaction mixture was then bubbled with a flow of nitrogen for 30 minutes prior to use. 74.8 mg (0.78 mmol) of $Pd_2(dba)_3$ and 67 mg (0.16 mmol) of SPhos were then added to the reaction mixture. The reaction mixture was further bubbled with nitrogen for 1 hour and the flask was sealed and heated at 80°C for 5 days. The reaction mixture was cooled to room temperature and concentrated under a reduced pressure, and the residue was dissolved in dichloromethane and washed with brine. The organic layer was dried with anhydrous Na_2SO_4 and concentrated under a reduce pressure. The crude mixture was first purified by column chromatography on silica gel with hexane/CH_2Cl_2 (1:1, v/v) as eluent to isolate the crude product. The crude product was further purified by gel permeation chromatography on a preparative recycling HPLC system using $CHCl_3$ as eluent to give **14b** (66 mg, 17%) as white solids. mp: 287-290 °C ^{1}H NMR (400 MHz, CD_2Cl_2 + CS_2) δ (ppm): 8.24 (d, J = 1.7Hz, 6H), 7.91 (dd, J = 8.6, 1.8 Hz, 6H), 7.81 (s, 3H), 7.67 (d, J = 8.9Hz, 6H), 7.60 (d, J = 8.4 Hz, 9H), 7.50 (d, J = 8.5 Hz, 6H), 7.23 (d, J = 8.9 Hz, 12H), 6.75 (d, J = 8.9 Hz, 12H), 3.93 (t, J = 6.5 Hz, 12H), 1.79-1.72 (m, 12H), 1.54-1.45 (m, 12H), 1.50 (s, 27H), 1.00 (t, J = 7.4 Hz, 18H). ^{13}C NMR (101 MHz, CD_2Cl_2 + CS_2) δ (ppm): 159.61, 150.87, 144.16, 141.27, 136.89, 135.28, 133.23, 132.97, 132.70, 127.96, 127.34, 126.75, 123.94, 121.35, 120.94, 115.57, 114.84, 109.54, 93.36, 88.12, 68.11, 35.04, 31.80, 31.68, 19.85, 14.25. HRMS (APCI): calcd. for $C_{156}H_{141}N_3O_6$ ($[M+H]^+$): 2154.09266, found: 2154.09325.

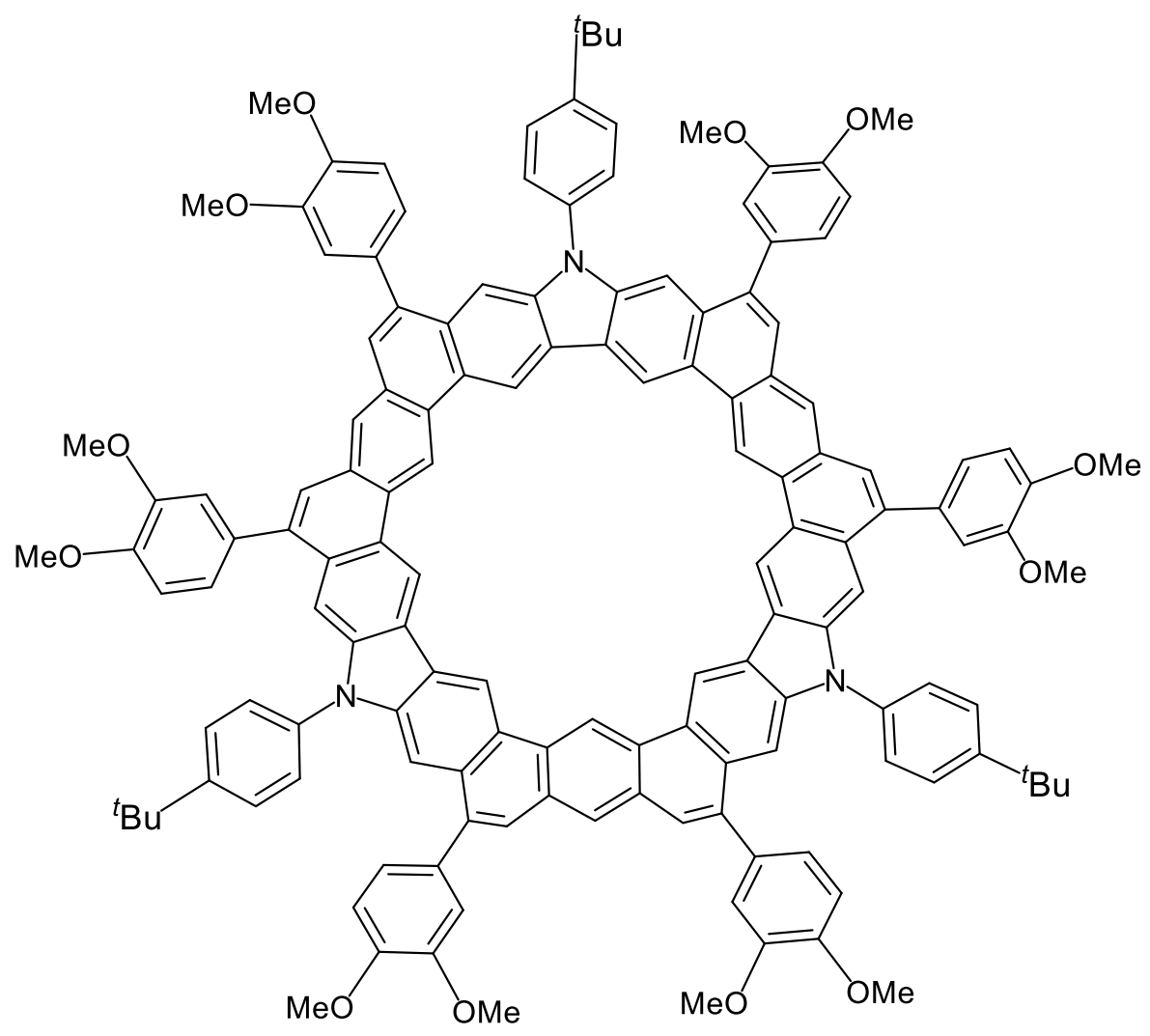

Compound 2a

To a flask containing 50 mg (0.024 mmol) of **14a** and 10 mg (0.045 mmol) of $InCl_3$ under a N_2 atmosphere were added 25 mL of toluene. The reaction mixture was heated under reflux for 24 hours. The reaction mixture was cooled to room temperature and concentrated under a reduced pressure. The crude product was purified by preparative thin-layer chromatography (silica gel) using hexane/CH_2Cl_2/diethyl ether (2:4:1, v/v) as the eluent. The final fraction was collected to obtain compound **2a** as a yellow solid (5 mg, 10%). mp: >300 °C ^{1}H NMR (400 MHz, CD_2Cl_2 + CS_2) δ (ppm): 10.06 (s, 3H), 9.98 (s, 6H), 8.30 (s, 3H), 7.88 (s, 6H), 7.74 (s, 6H), 7.51 (d, J = 8.8 Hz, 6H), 7.41 (d, J = 8.6 Hz, 6H), 7.17 (dd, J = 8.1, 2.0 Hz, 6H), 7.13 (d, J = 2.0 Hz, 6H), 6.95 (d, J = 8.4 Hz, 6H), 3.88 (s, 18H), 3.81 (s, 18H), 1.40 (s, 27H). ^{13}C NMR (101 MHz, CD_2Cl_2 + CS_2) δ (ppm): 150.22, 149.45, 149.23, 143.17, 138.37, 134.99, 133.78, 132.18, 131.37, 129.89, 128.25, 127.20, 127.04, 124.82, 122.67, 117.21, 114.00, 111.97, 107.07, 56.18, 56.15, 34.96, 31.55. HRMS (APCI): calcd. for $C_{144}H_{117}N_3O_{12}$ ($[M+H]^+$): 2081.87434, found: 2081.87685.

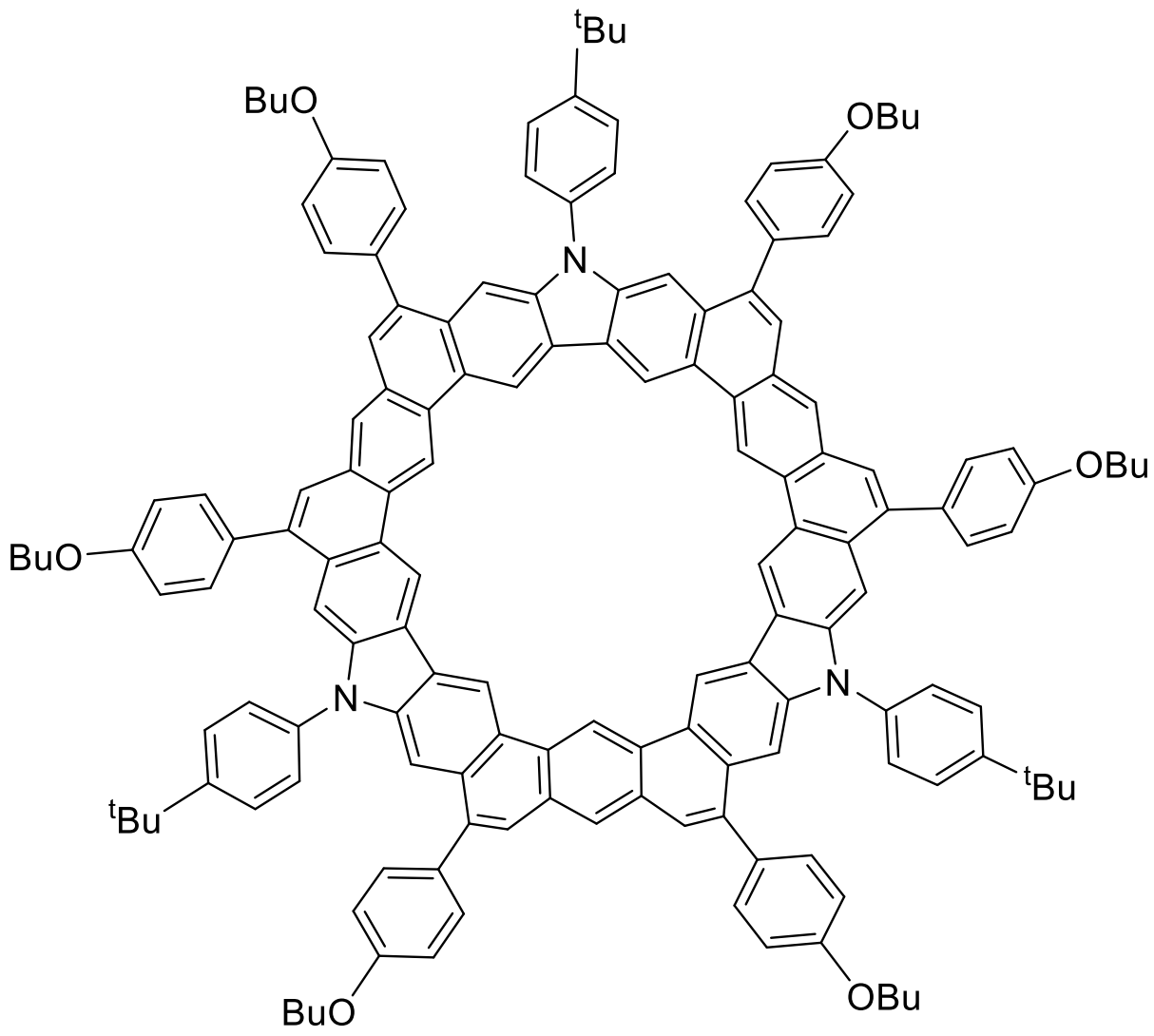

Compound 2b

To a flask containing 43 mg (0.02 mmol) of **14b** and 8.4mg (0.038 mmol) of $InCl_3$ under a N_2 atmosphere were added 20 mL of toluene. The reaction mixture was heated under reflux for 24 hours. The reaction mixture was cooled to room temperature and concentrated under a reduced pressure. The crude product was purified by preparative thin-layer chromatography (silica gel) using hexane/CH_2Cl_2 (1:1, v/v) as the eluent. The final fraction was collected to obtain compound **2b** as a yellow solid (7.8 mg, 18%). mp: >300 °C ^{1}H NMR (400 MHz, CD_2Cl_2 + CS_2) δ (ppm): 9.96 (s, 3H), 9.91 (s, 3H), 8.18 (s, 3H), 7.82 (s, 6H), 7.66 (s, 6H), 7.51 (d, J = 8.6 Hz, 12H), 7.47 (d, J = 8.6 Hz, 6H), 7.39 (d, J = 8.6 Hz, 6H), 6.95 (d, J = 8.8 Hz, 12H), 4.00 (t, J = 6.4 Hz, 12H), 1.85-1.78 (m, 12H), 1.60-1.50 (m, 12H), 1.40 (s, 27H), 1.05 (t, J = 7.4 Hz, 12H). ^{13}C NMR (101 MHz, CD_2Cl_2 + CS_2) δ (ppm): 159.06, 150.61, 142.91, 138.19, 135.06, 133.26, 132.27, 131.38, 131.29, 129.90, 129.17, 128.77, 128.22, 127.15, 127.08, 126.93, 124.86, 117.30, 117.19, 114.73, 107.05, 68.05, 34.86, 32.15, 31.80, 31.66, 30.54, 20.19, 14.63. HRMS (APCI): calcd. for $C_{156}H_{141}N_3O_6$ ($[M+H]^+$): 2154.09266, found: 2154.09569.

2. NMR Spectra

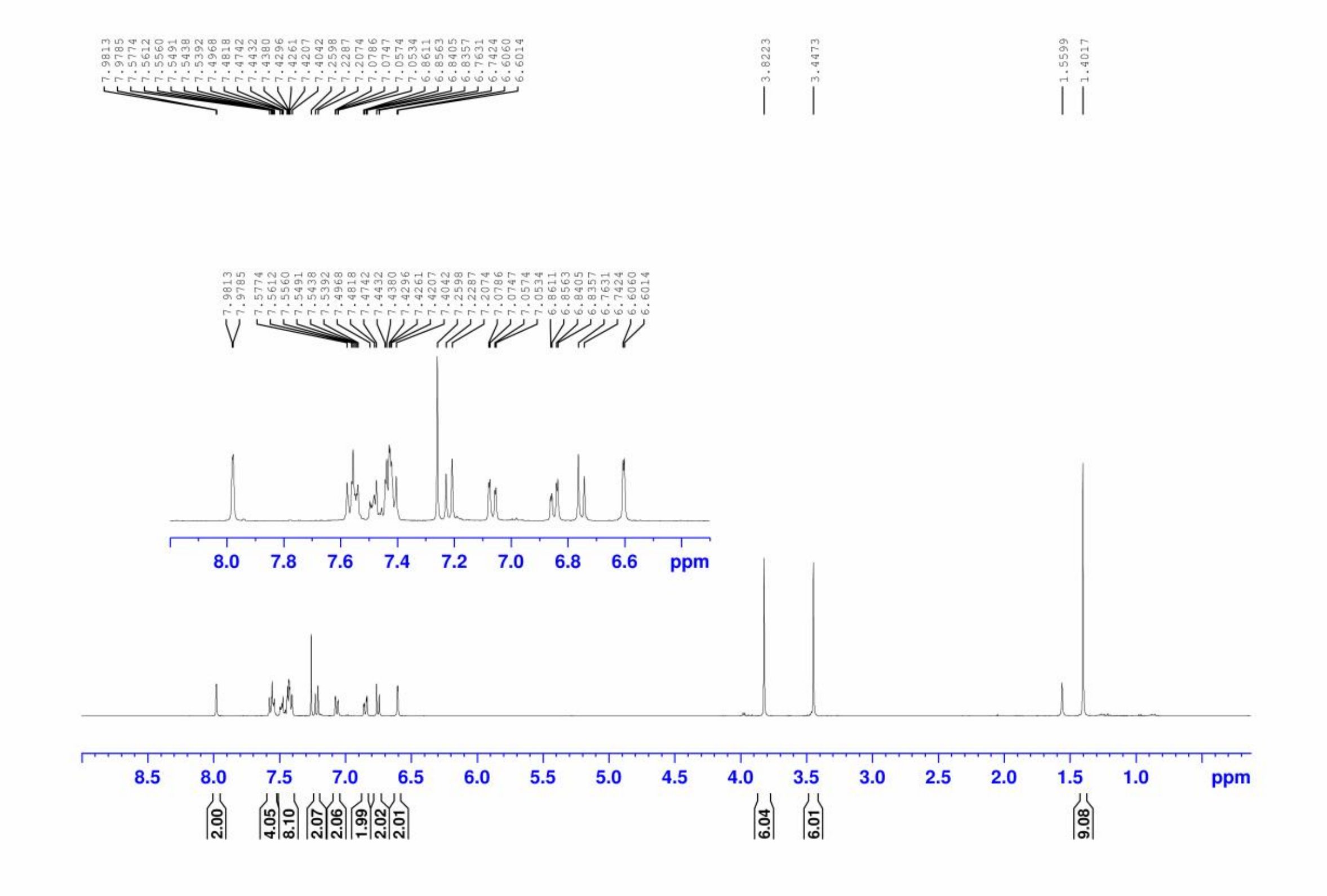

Figure S1. ^{1}H NMR spectrum (400MHz, $CDCl_3$) of **4**.

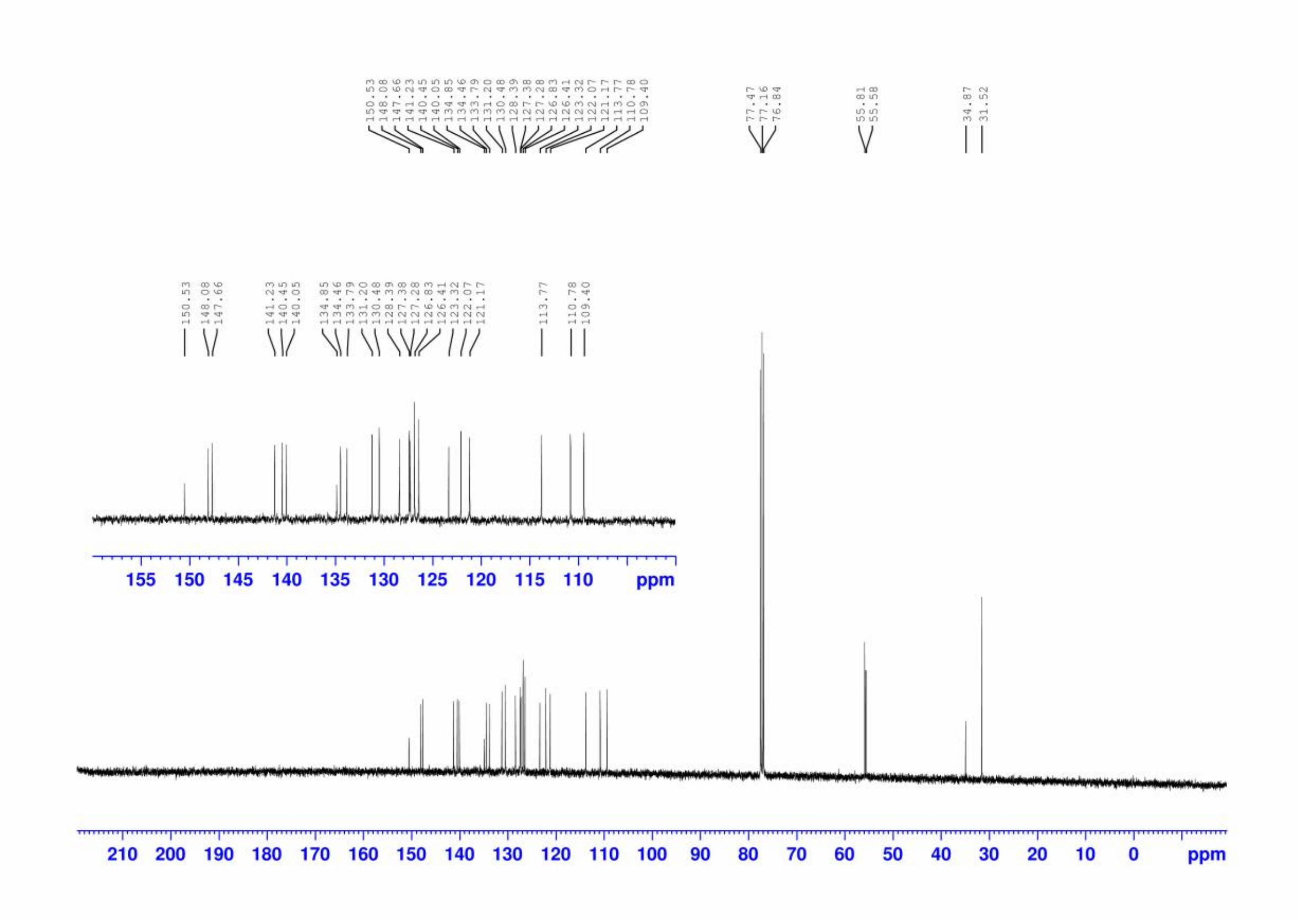

Figure S2. ^{13}C NMR spectrum (101MHz, $CDCl_3$) of **4**.

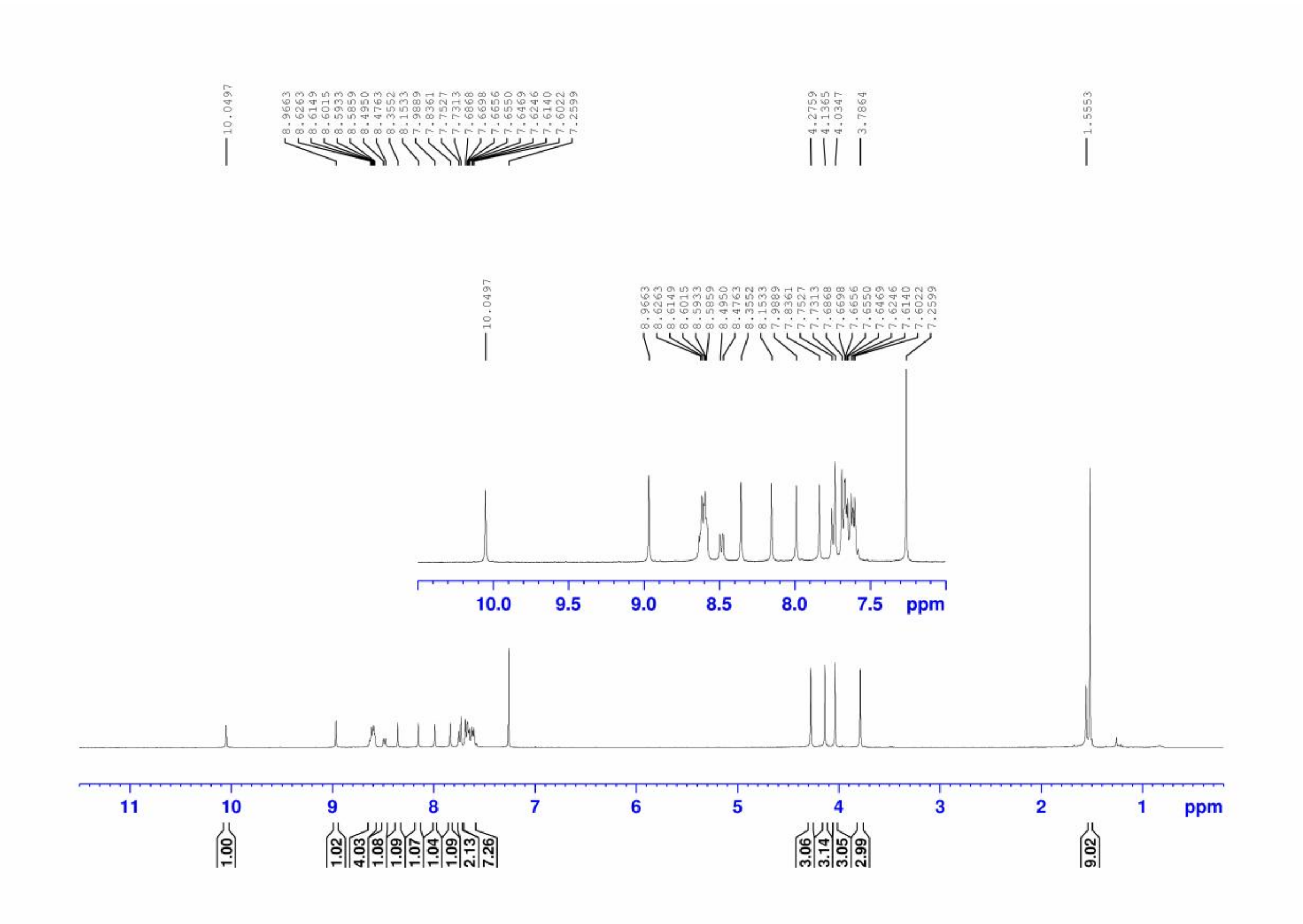

Figure S3. ^{1}H NMR spectrum (400MHz, $CDCl_3$) of **5**.

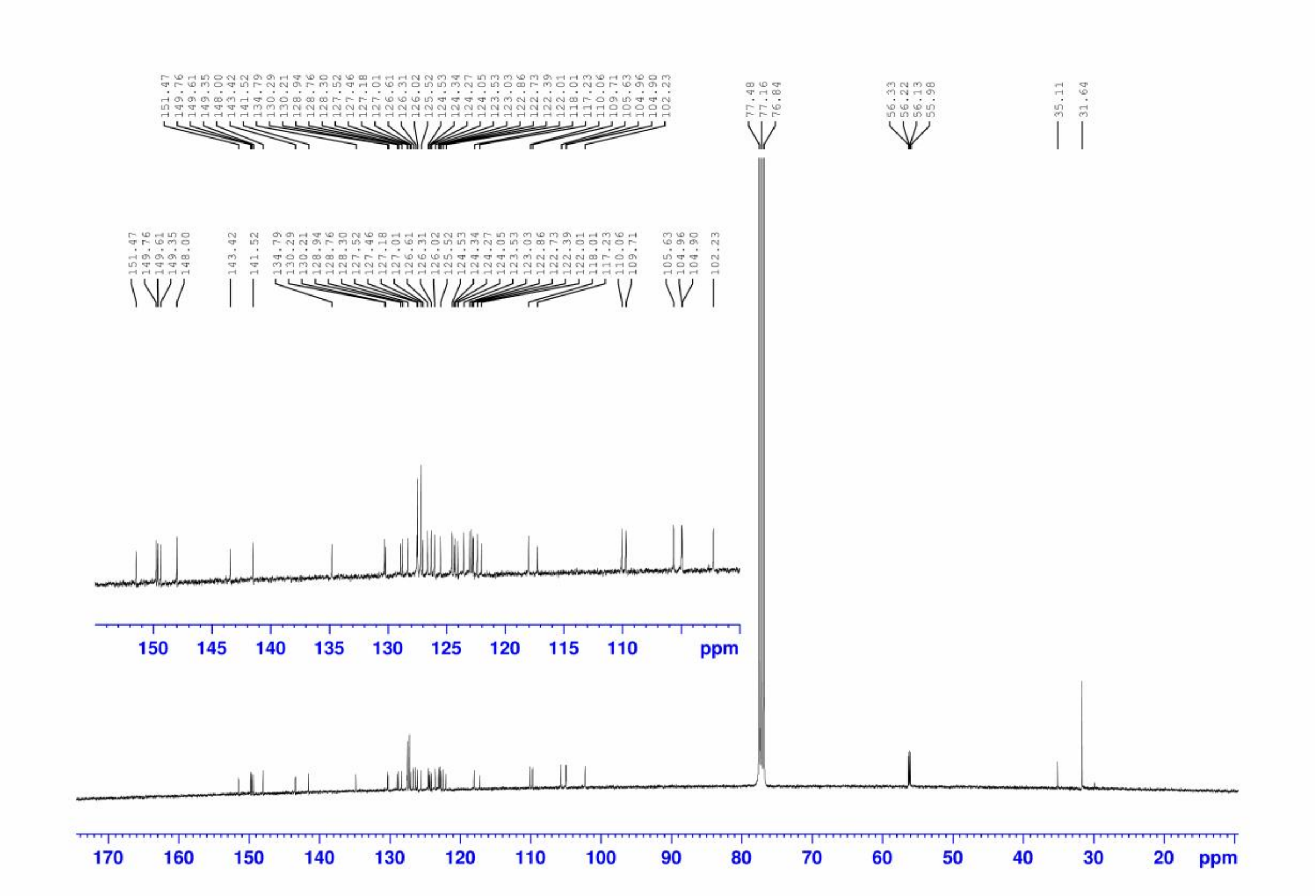

Figure S4. ^{13}C NMR spectrum (101MHz, $CDCl_3$) of **5**.

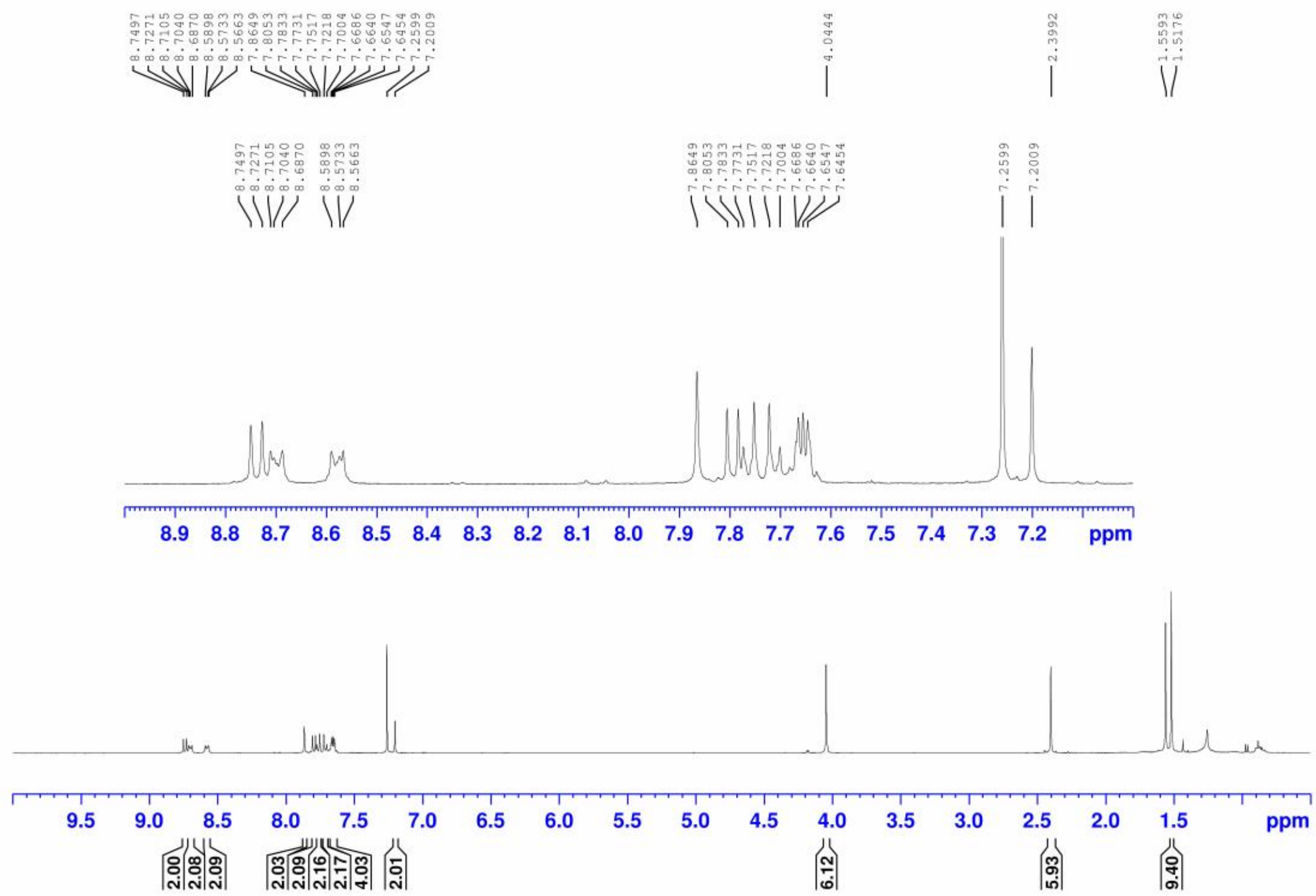

Figure S5. ^{1}H NMR spectrum (400MHz, $CDCl_3$) of **6**.

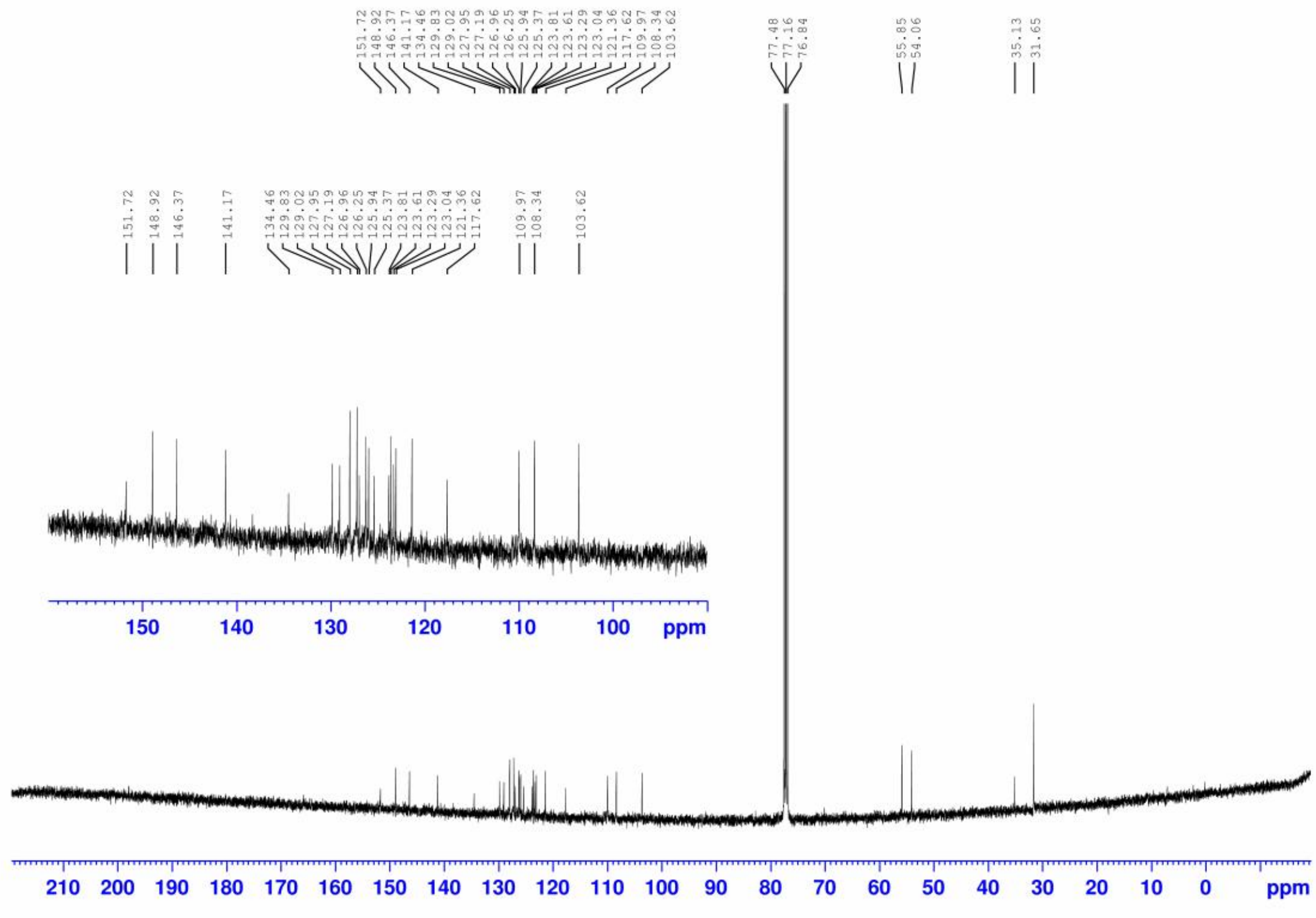

Figure S6. ^{13}C NMR spectrum (101MHz, $CDCl_3$) of **6**.

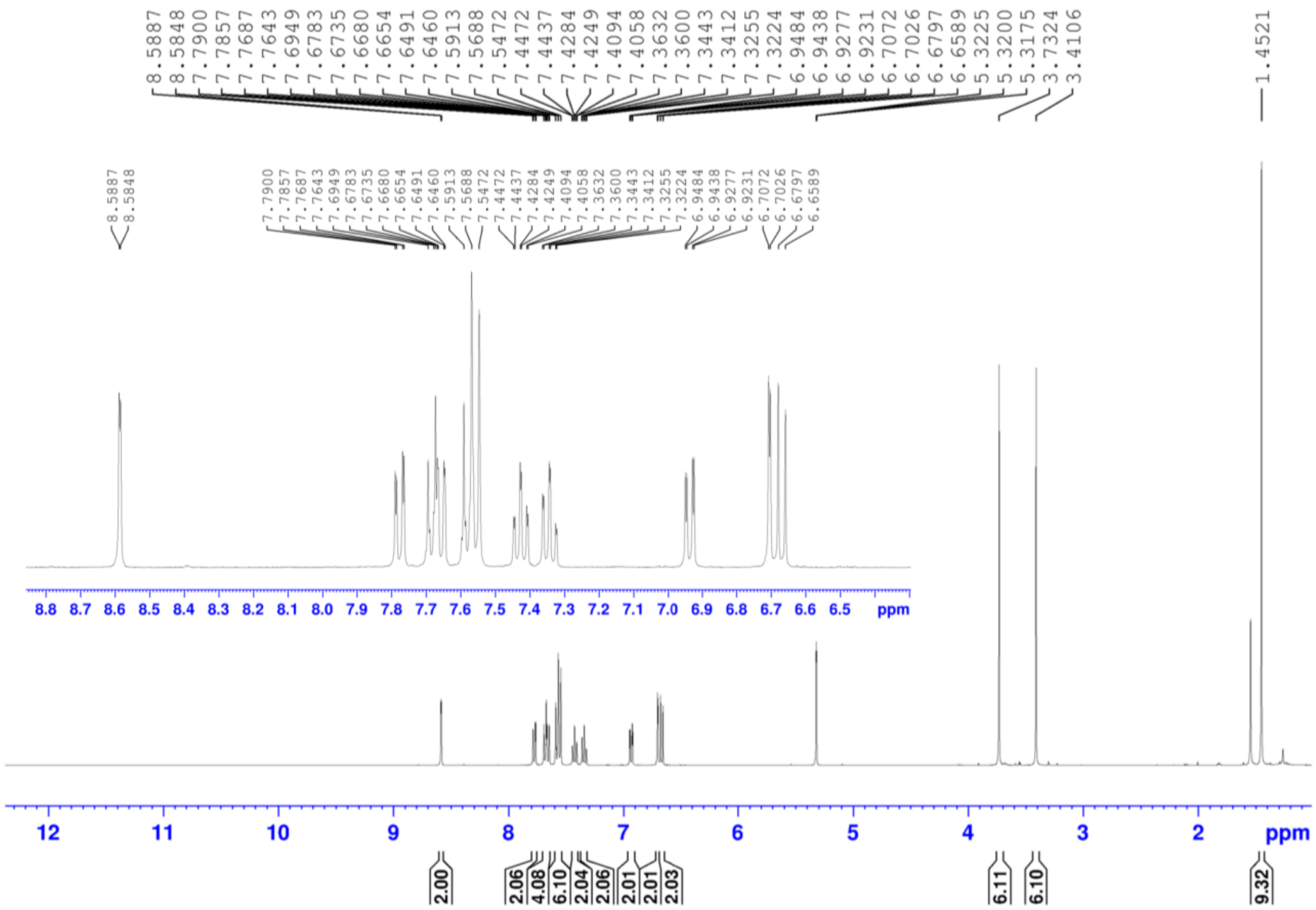

Figure S7. ^{1}H NMR spectrum (400MHz, CD_2Cl_2) of **7**.

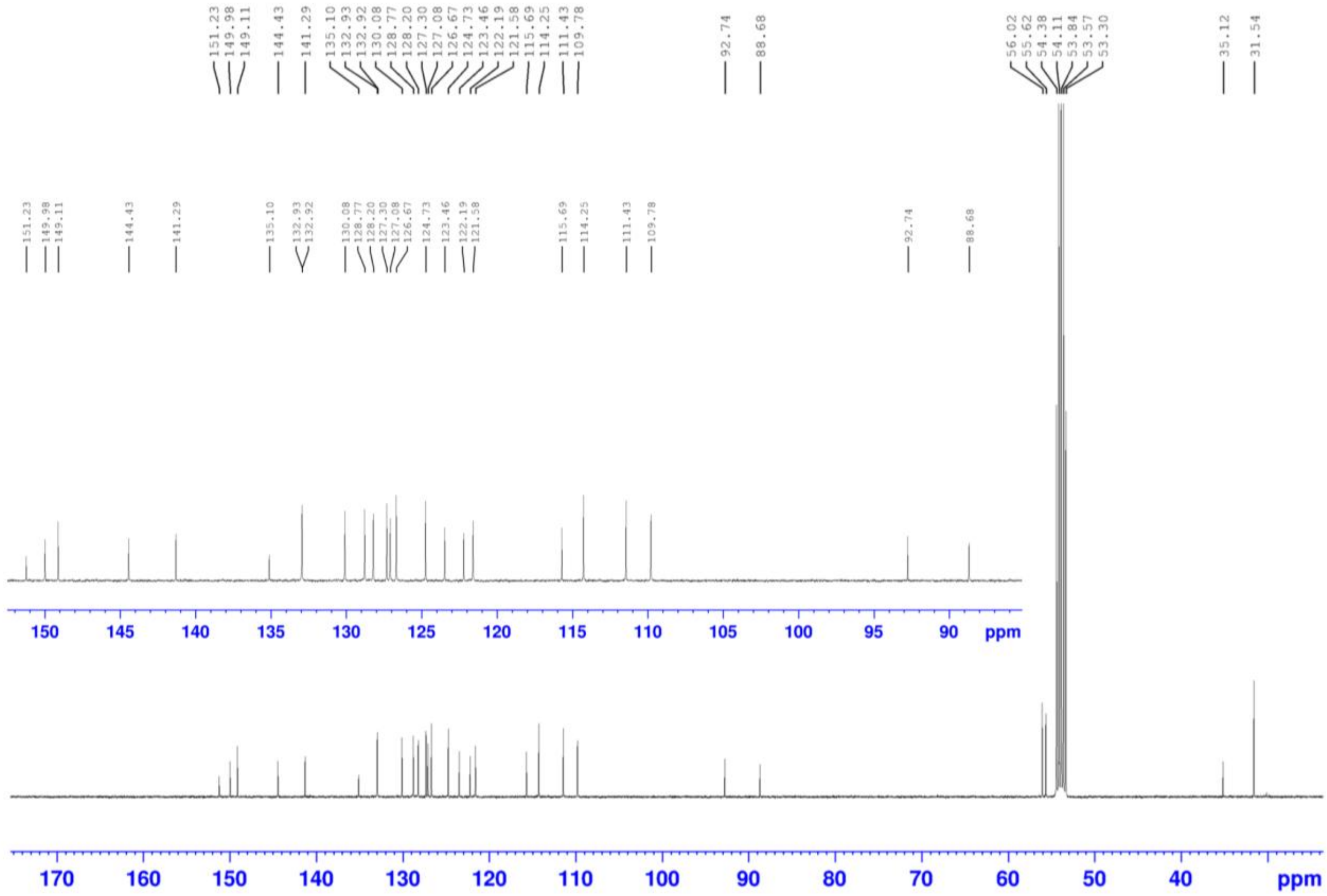

Figure S8. ^{13}C NMR spectrum (101MHz, CD_2Cl_2) of **7**.

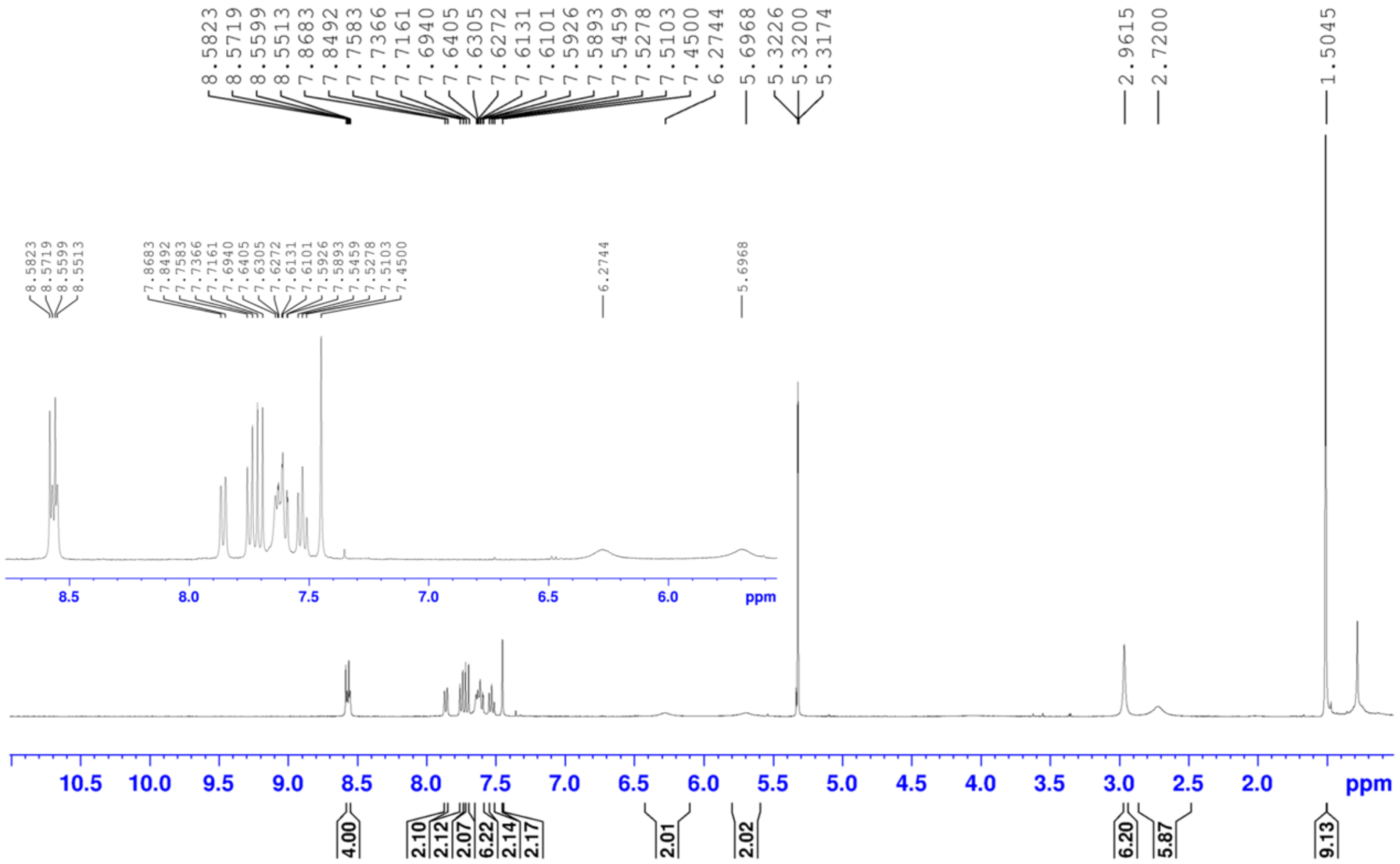

Figure S9. ^{1}H NMR spectrum (400MHz, CD_2Cl_2 + CS_2) of **8**.

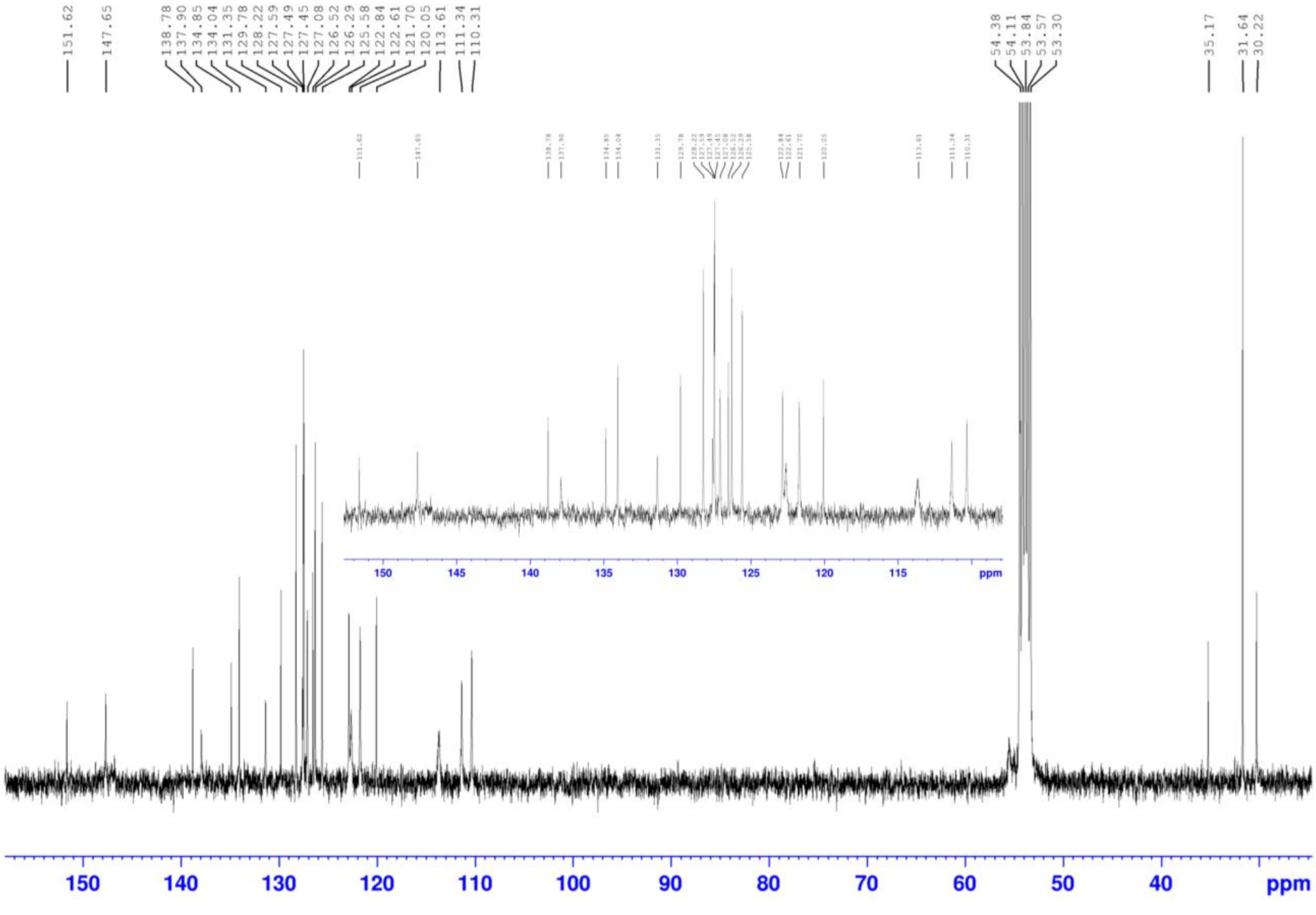

Figure S10. ^{13}C NMR spectrum (101MHz, CD_2Cl_2 + CS_2) of **8**.

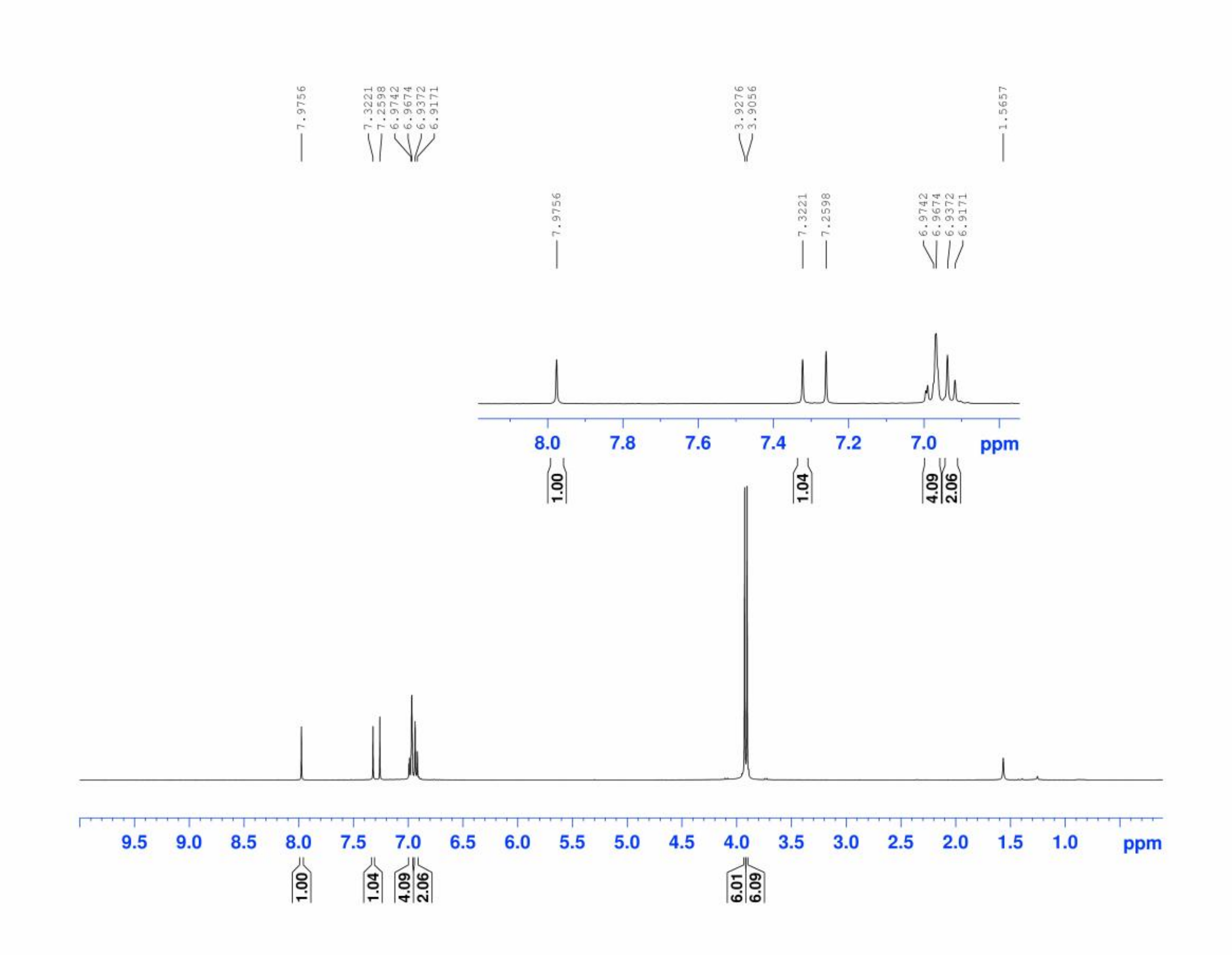

Figure S11. ^{1}H NMR spectrum (400MHz, $CDCl_3$) of **10**.

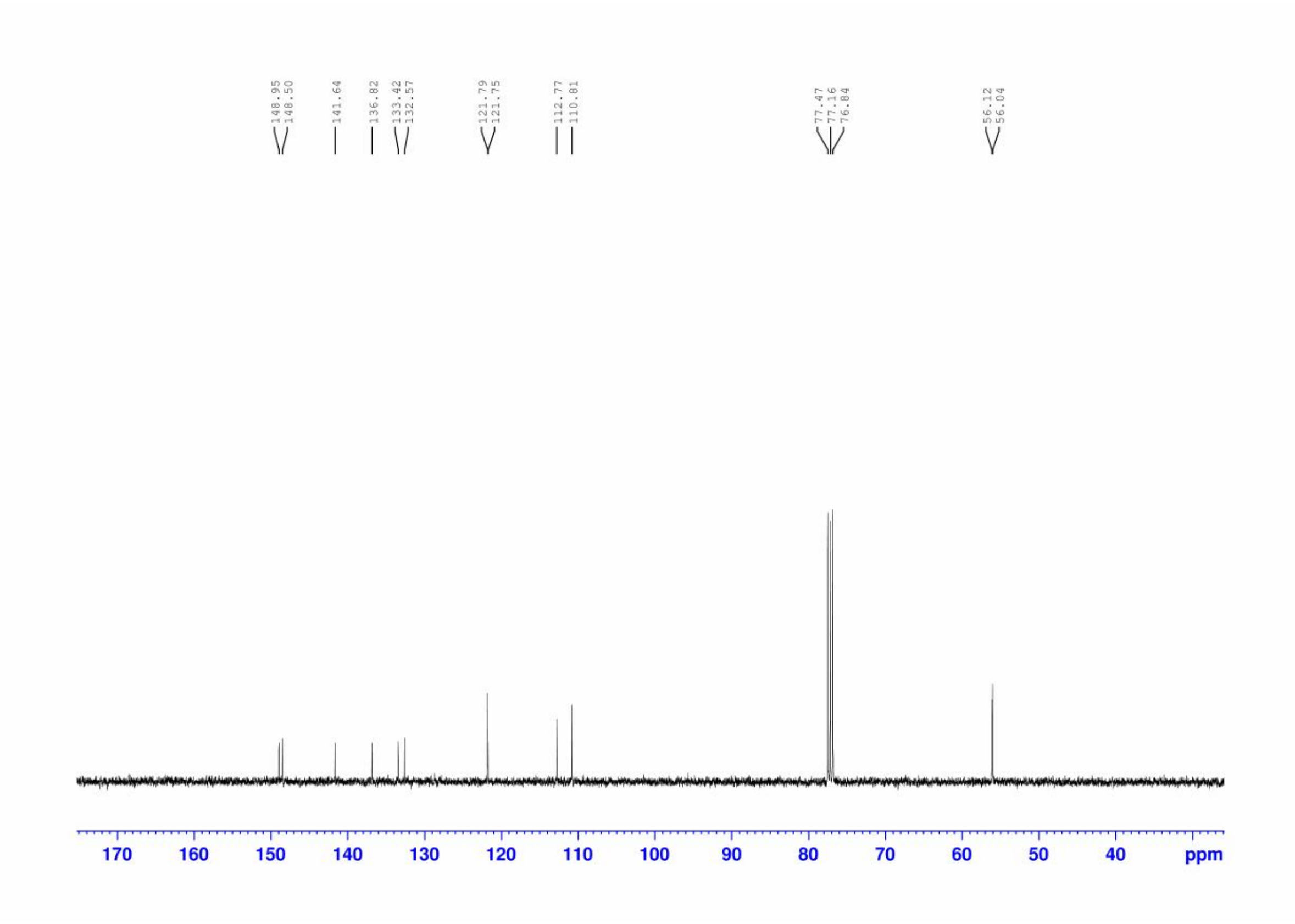

Figure S12. ^{13}C NMR spectrum (101MHz, $CDCl_3$) of **10**.

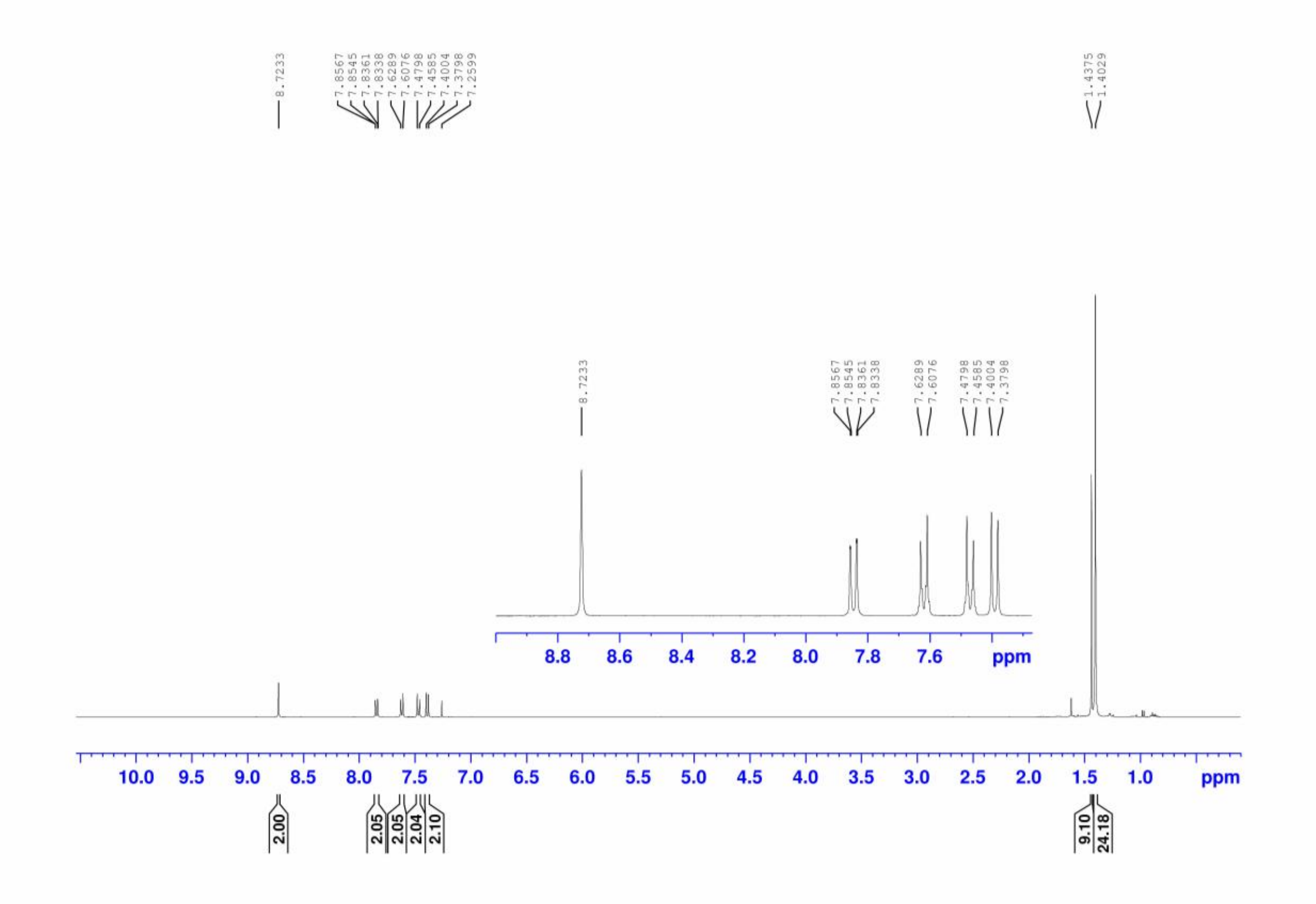

Figure S13. ^{1}H NMR spectrum (400MHz, $CDCl_3$) of **11**.

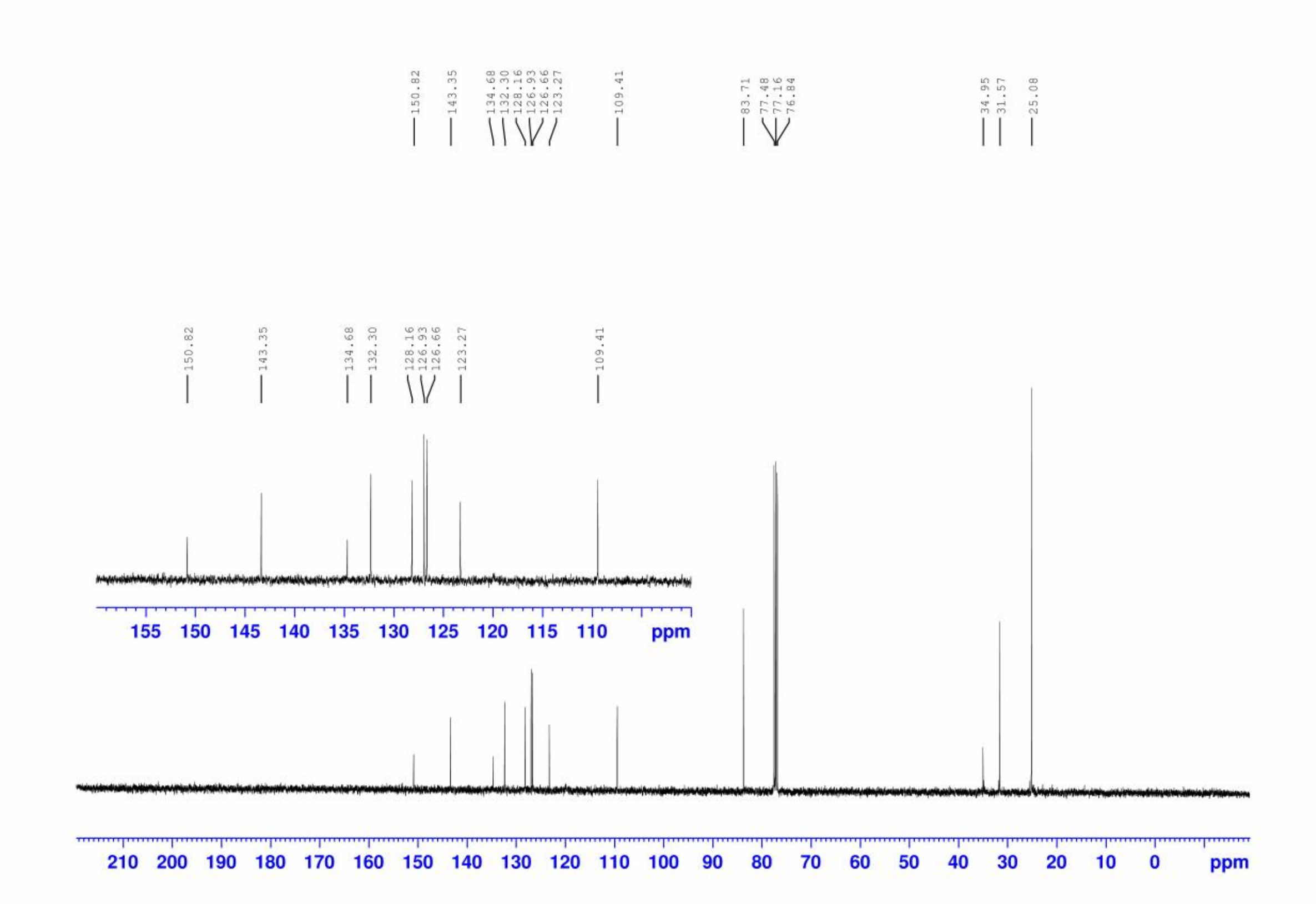

Figure S14. ^{13}C NMR spectrum (101MHz, $CDCl_3$) of **11**.

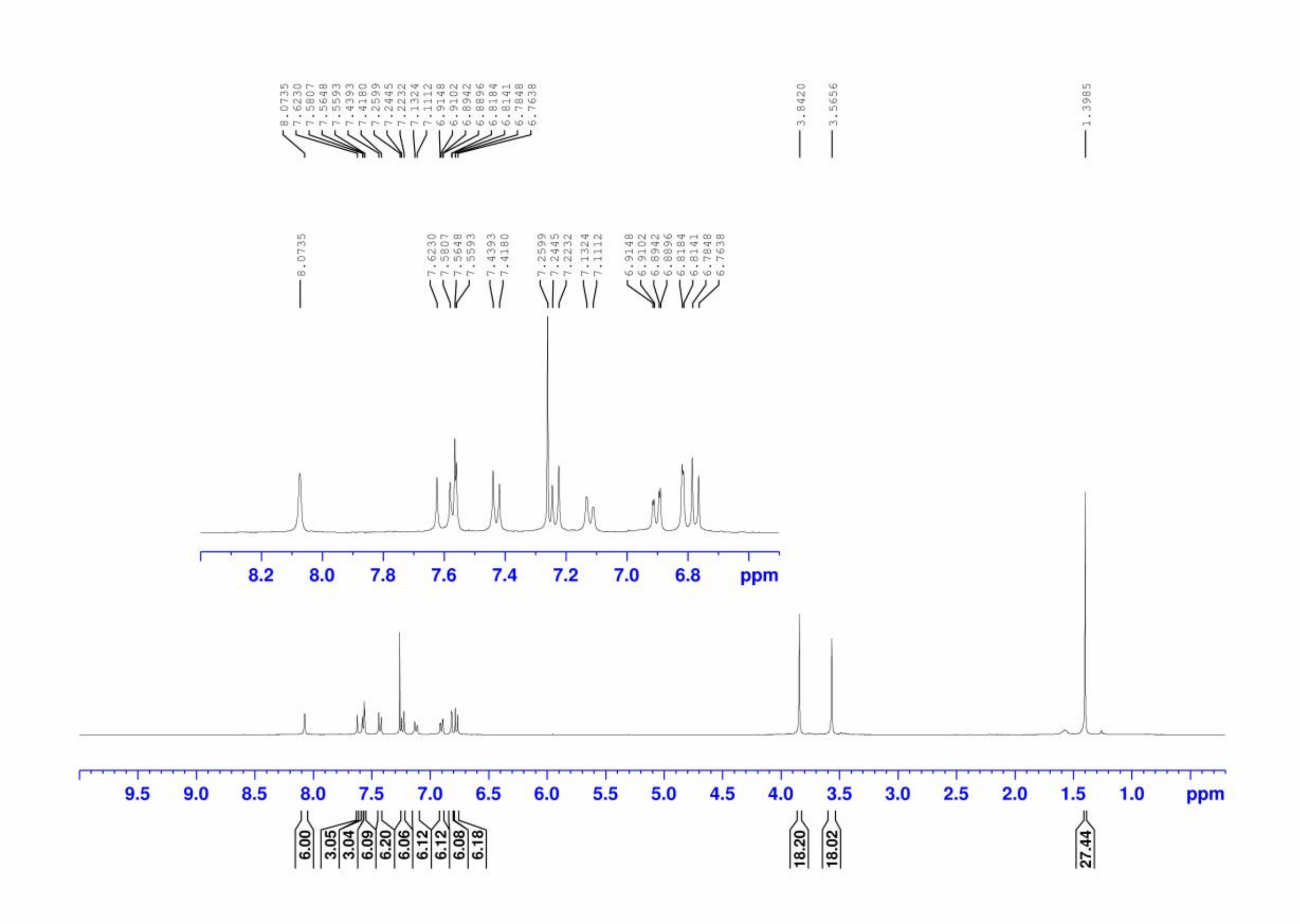

Figure S15. ^{1}H NMR spectrum (400MHz, $CDCl_3$) of **9**.

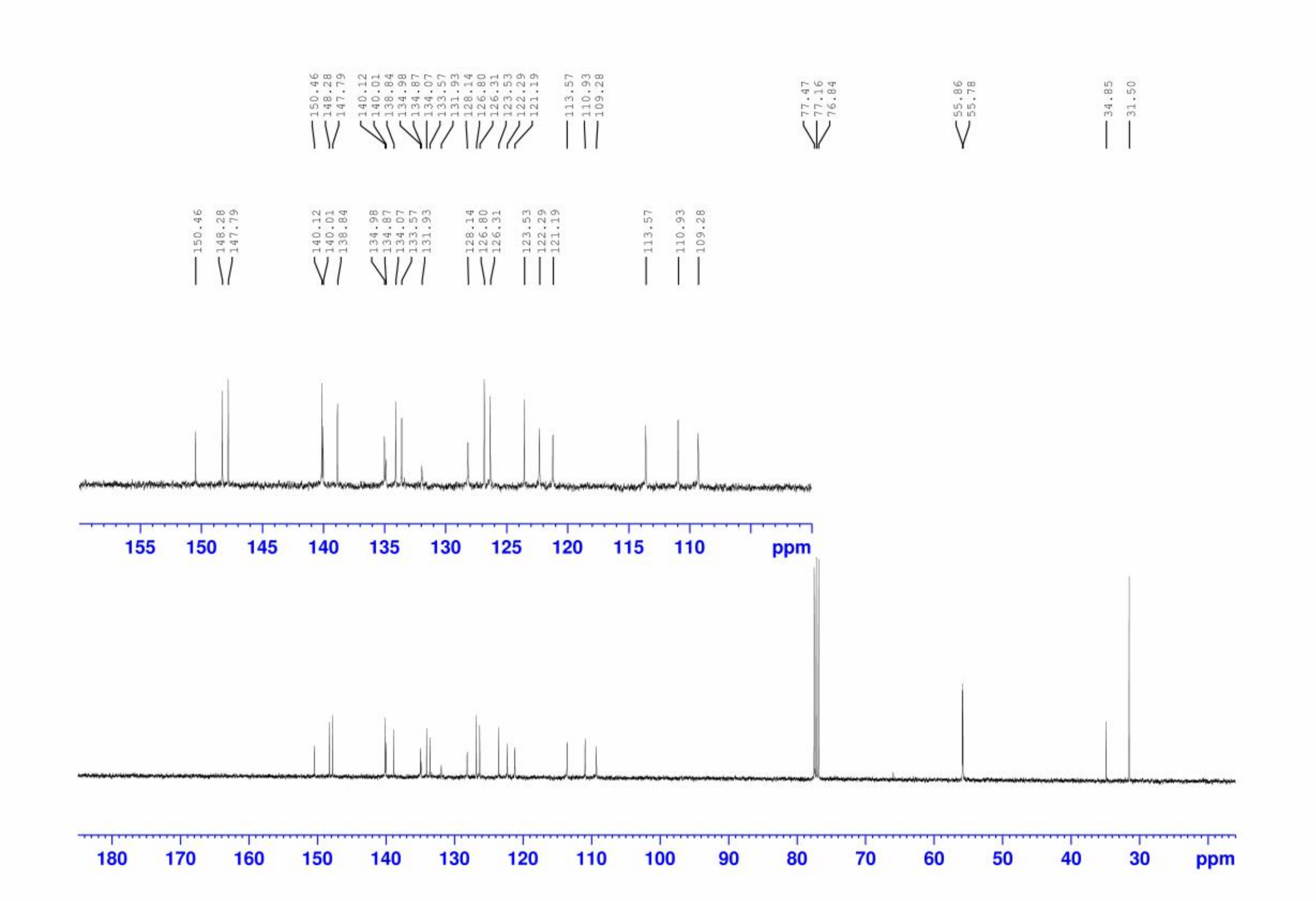

Figure S16. ^{13}C NMR spectrum (101MHz, $CDCl_3$) of **9**.

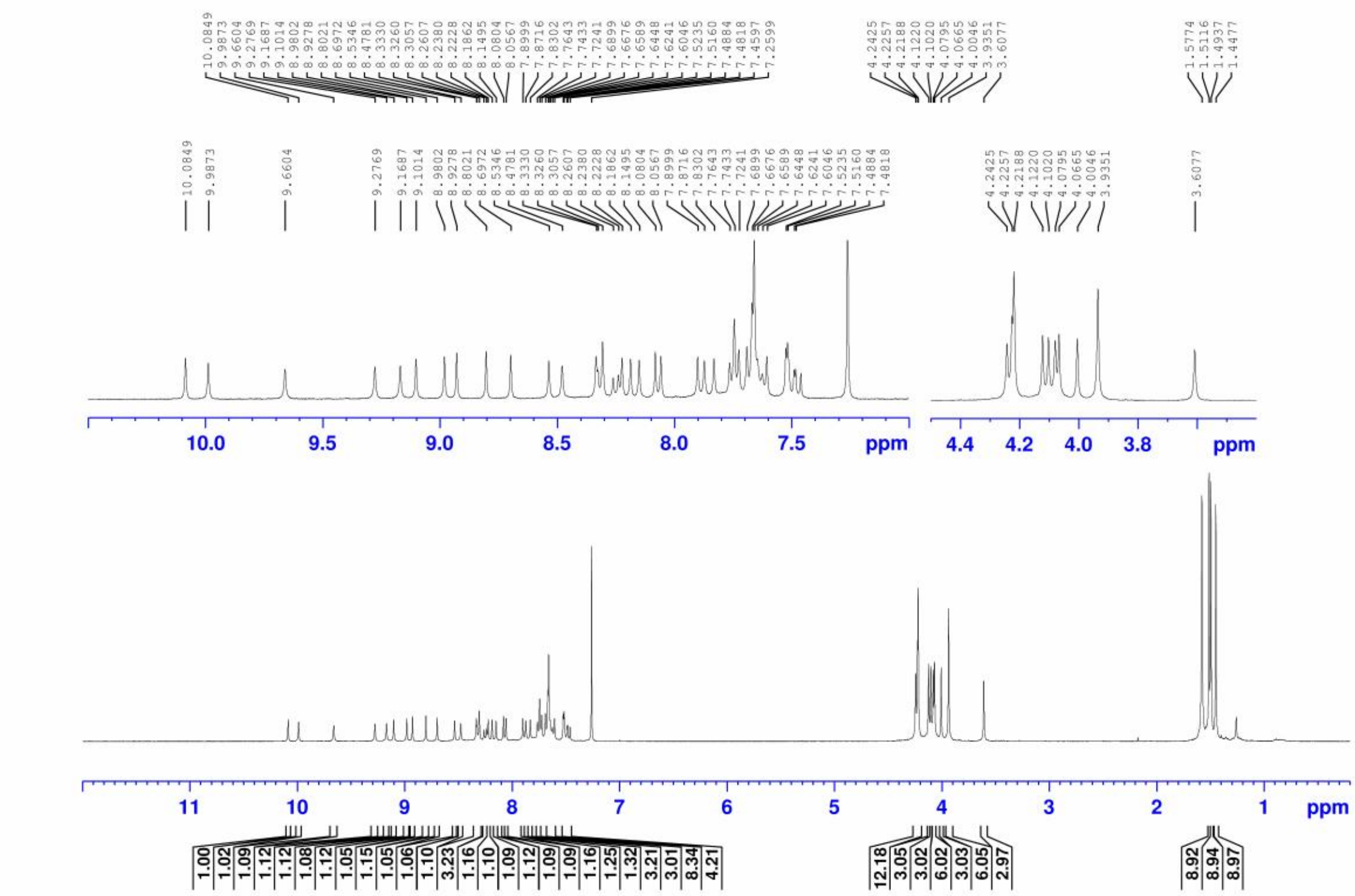

Figure S17. ^{1}H NMR spectrum (400MHz, $CDCl_3$) of **1**.

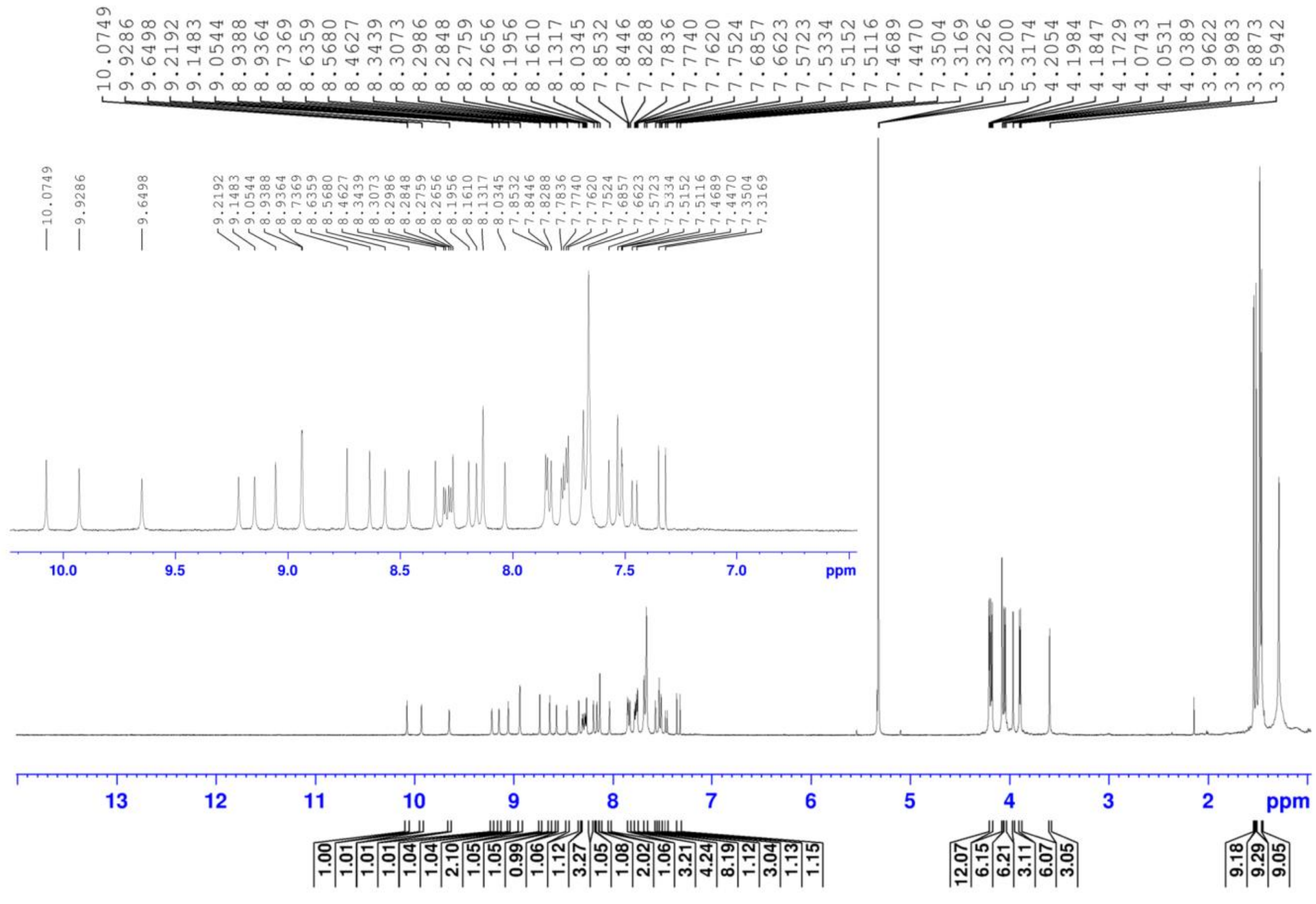

Figure S18. ^{1}H NMR spectrum (400MHz, CD_2Cl_2 + CS_2) of **1**.

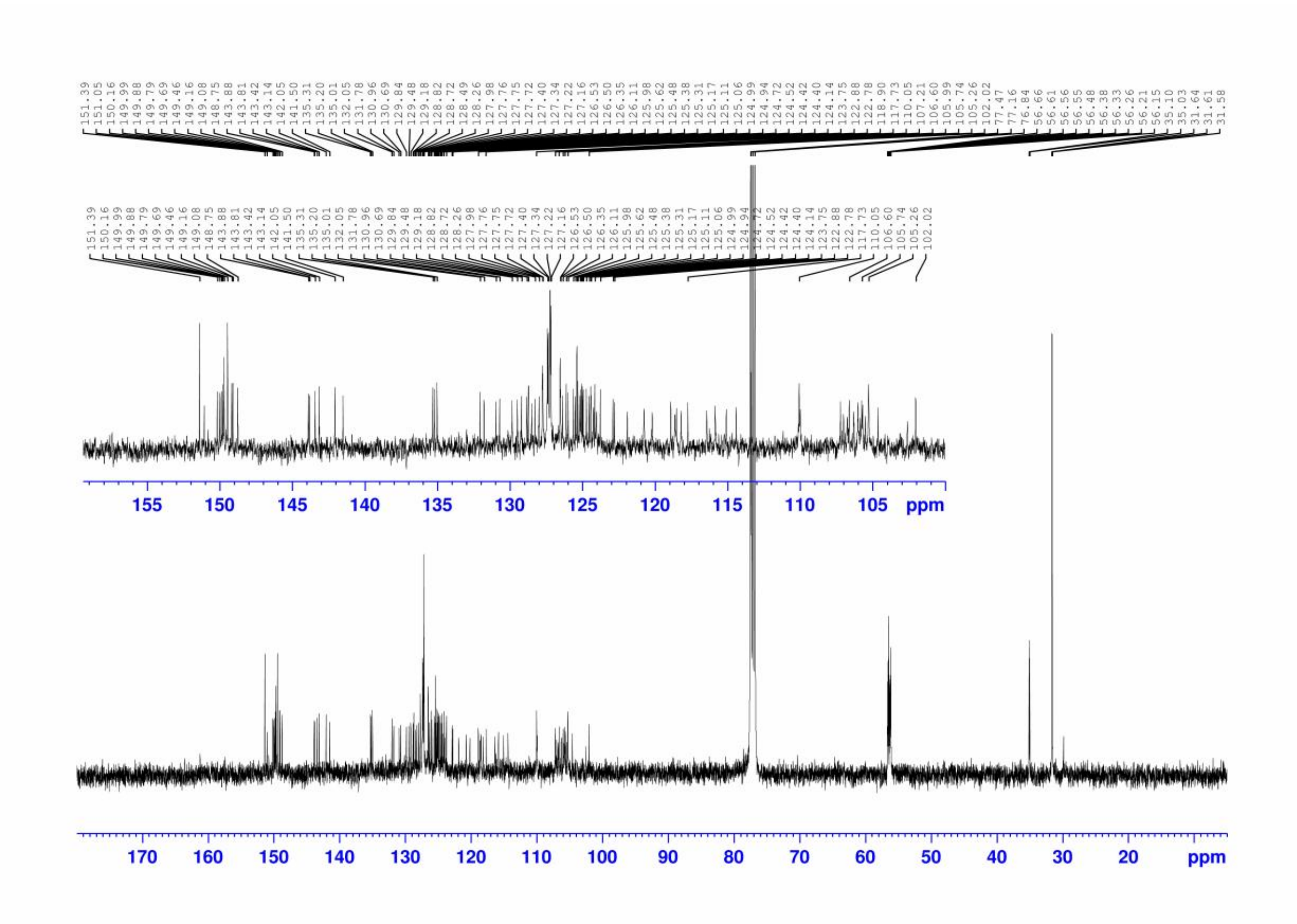

Figure S19. ^{13}C NMR spectrum (101MHz, $CDCl_3$) of **1**.

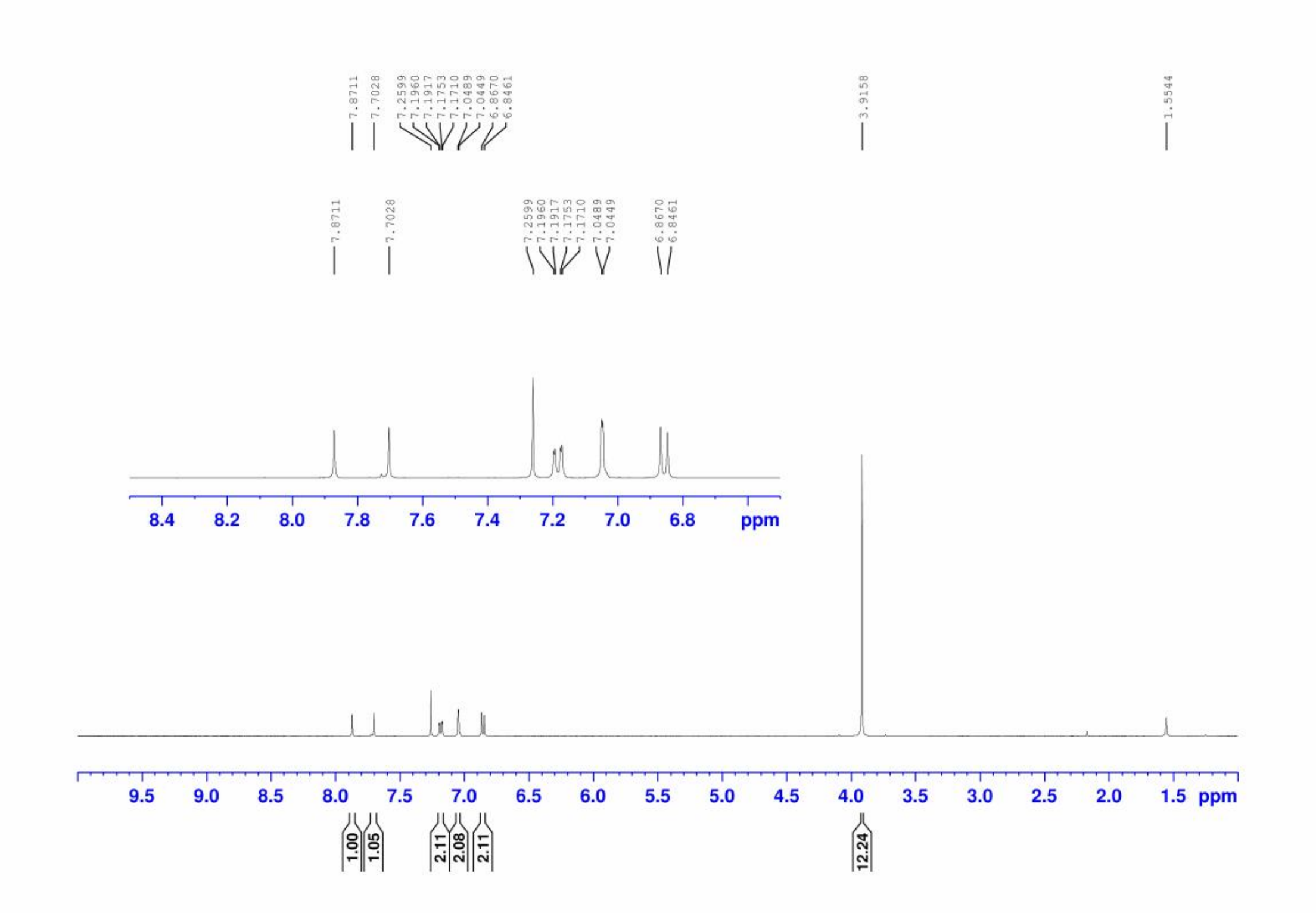

Figure S20. ^{1}H NMR spectrum (400MHz, $CDCl_3$) of **15a**.

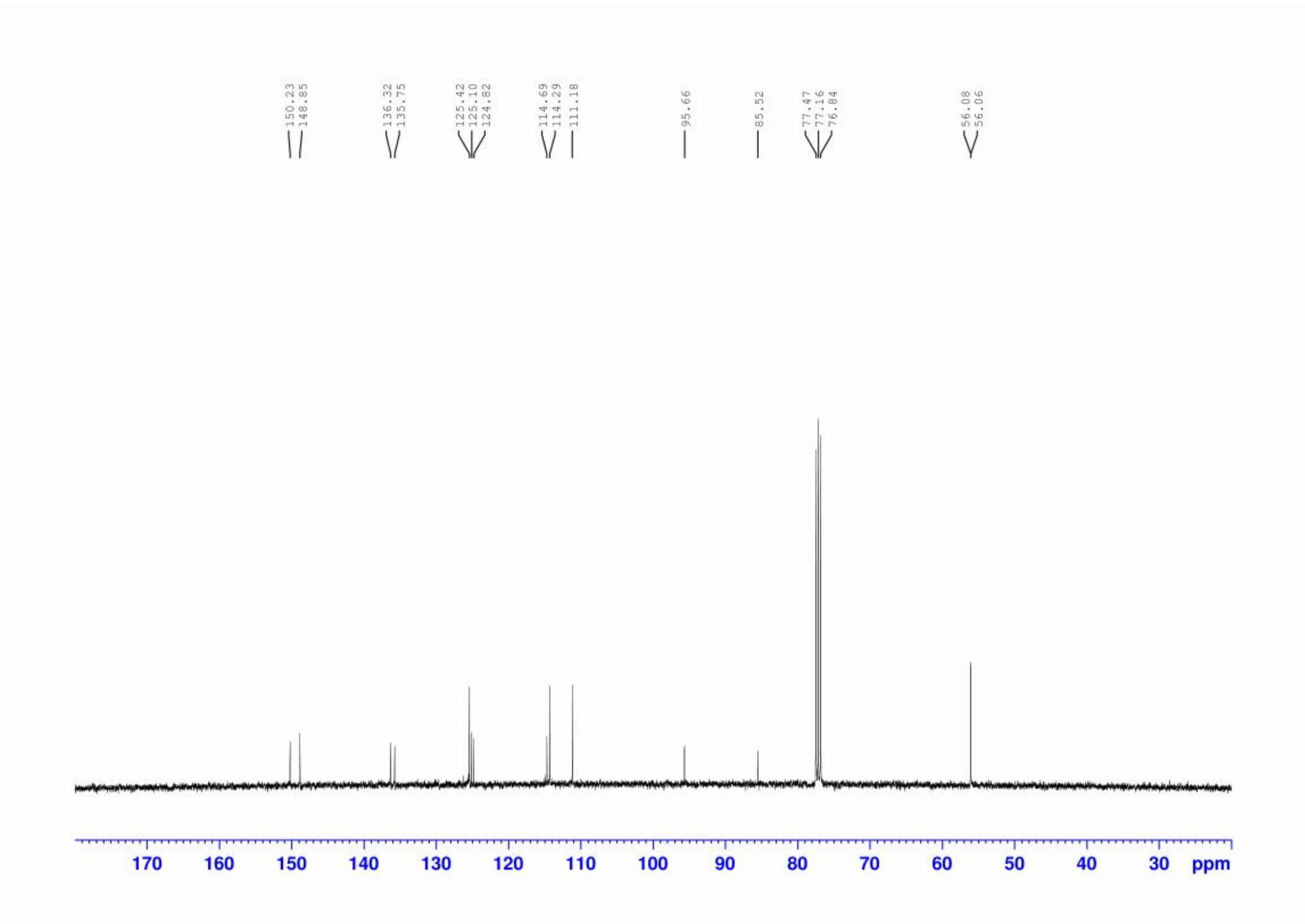

Figure S21. ^{13}C NMR spectrum (101MHz, $CDCl_3$) of **15a**.

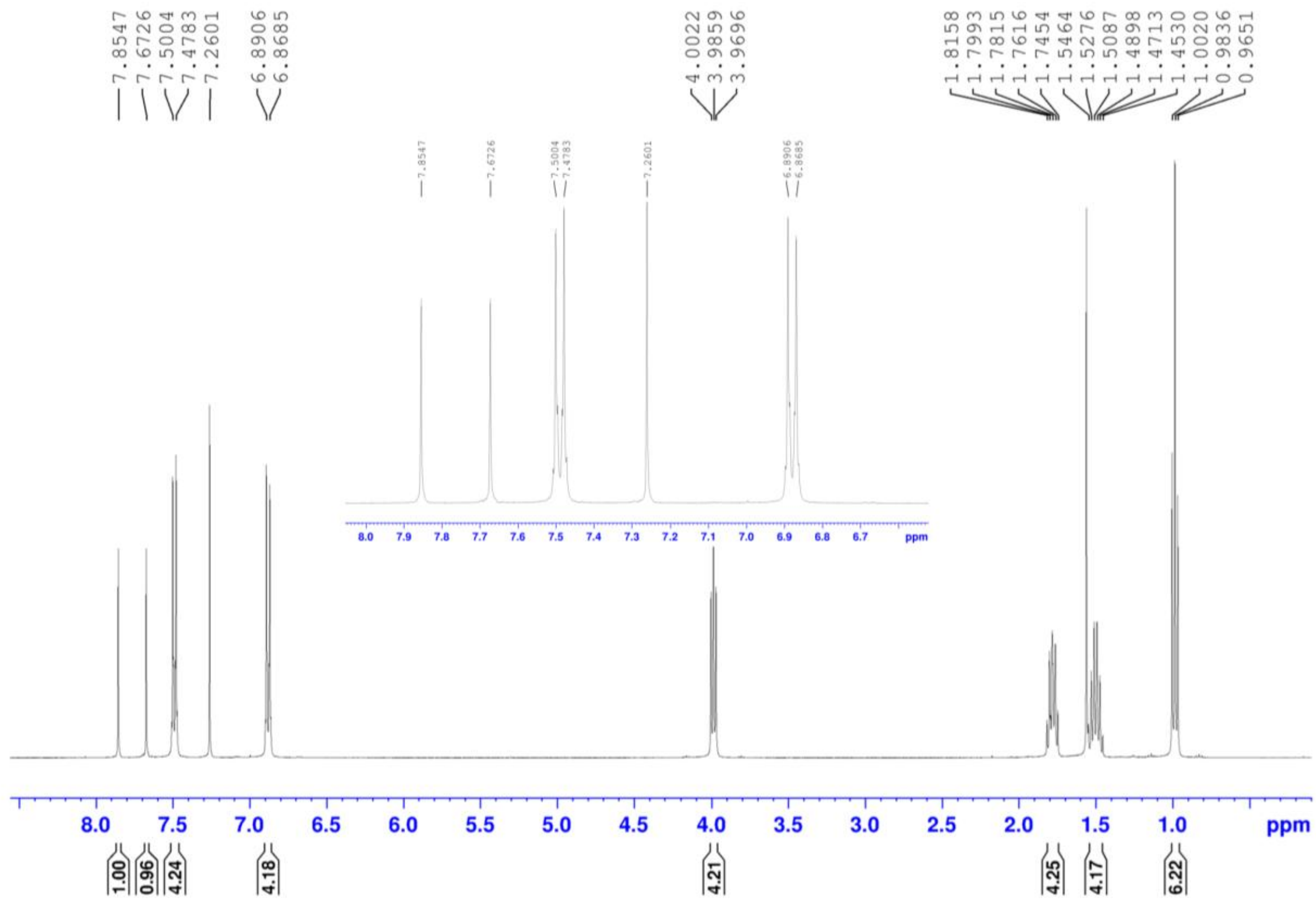

Figure S22. ^{1}H NMR spectrum (400MHz, $CDCl_3$) of **15b**.

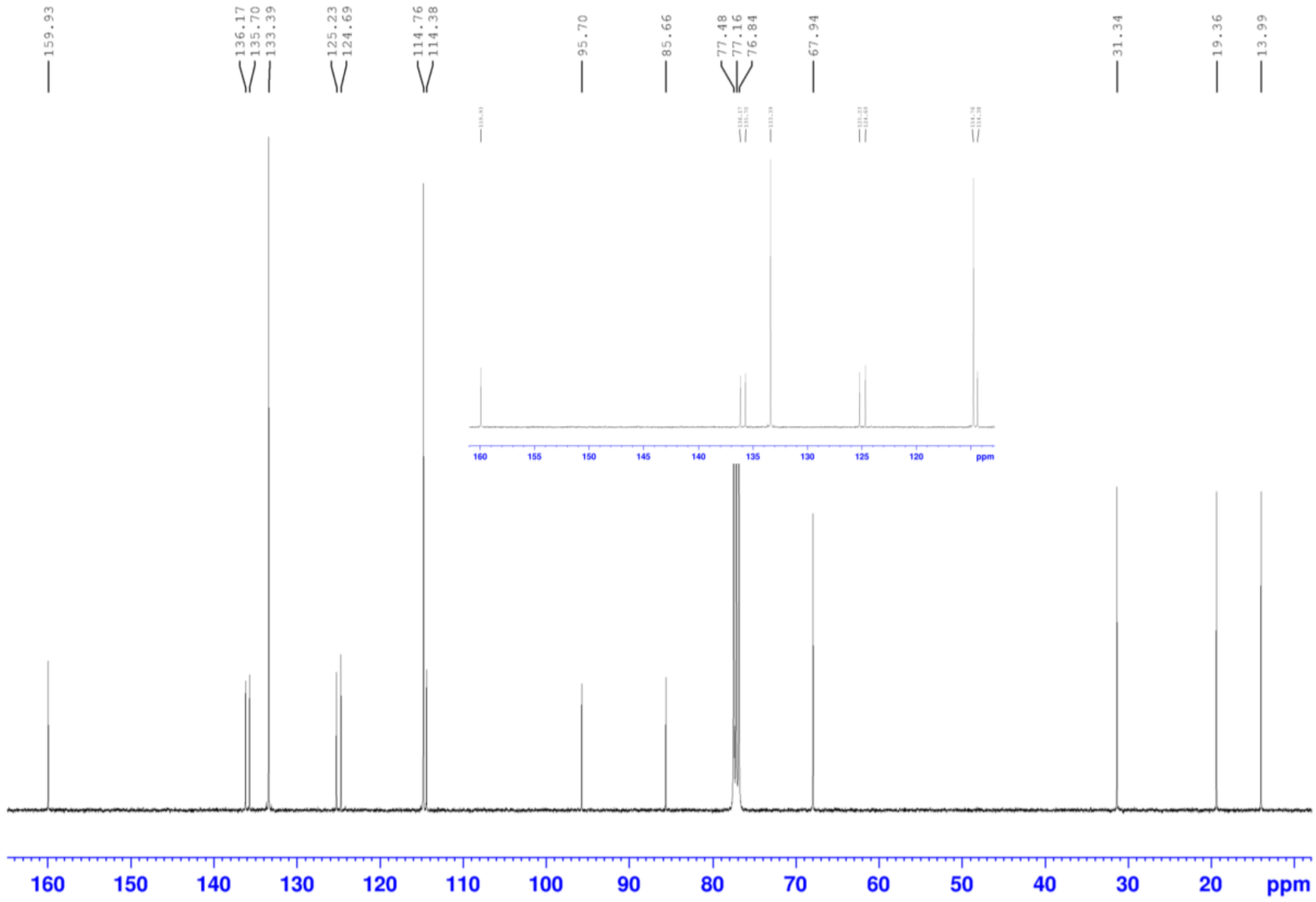

Figure S23. ^{13}C NMR spectrum (101MHz, $CDCl_3$) of **15b**.

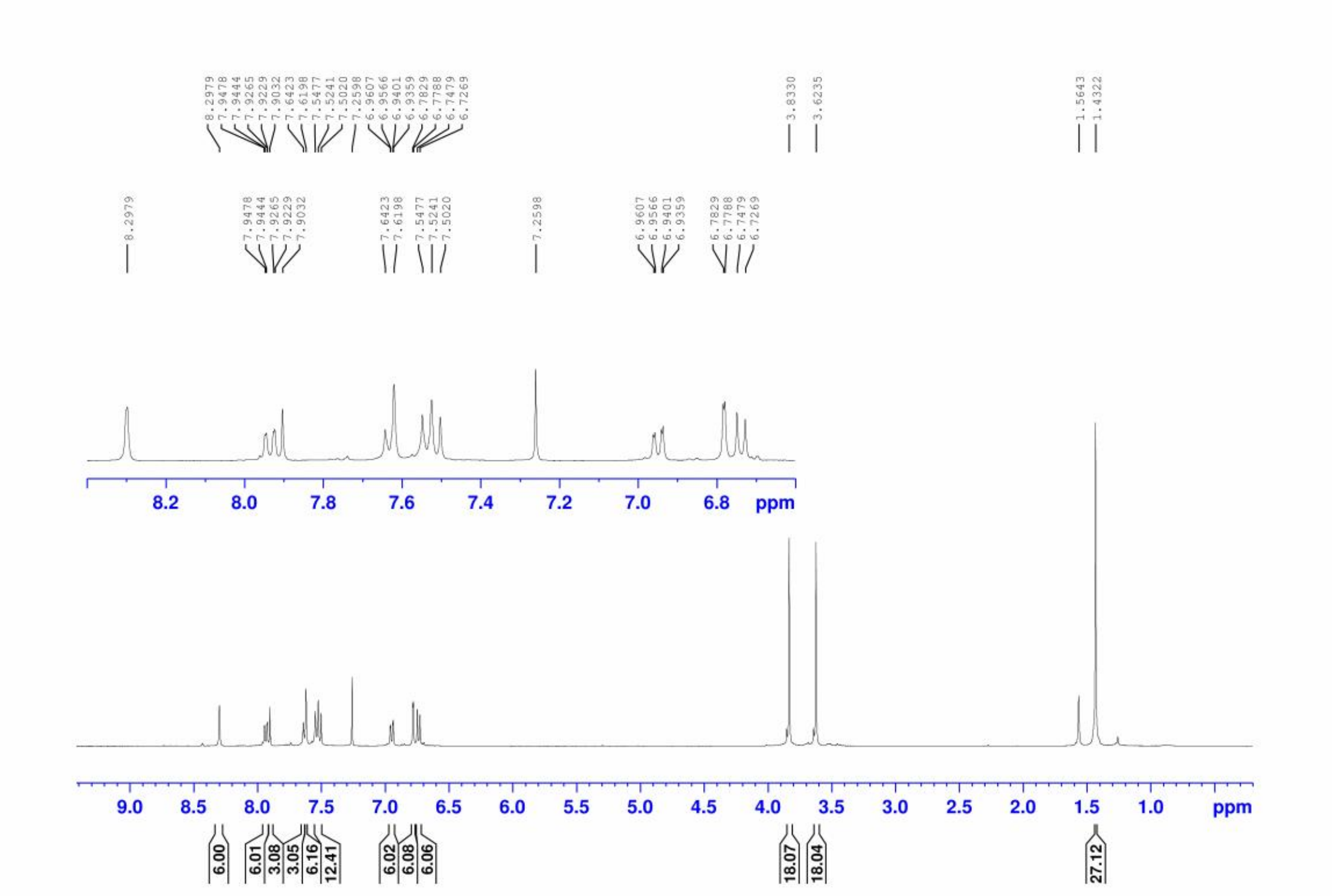

Figure S24. ^{1}H NMR spectrum (400MHz, $CDCl_3$) of **14a**.

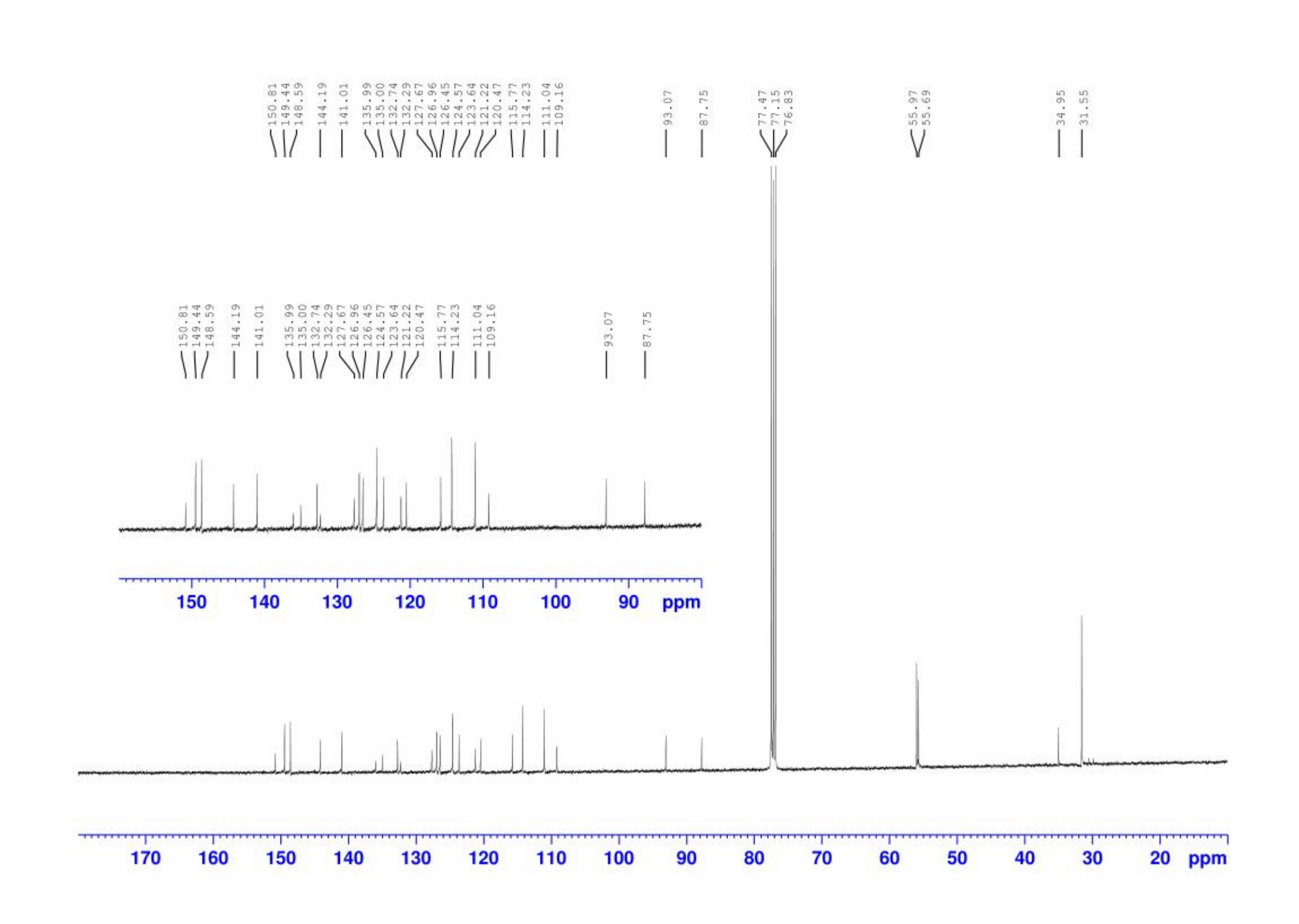

Figure S25. ^{13}C NMR spectrum (101MHz, $CDCl_3$) of **14a**.

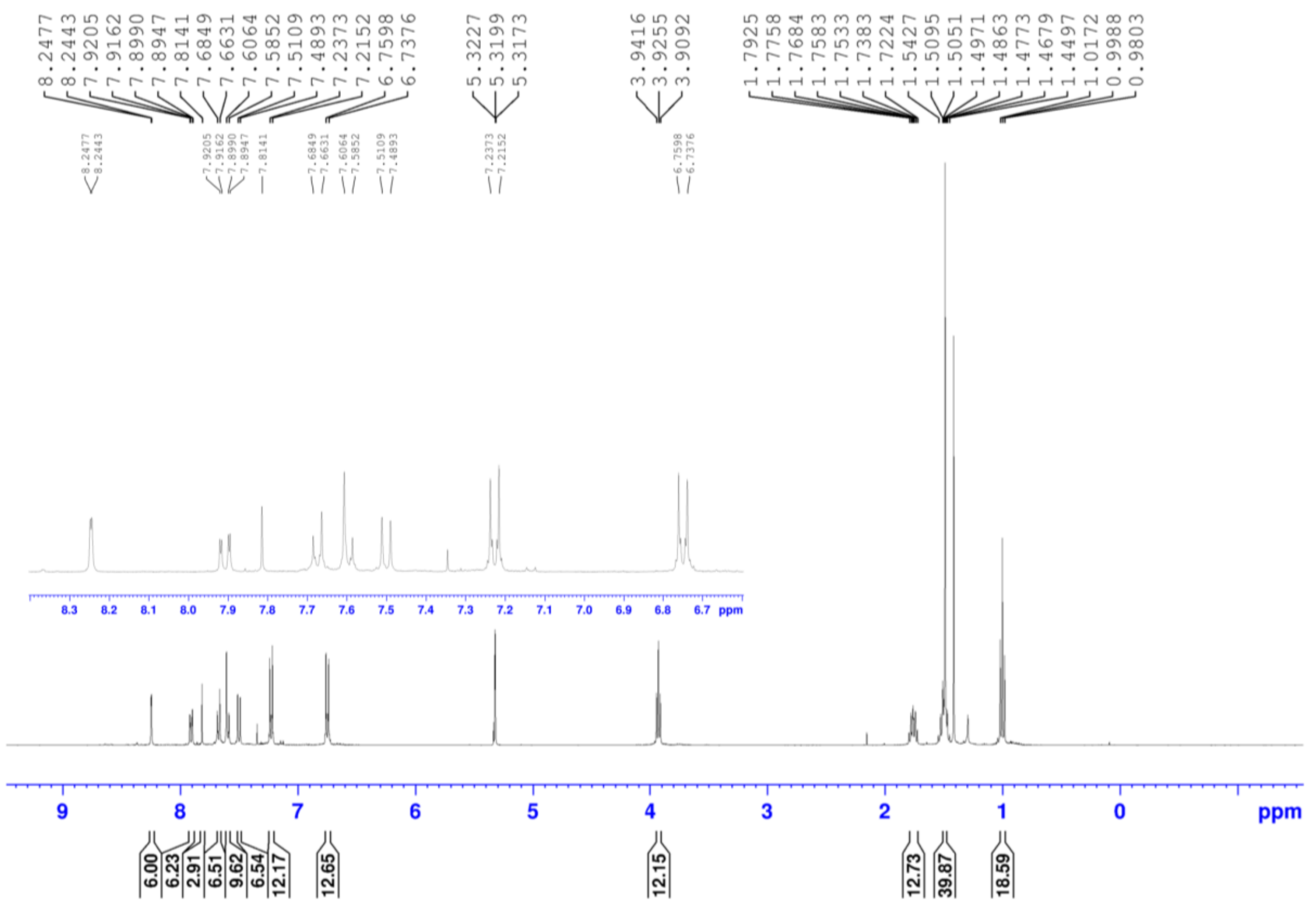

Figure S26. ^{1}H NMR spectrum (400MHz, $CD_2Cl_2 + CS_2$) of **14b**.

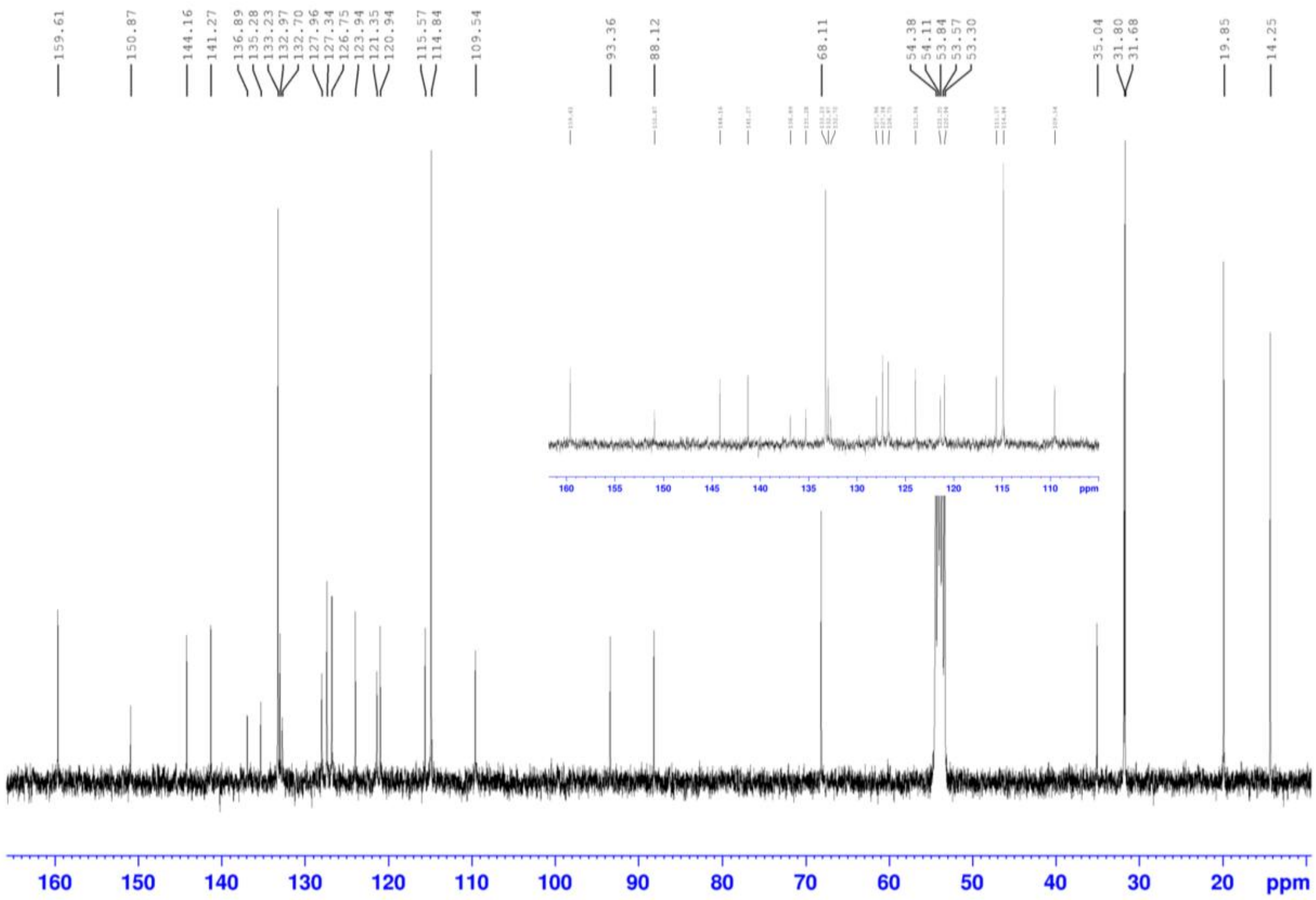

Figure S27. ^{13}C NMR spectrum (101MHz, CD_2Cl_2 + CS_2) of **14b**.

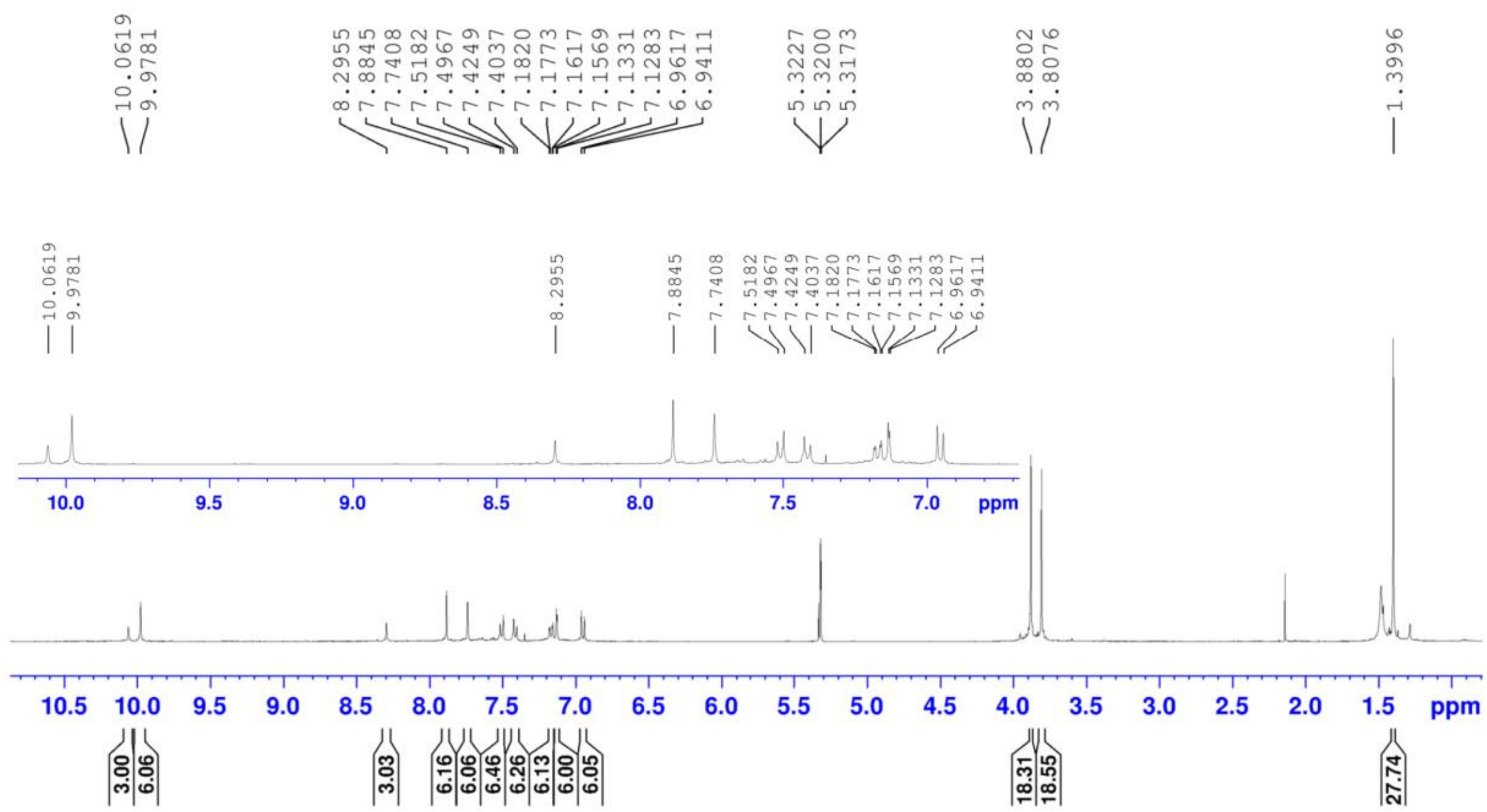

Figure S28. ^{1}H NMR spectrum (400MHz, CD_2Cl_2 + CS_2) of **2a**.

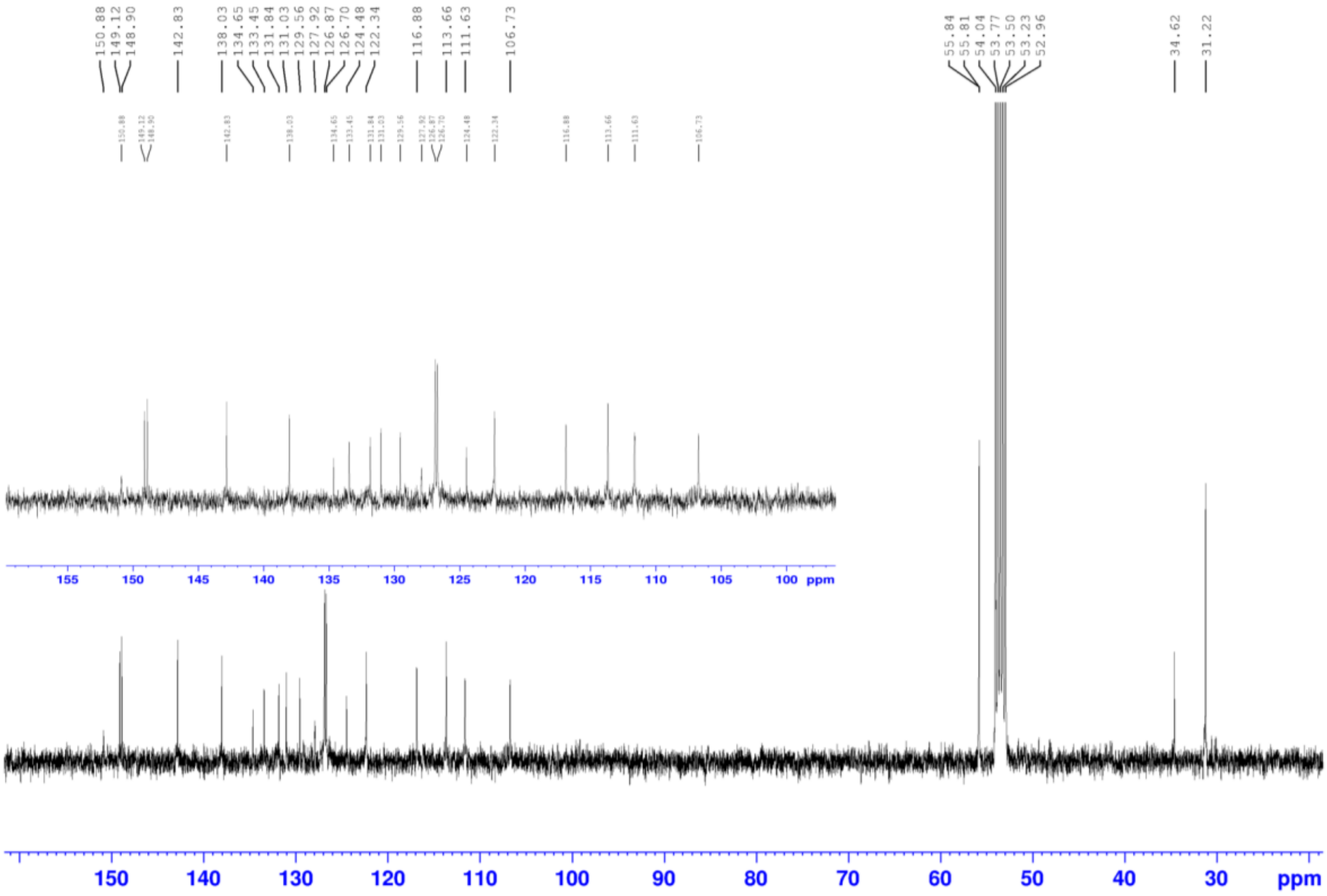

Figure S29. ^{13}C NMR spectrum (101MHz, CD_2Cl_2 + CS_2) of **2a**.

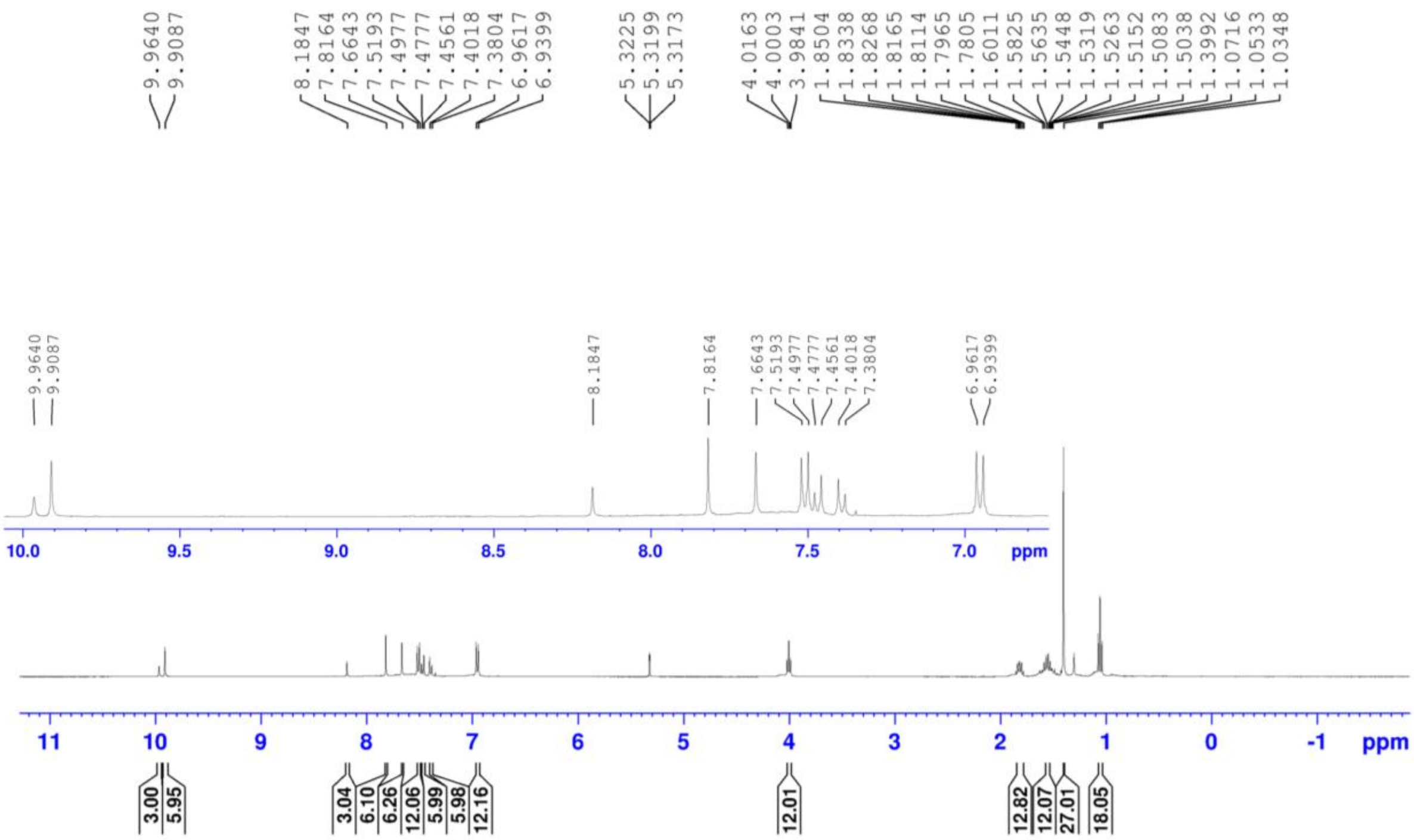

Figure S30. ^{1}H NMR spectrum (400MHz, CD_2Cl_2 + CS_2) of **2b**.

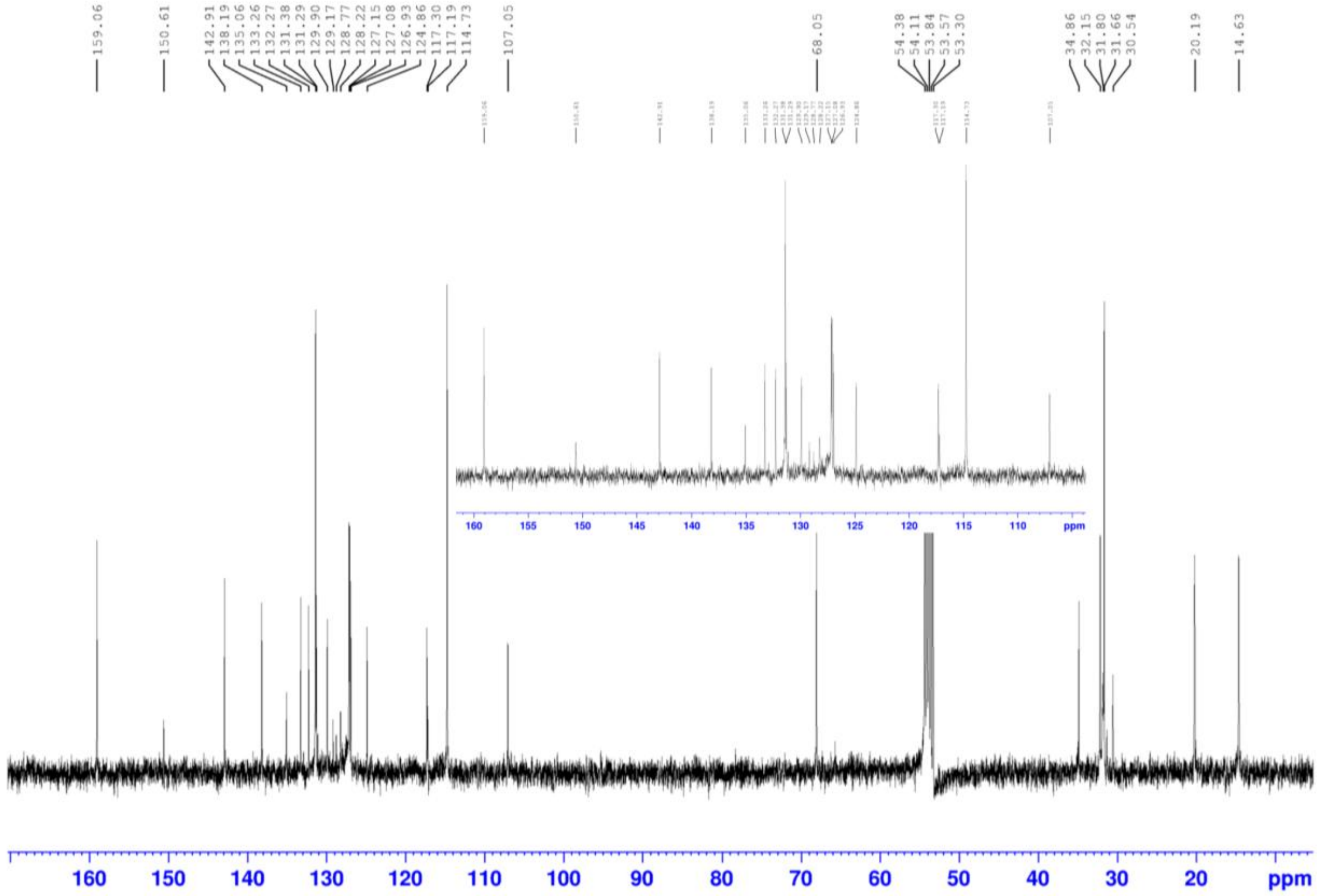

Figure S31. ^{13}C NMR spectrum (101MHz, CD_2Cl_2 + CS_2) of **2b**.

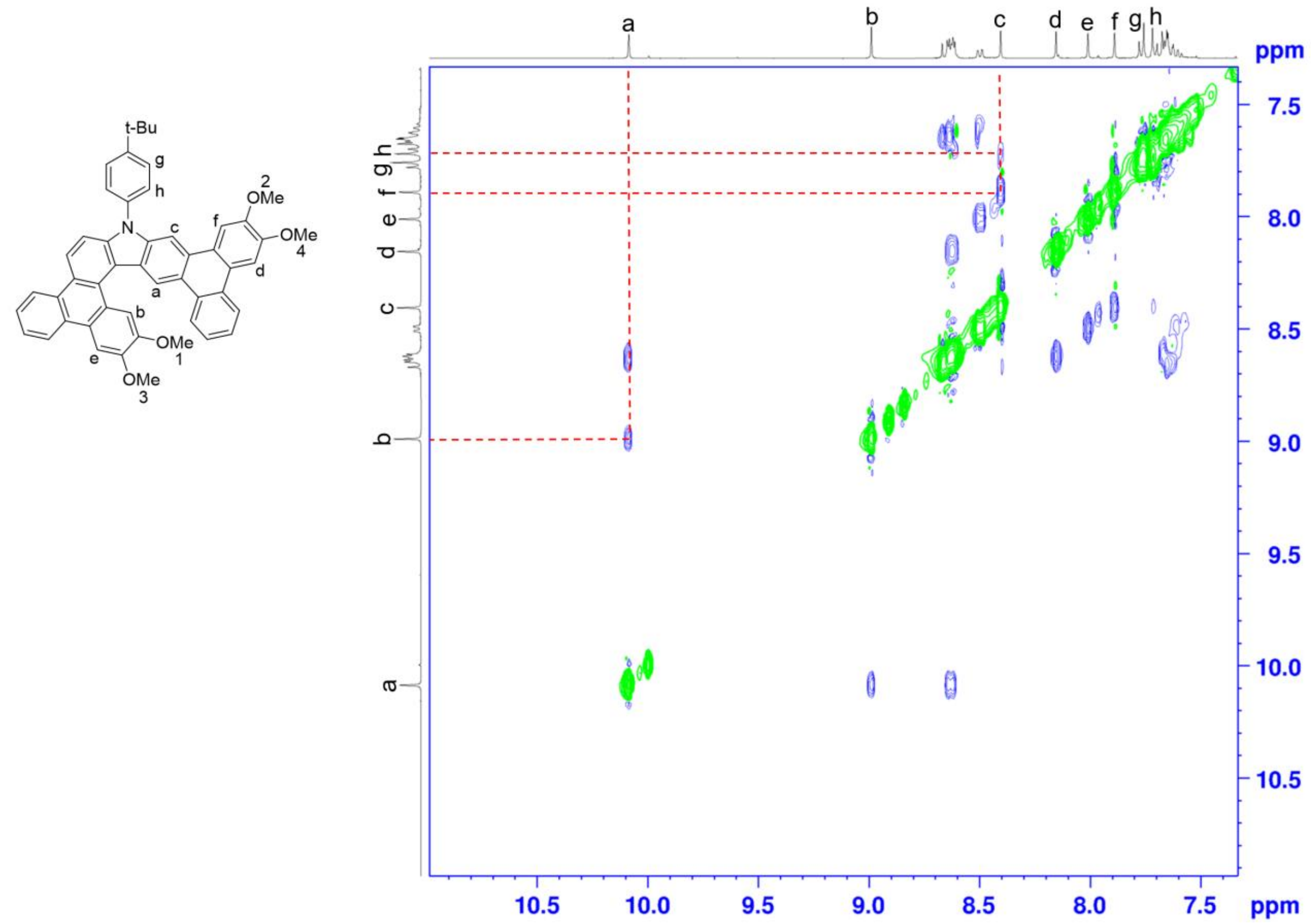

Figure S32. ROESY spectrum of **5** in $CDCl_3$ at 298K (aromatic region).

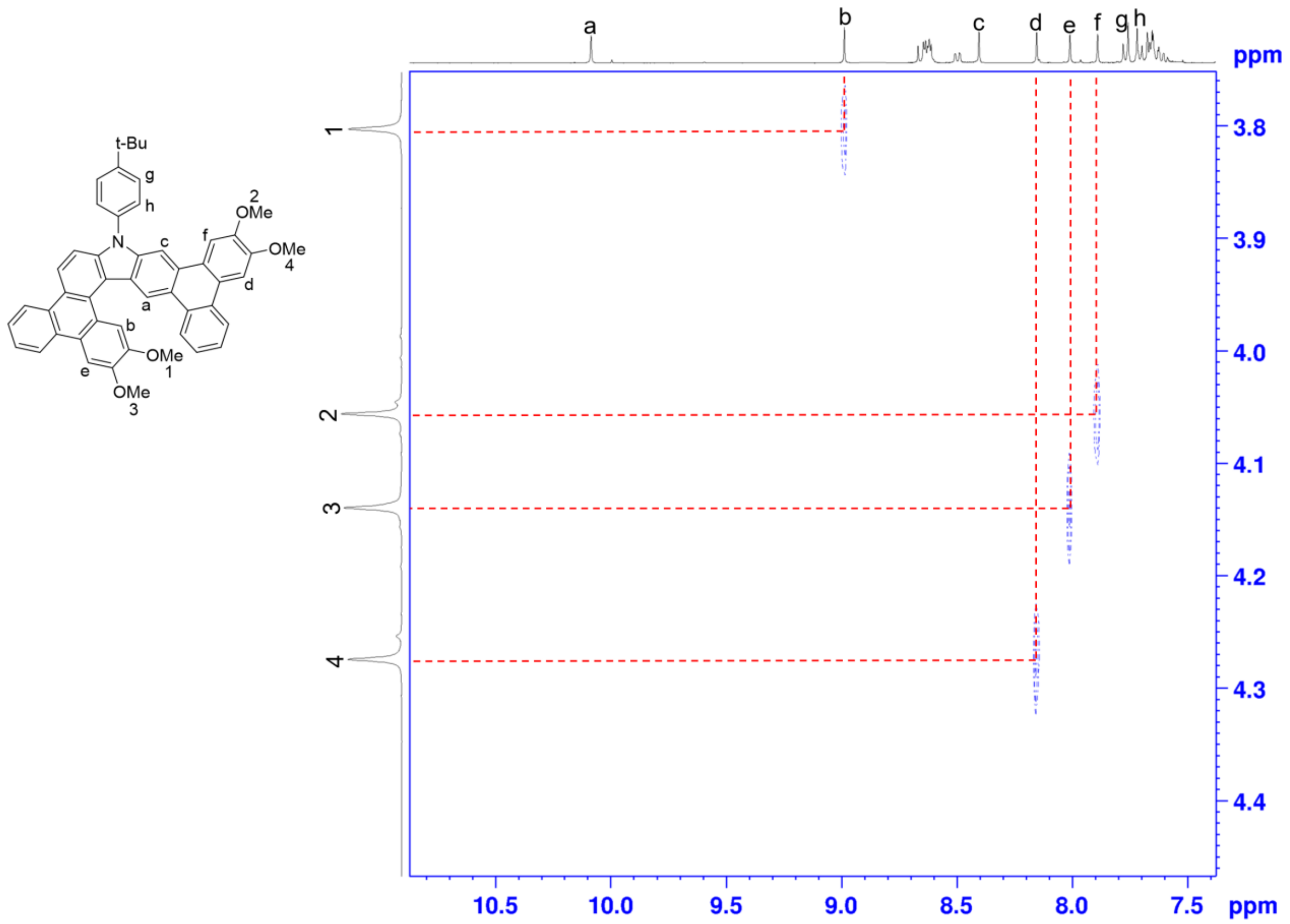

Figure S33. ROESY spectrum of **5** in $CDCl_3$ at 298K (aromatic region versus aliphatic region).

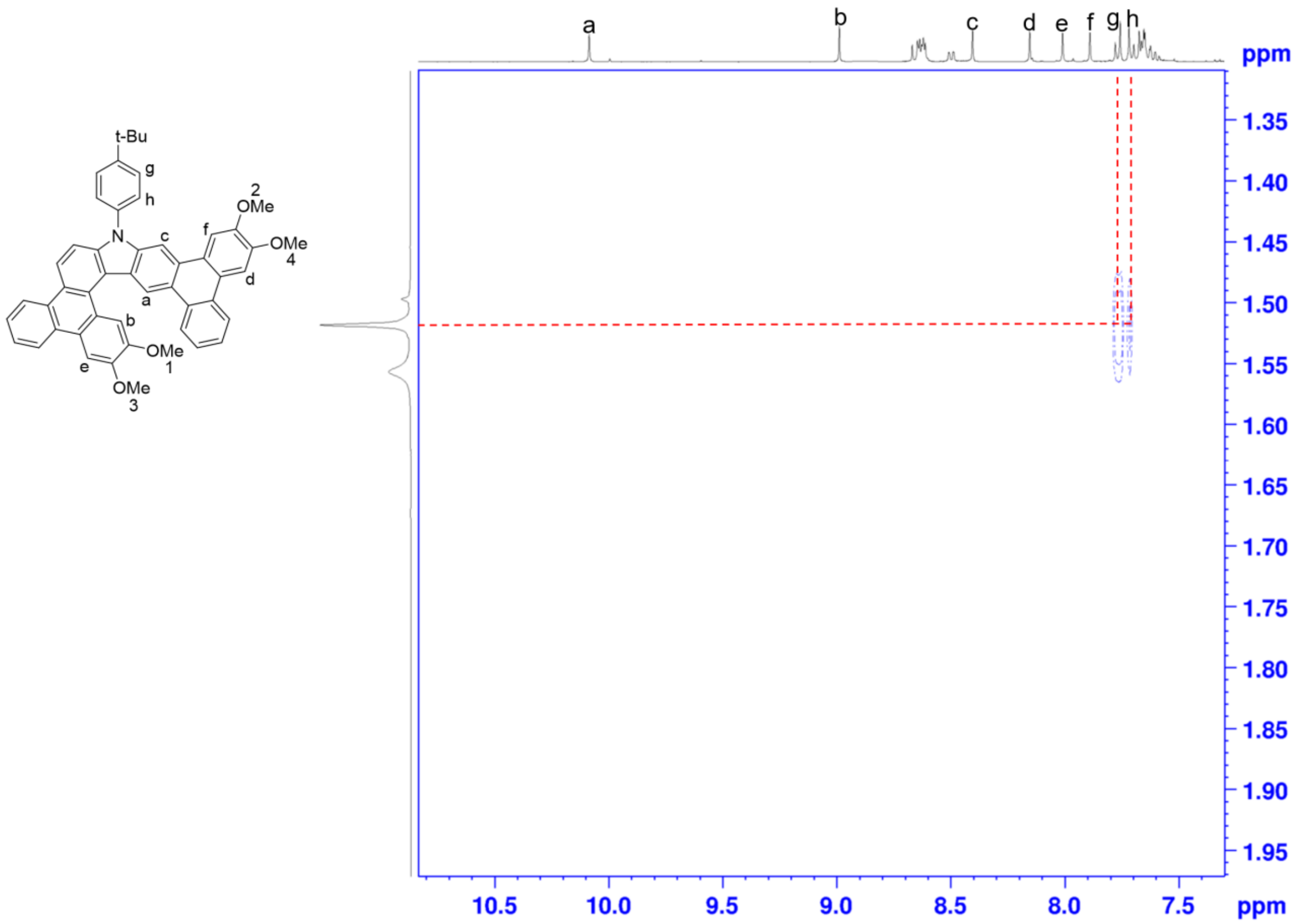

Figure S34. ROESY spectrum of **5** in $CDCl_3$ at 298K (aromatic region versus *t*-Bu groups).

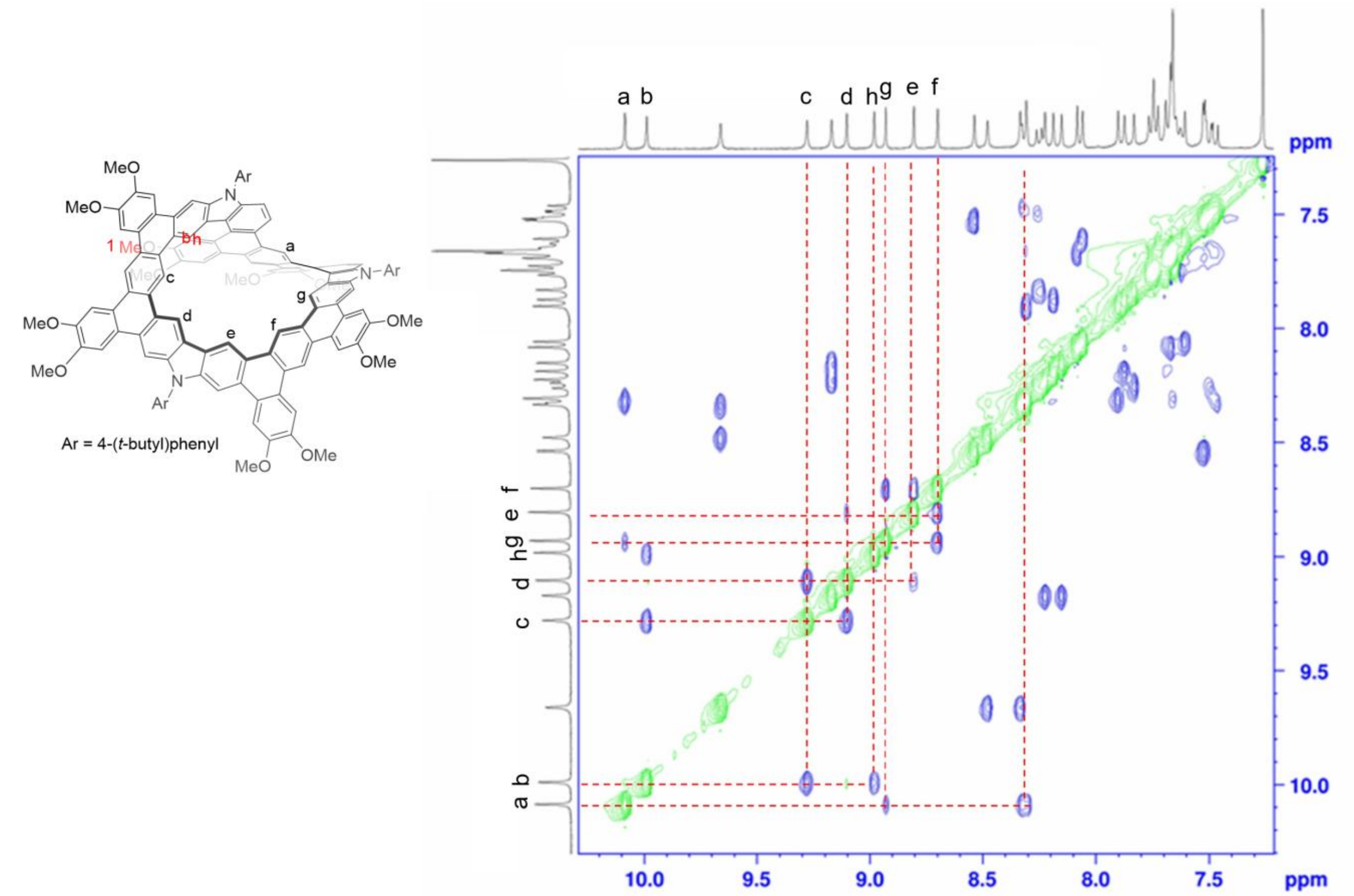

Figure S35. ROESY spectrum of **1** in $CDCl_3$ at 298K (aromatic region).

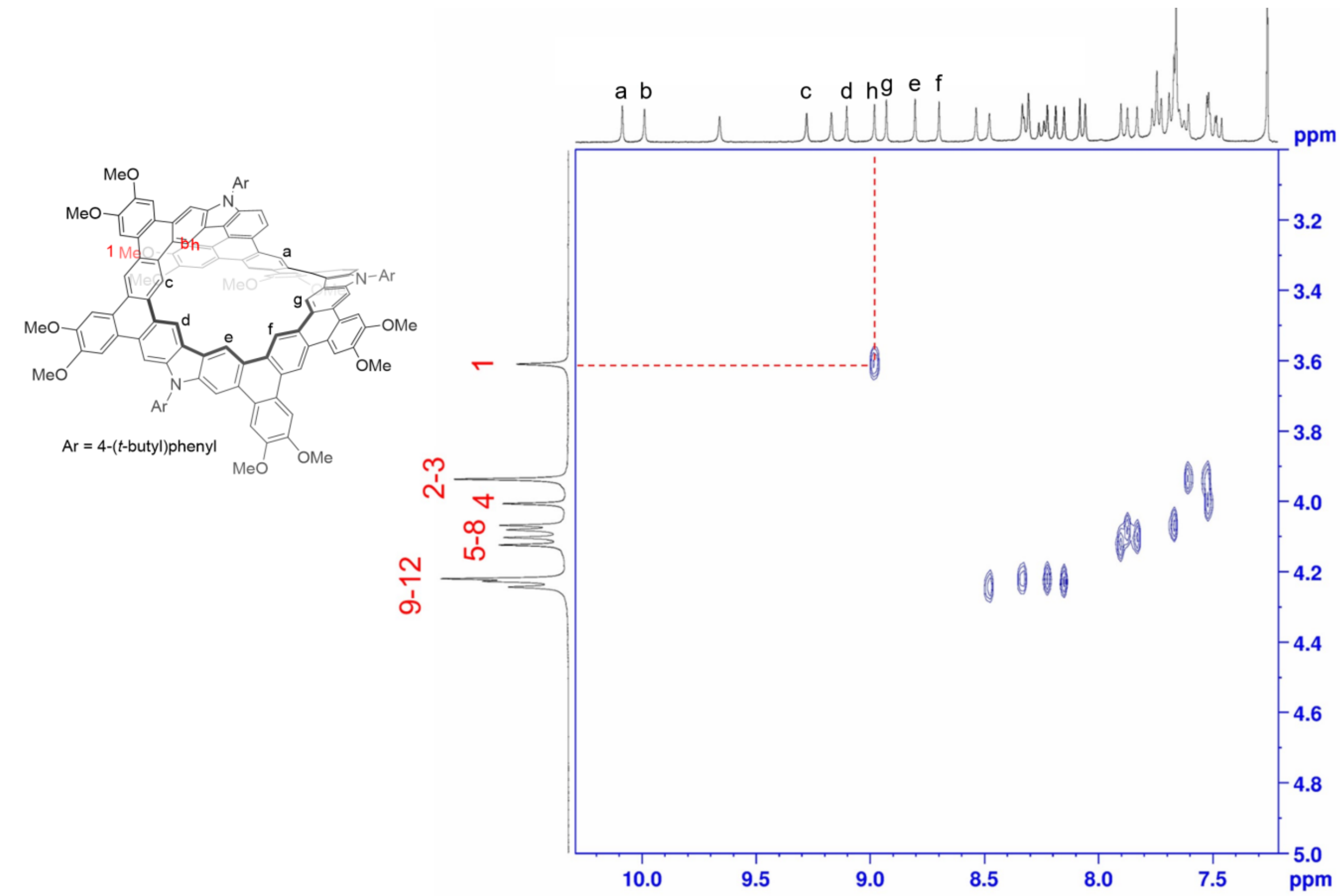

Figure S36. ROESY spectrum of **1** in $CDCl_3$ at 298K (aromatic region versus aliphatic region).

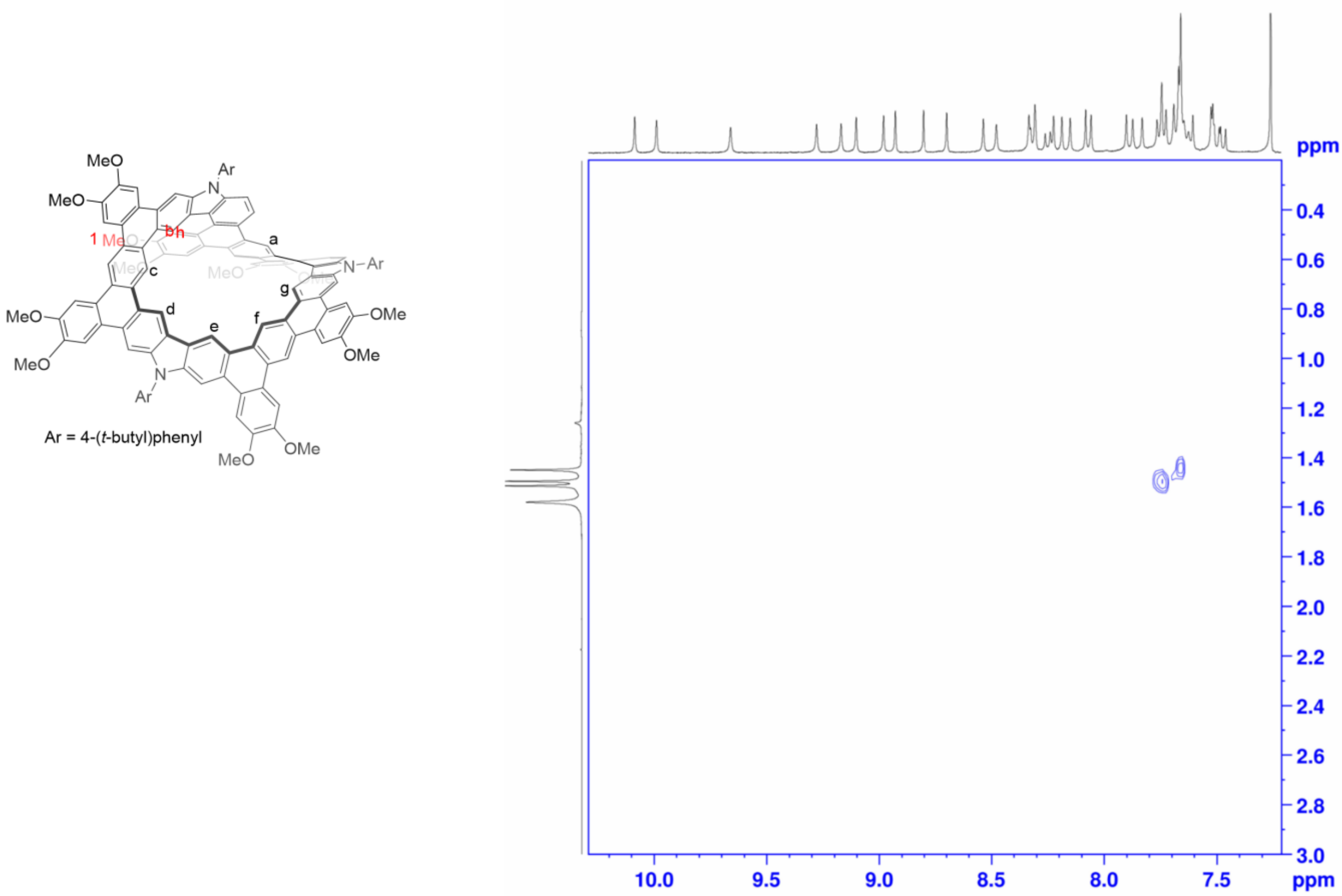

Figure S37. ROESY spectrum of **1** in $CDCl_3$ at 298K (aromatic region versus *t*-Bu groups).

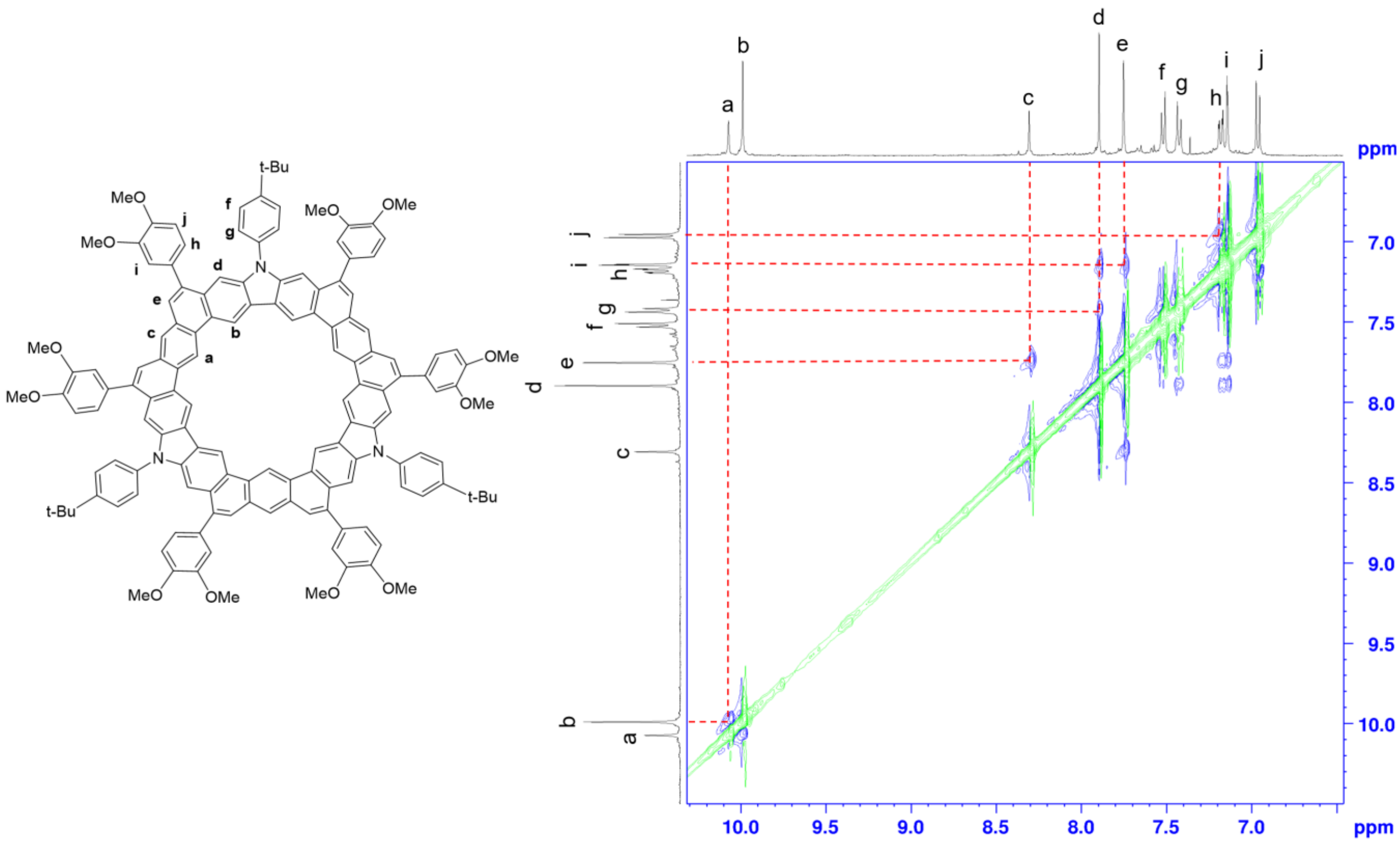

Figure S38. ROESY spectrum of **2a** in CD_2Cl_2 + CS_2 at 298K (aromatic region).

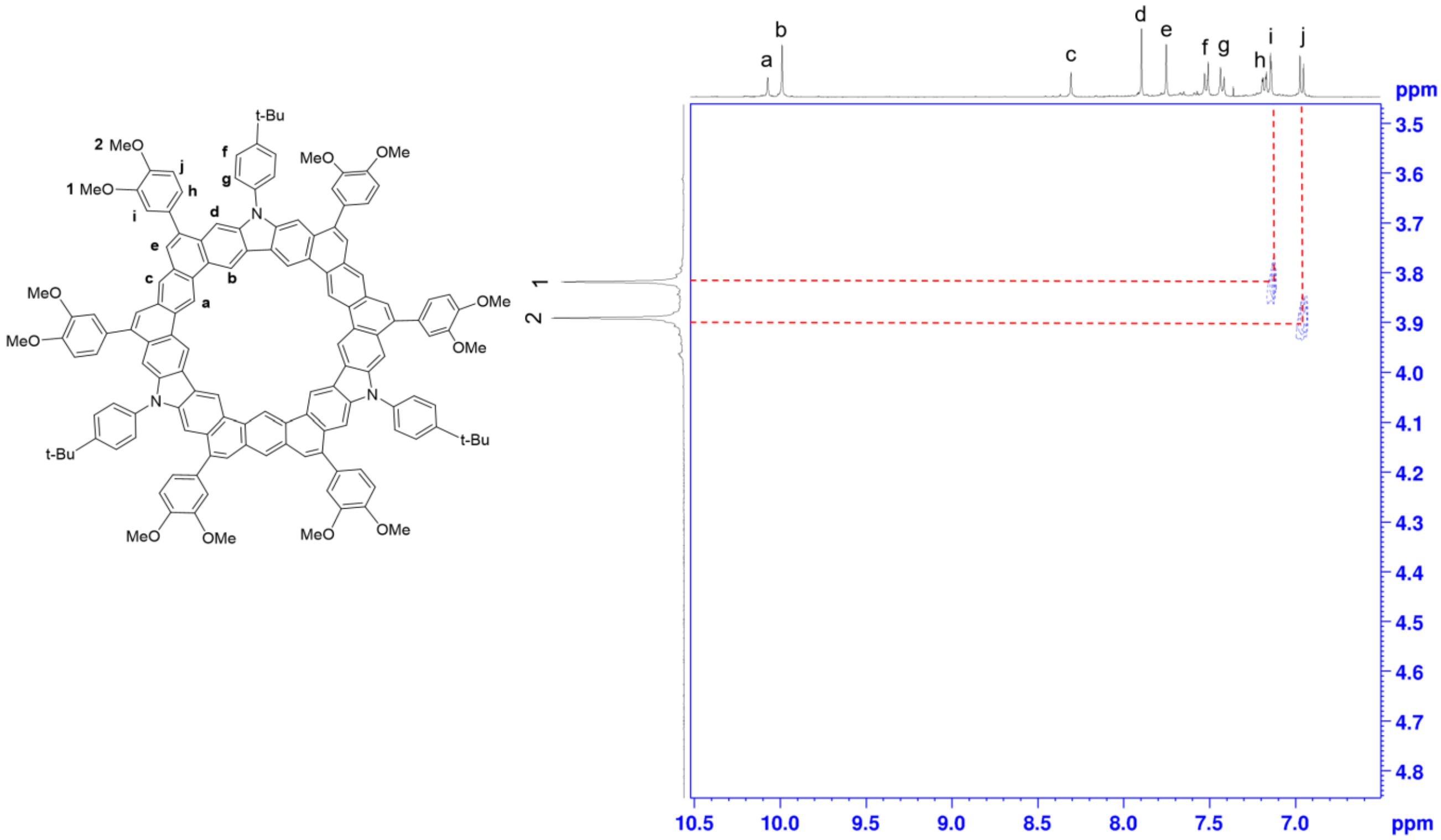

Figure S39. ROESY spectrum of **2a** in $CD_2Cl_2 + CS_2$ at 298K (aromatic region versus aliphatic region).

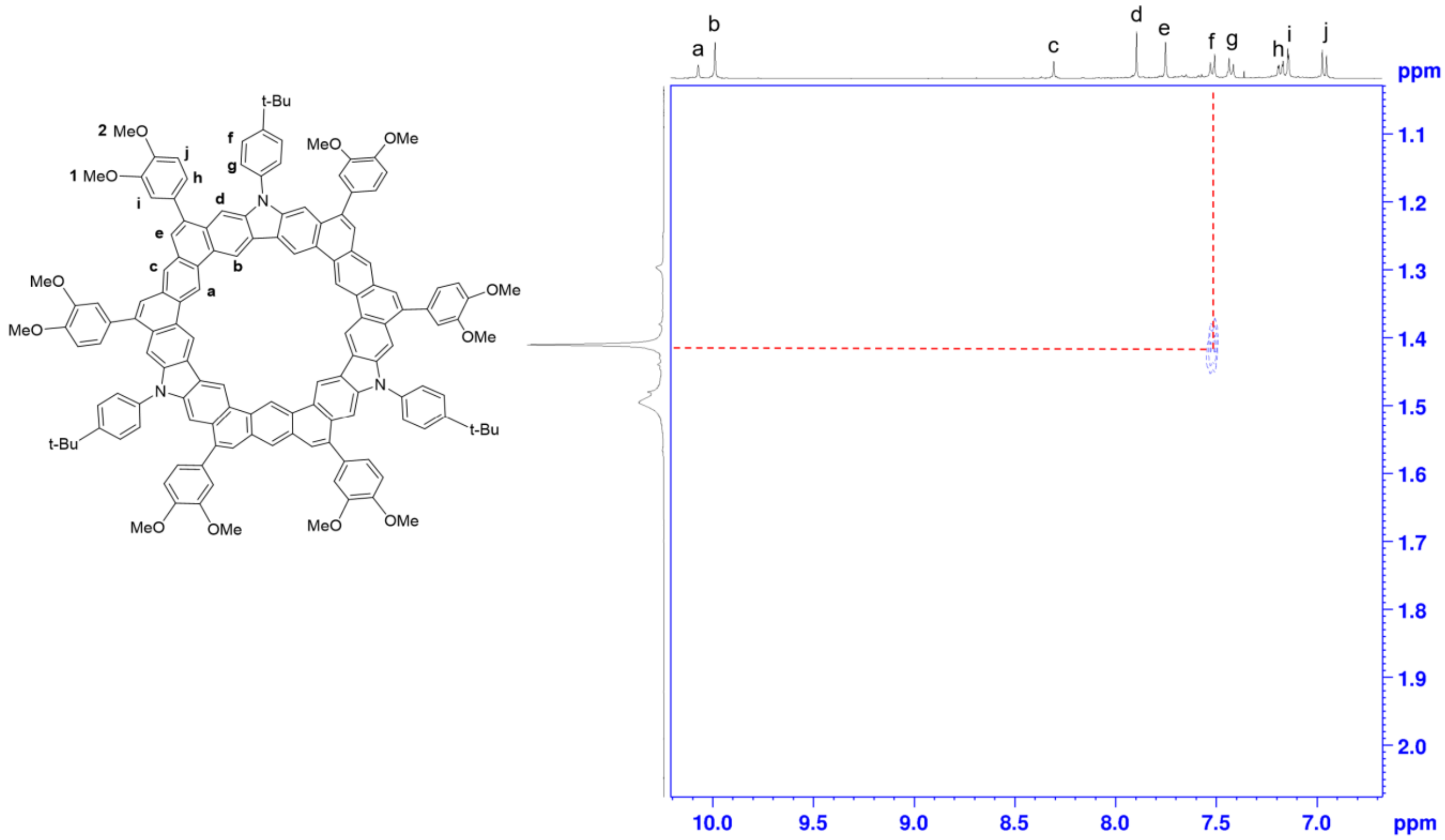

Figure S40. ROESY spectrum of **2a** in $CD_2Cl_2 + CS_2$ at 298K (aromatic region versus *t*-Bu groups).

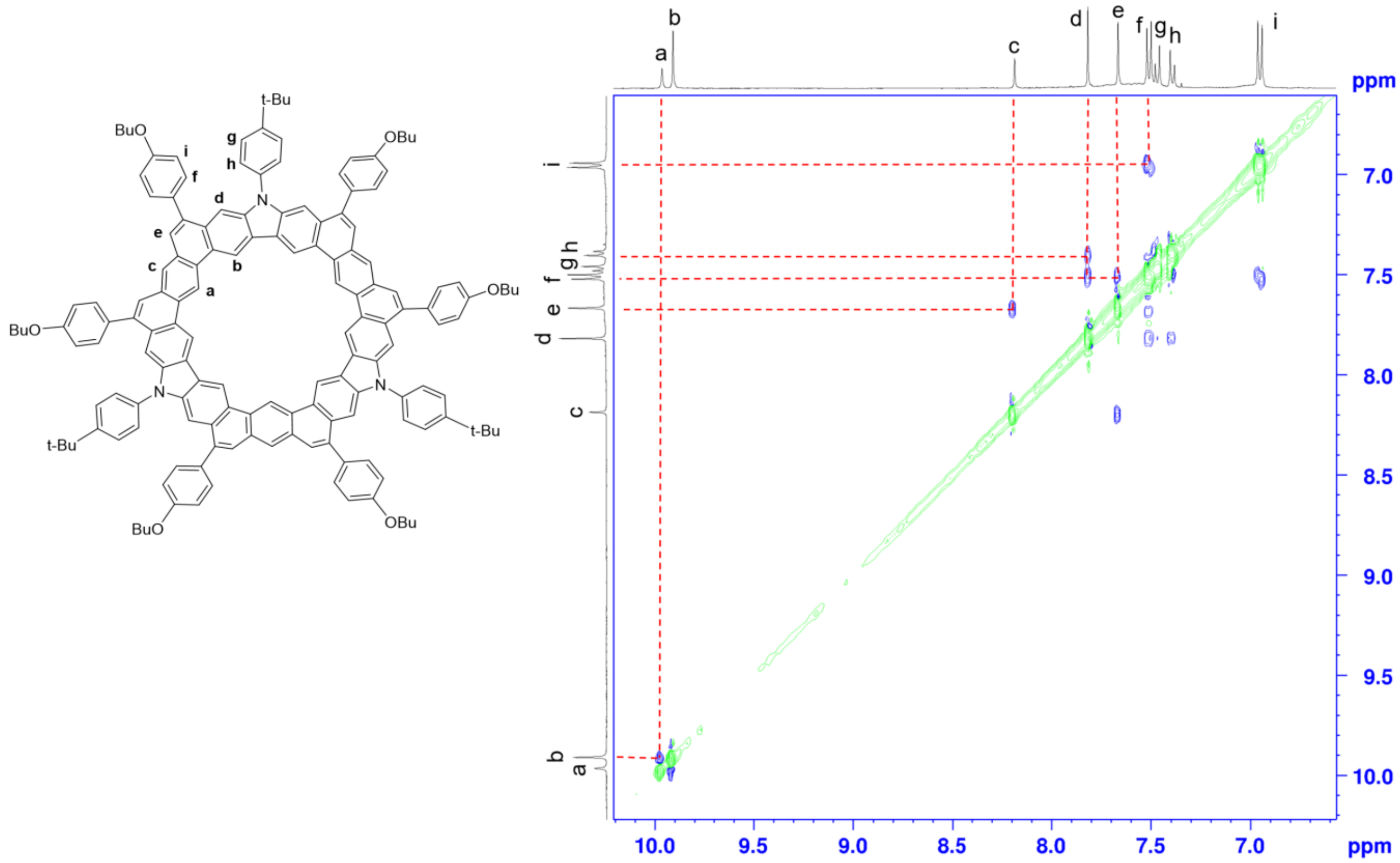

Figure S41. ROESY spectrum of **2b** in CD_2Cl_2 + CS_2 at 298K (aromatic region).

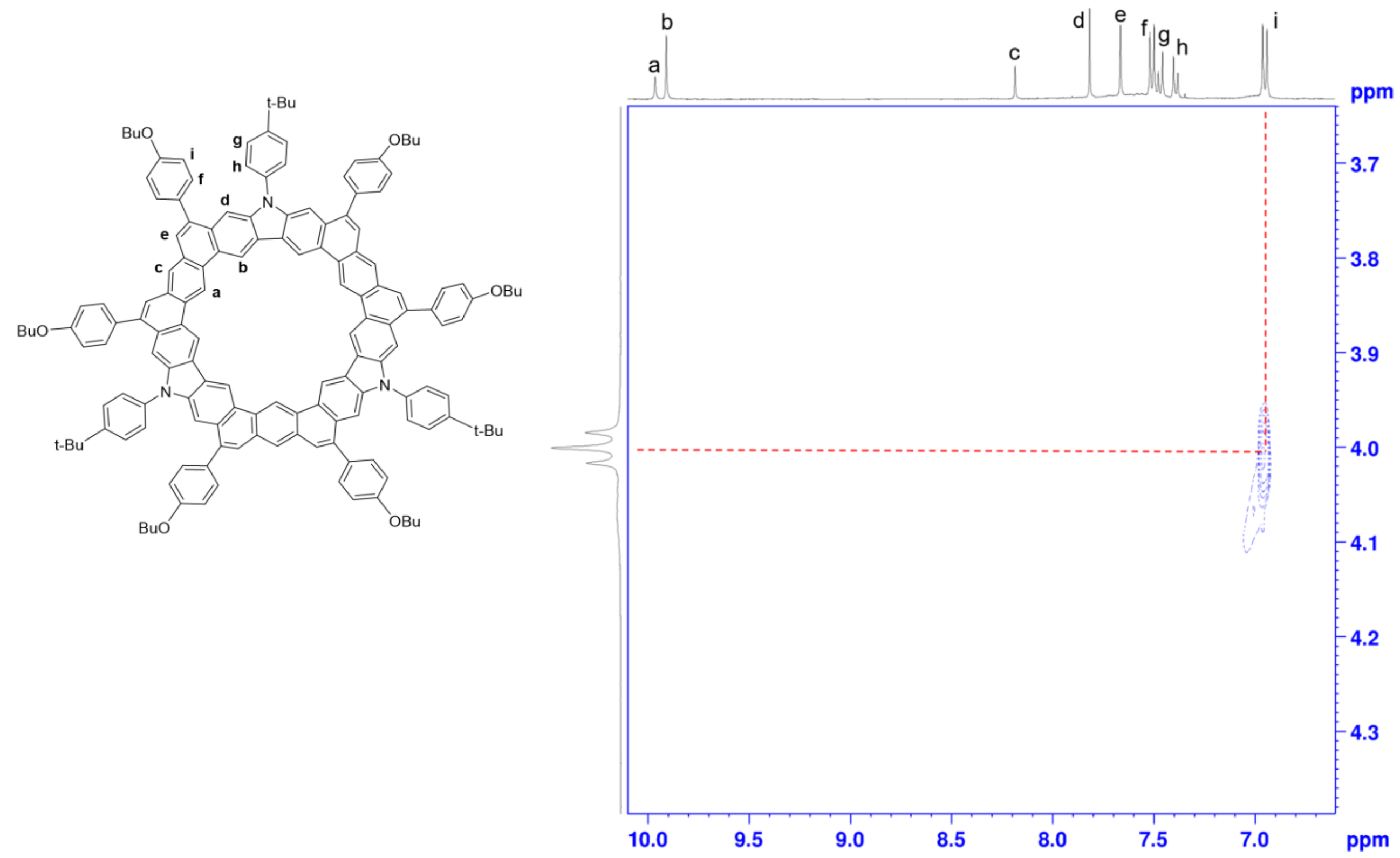

Figure S42. ROESY spectrum of **2b** in CD_2Cl_2 + CS_2 at 298K (aromatic region versus aliphatic region).

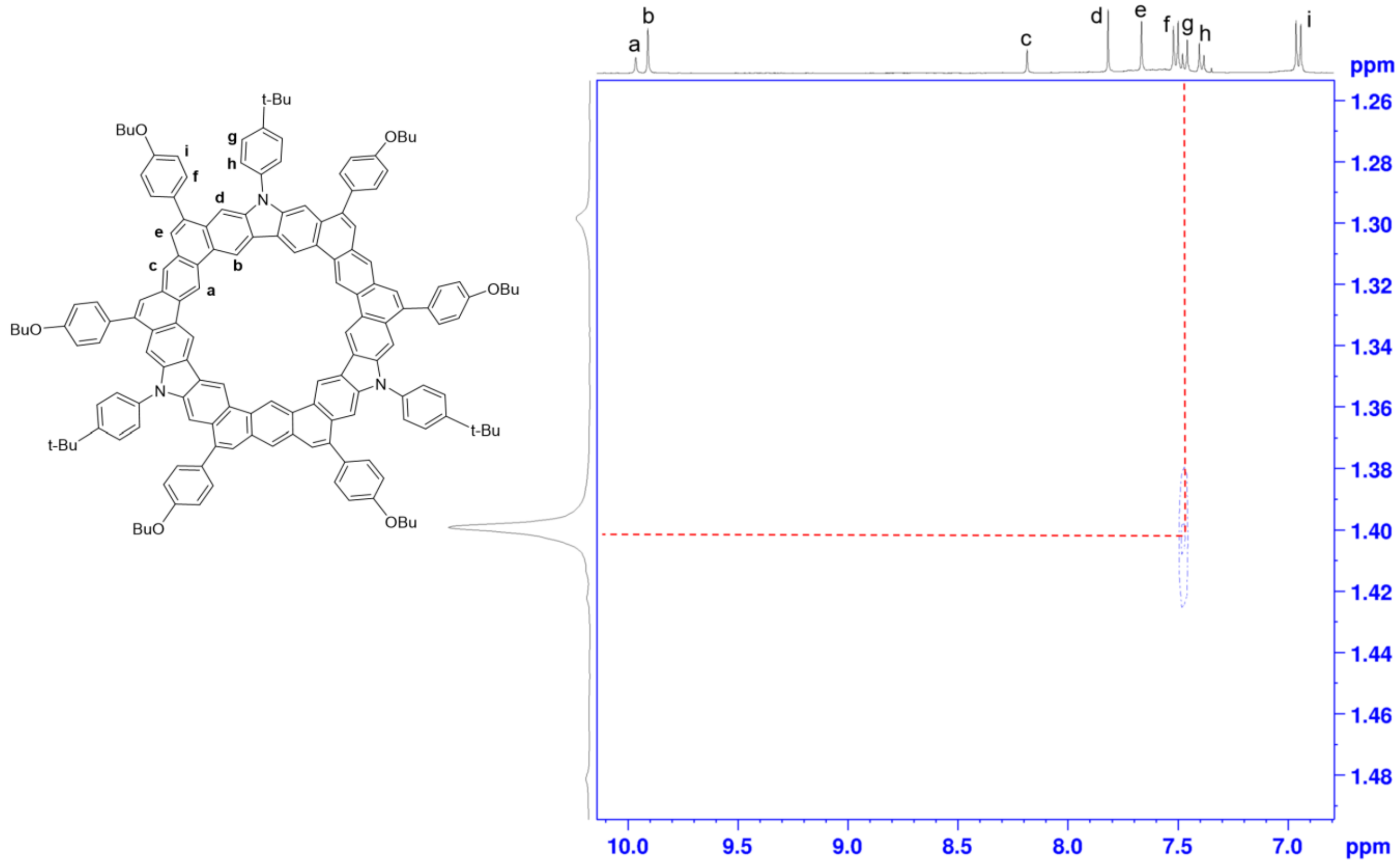

Figure S43. ROESY spectrum of **2b** in CD_2Cl_2 + CS_2 at 298K (aromatic region versus *t*-Bu groups).

3. High-resolution mass spectra

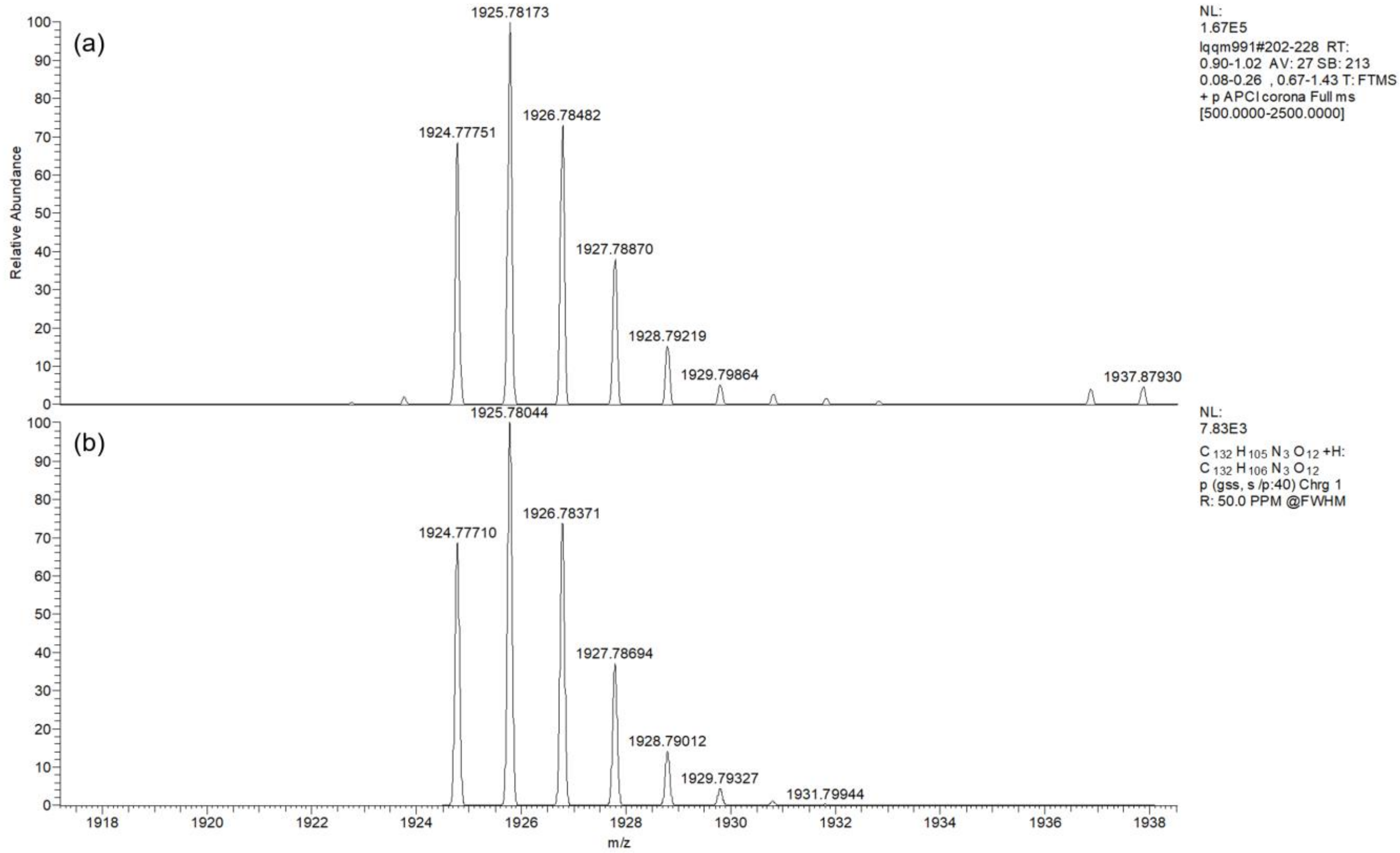

Figure S44. (a) High resolution mass spectrum of **1**. (b) Calculated mass-spectra of **1**.

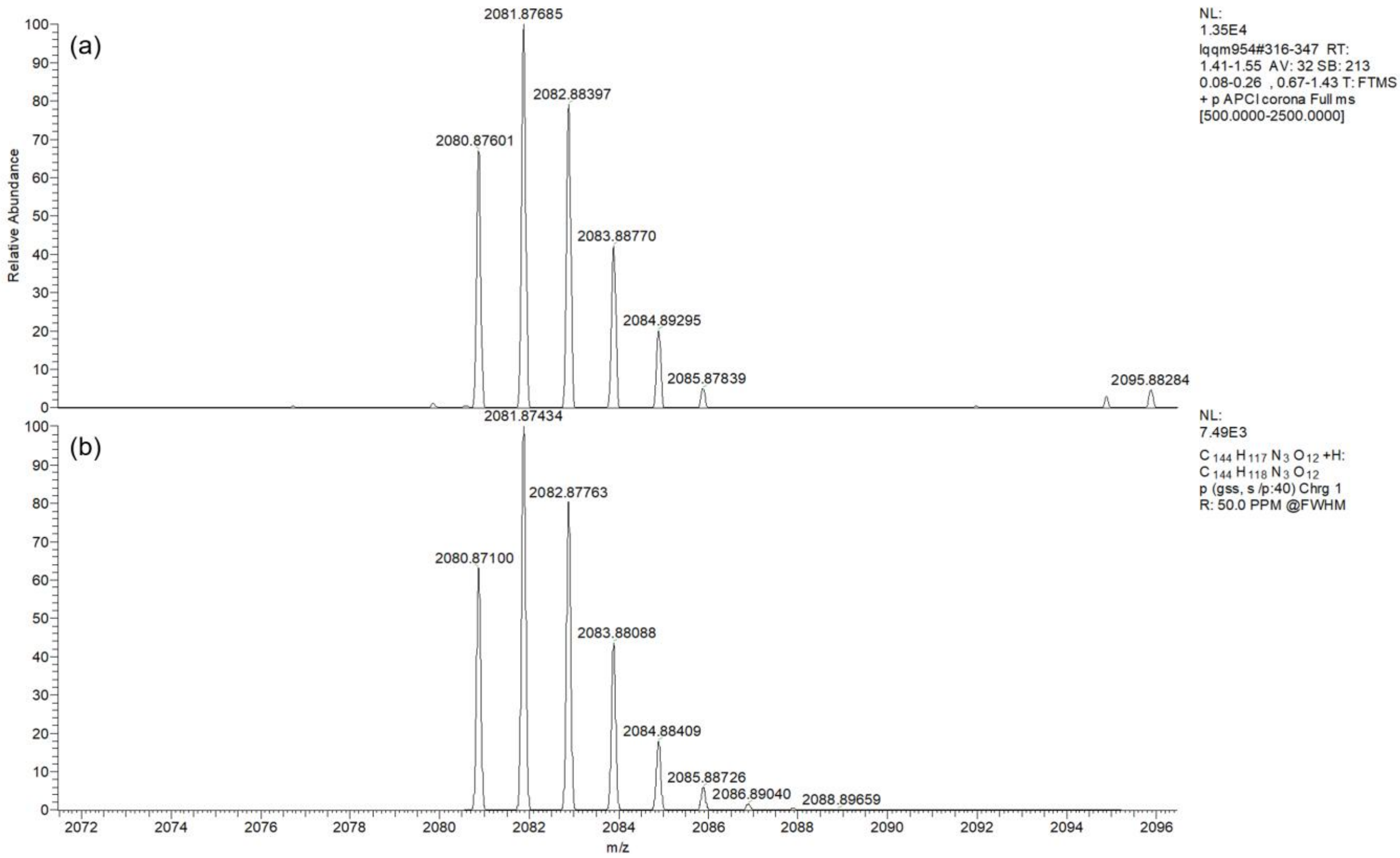

Figure S45. (a) High resolution mass spectrum of **2a**. (b) Calculated mass-spectra of **2a**.

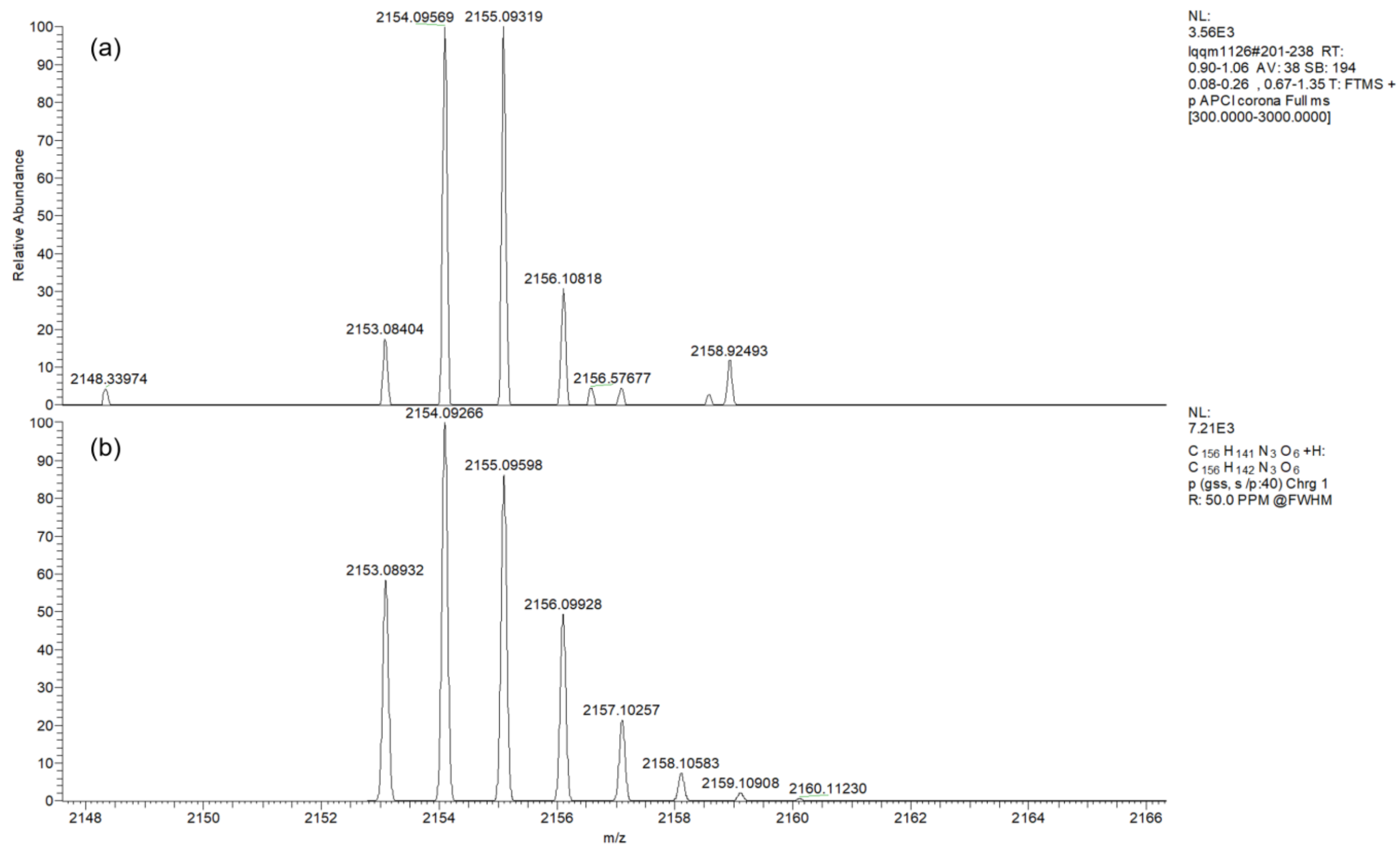

Figure S46. (a) High resolution mass spectrum of **2b**. (b) Calculated mass-spectra of **2b**.

4. Photophysical properties

UV-vis absorption spectra were recorded on a Shimadzu UV-3600 Plus UV-VIS-NIR Spectrophotometer. Fluorescence spectra were taken on a Hitachi F-4500 spectrofluorometer. Circular dichroism (CD) spectra were recorded on a Chirascan ™ Spectrometer (Applied Photophysics). Circularly polarized luminescence (CPL) spectra were recorded on an Applied photophysics Chirascan V100 circular dichroism spectrometer. All optical measurements were performed at room temperature under ambient conditions.

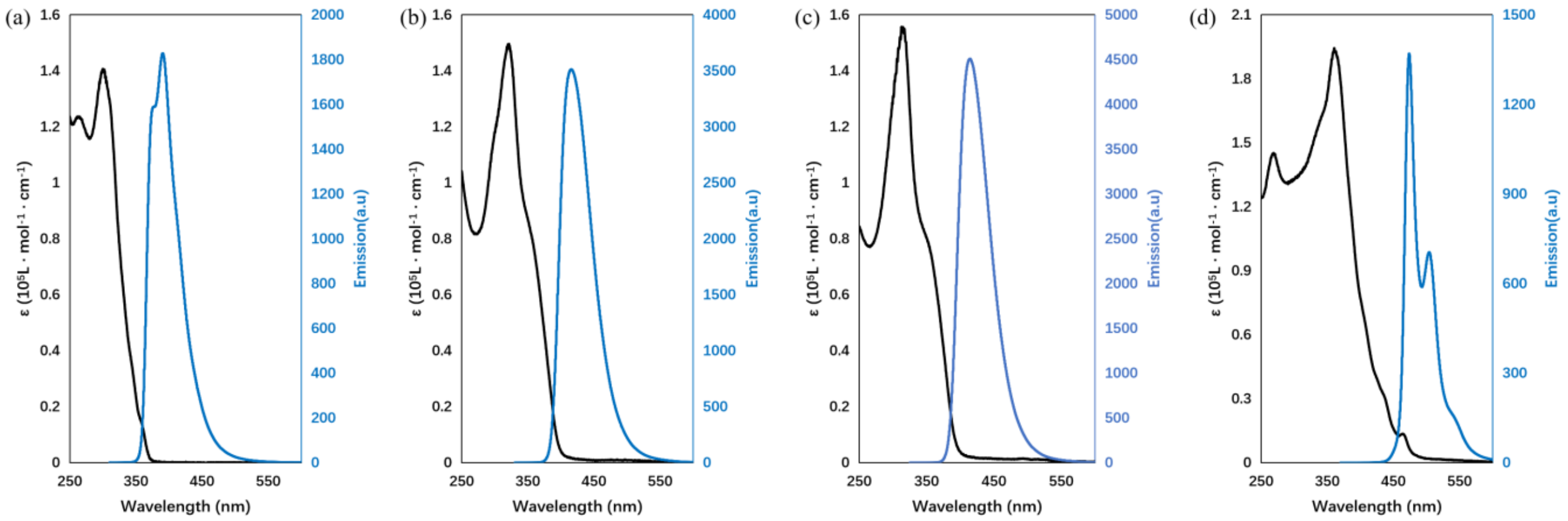

Figure S47. Absorption spectra and photoluminescence spectra of **9** (a), **14a** (b), **14b** (c) and **2a** (d) in CH_2Cl_2 (1.0×10^{-5} mol/L). (The photoluminescence spectra of **9**, **14a**, **14b** and **2a** were measured with excitation at 300, 320, 315 and 360 nm, respectively.)

5. Chiral resolution

Compound **1** was resolved into two enantiomers by Daicel Chiral Technologies (China) Co., Ltd. with a Shimadzu LC-20AT HPLC on a CHIRALPAK® IE column using MeOH/CH_2Cl_2 = 50/50(V/V) as the eluent. From 37.2 mg of racemic Compound **1**, 17.7 mg of one enantiomer (the first fraction) and 17.6 mg of the other enantiomer (the second fraction) were obtained.

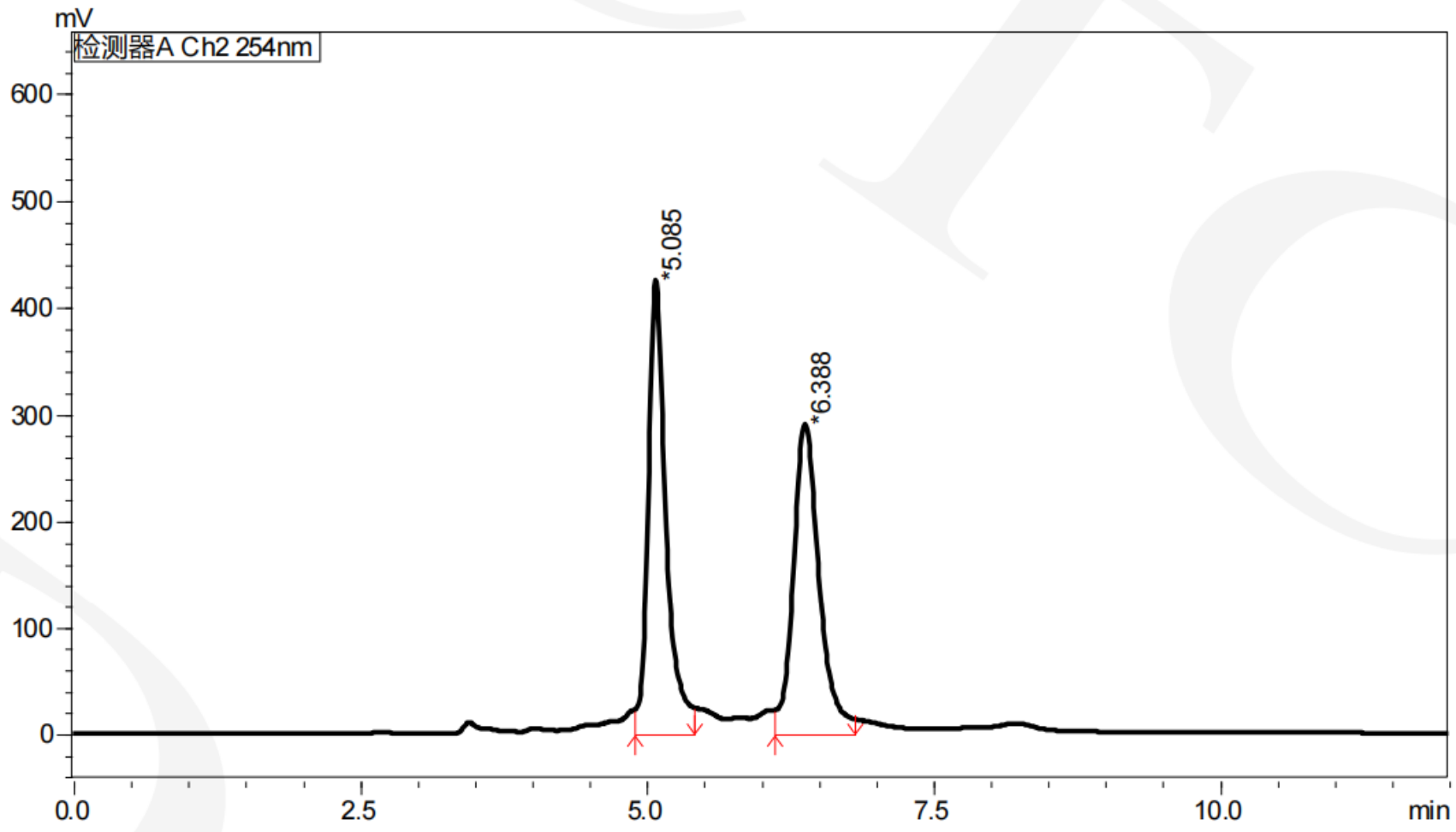

<Peak Table>

Peak#	Ret. Time	Area	Area%	T.Plate#	Tailing F.	Resolution
1	5.085	4596290	50.289	5619	--	--
2	6.388	4543443	49.711	4148	--	3.899

Figure S48. Chiral HPLC trace for resolution of Compound **1**.

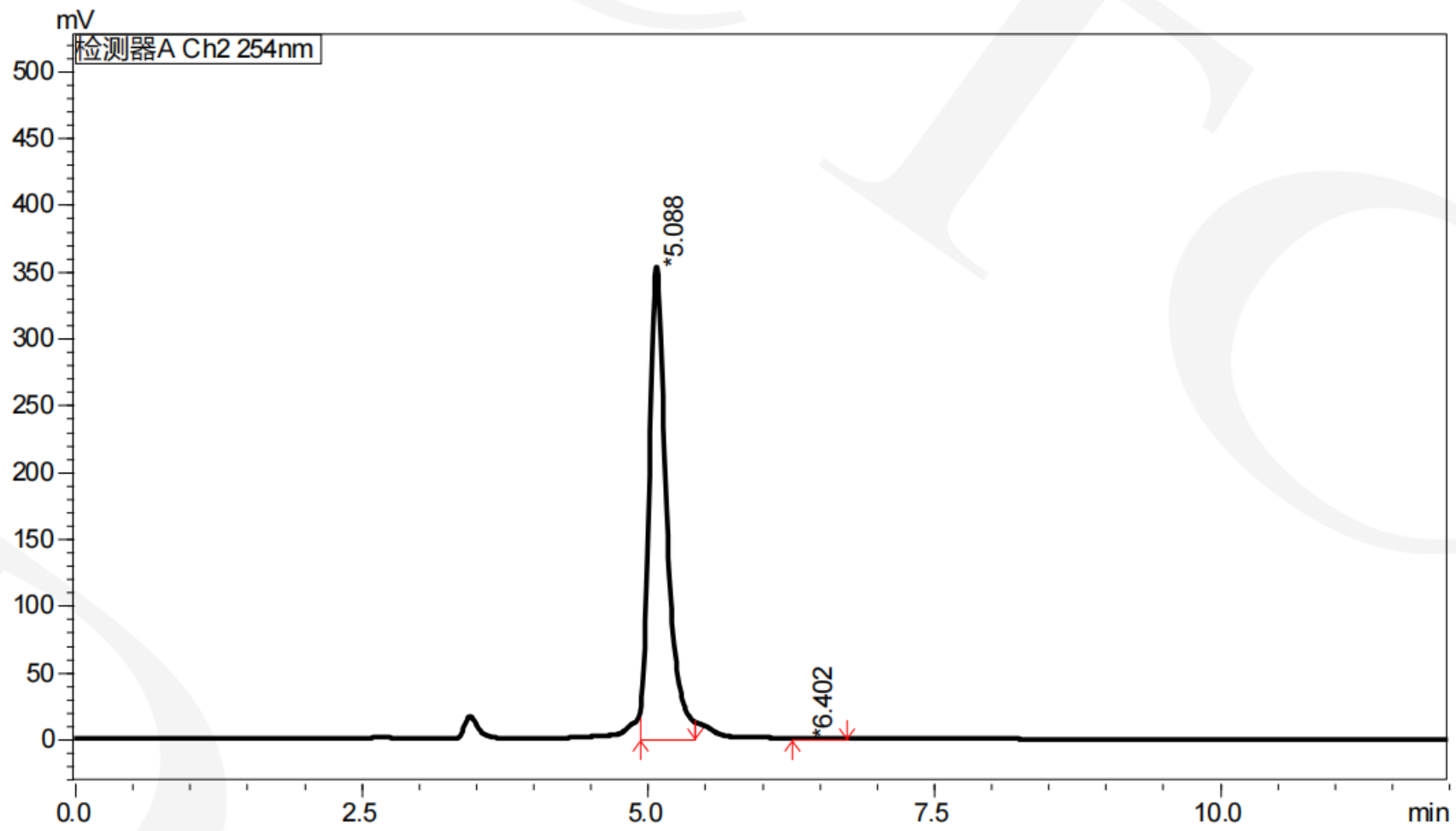

<Peak Table>

Peak#	Ret. Time	Area	Area%	T.Plate#	Tailing F.	Resolution
1	5.088	3743212	99.884	5572	1.415	--
2	6.402	4331	0.116	1945	--	3.080

Figure S49. Chiral HPLC analysis for the first fraction of Compound **1**. $[\alpha]^{25}D$ = +1489.47 (c = 2×10^{-4} g/ml in CH_2Cl_2).

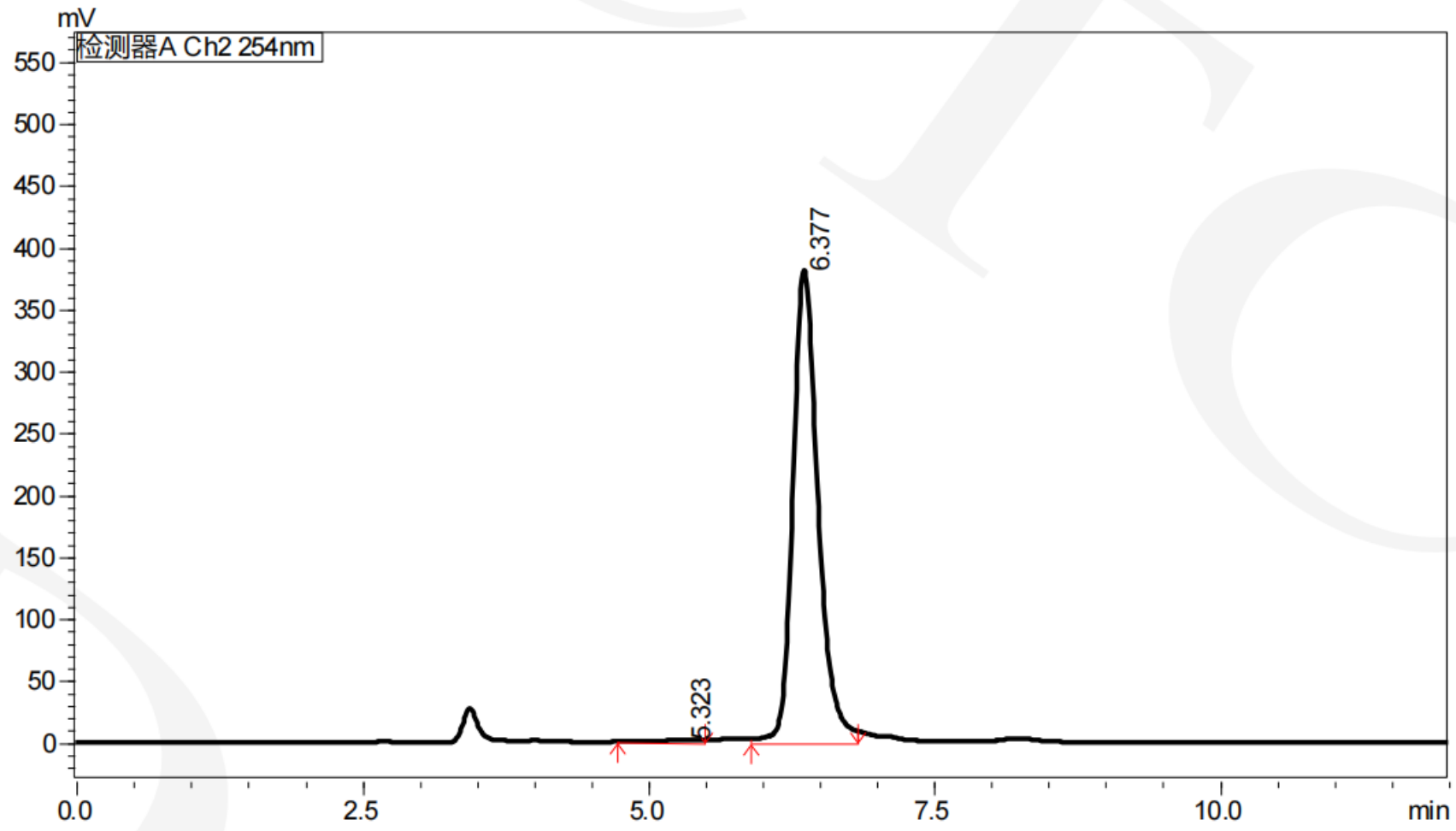

<Peak Table>

Peak#	Ret. Time	Area	Area%	T.Plate#	Tailing F.	Resolution
1	5.323	42032	0.718	587	--	--
2	6.377	5810059	99.282	4204	1.273	1.656

Figure S50. Chiral HPLC analysis for the second fraction of Compound **1**. $[\alpha]^{25}D$ = -1478.95 (c = $2x10^{-4}$ g/ml in CH_2Cl_2).

6. X-ray crystallography data

X-ray diffraction data collection was performed on a Bruker D8 VENTURE diffractometer equipped with a Cu-target iμs 3.0 microfocus X-ray source (λ = 1.54178 Å) and a PHOTON III C14 detector, under a temperature of 150(2) K (Oxford Cryosystem 800). The dataset's reduction and integration were performed with the Bruker software package SAINT (version 8.40B).[7] Data were corrected for absorption effects using the empirical methods as implemented in SADABS (version 2016/2).[8] The structures were solved by SHELXT (version 2018/3) [9] and refined by full-matrix least-squares procedures using the SHELXL (version 2022/12) [10] software package. All non-hydrogen atoms were refined anisotropically. The H-atoms were included at calculated positions and refined as riders, with $U_{iso}(H) = 1.2\ U_{eq}(C)$. In each unit cell of **1**, 92 hexane solvent molecules were found to be severely disordered and removed by the Olex2's solvent mask subroutine.[11] The total void volume was 21903 Å^3, equivalent to 39.3 % of the unit cell's total volume. Crystallographic data and details of the data collection and structure refinement are listed in the Table below.

Crystal data and structure refinement for **1**.

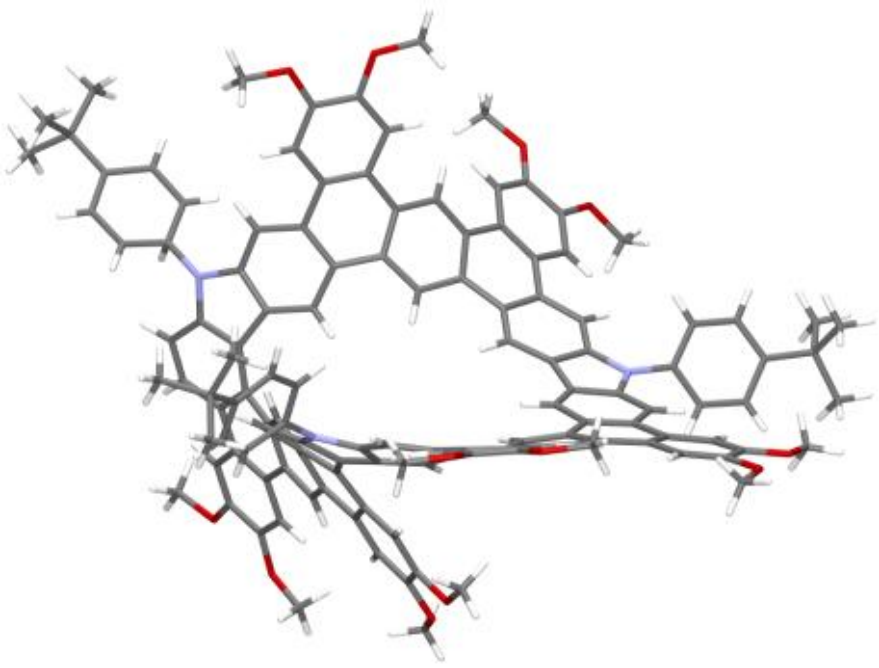

Compound	**1**
Empirical formula	$C_{333}H_{372}N_6O_{24}$
Formula weight	4842.34
Temperature (K)	150(2)
Wavelength (Å)	1.54178
Crystal system	Monoclinic
Space group	*C*2/*c*
a (Å)	22.7874(16)
b (Å)	39.365(3)
c (Å)	63.001(4)
α (°)	90
β (°)	99.362(4)
γ (°)	90
V (Å^3)	55761(7)
Z	8
ρ_{calcd} (g·cm^{-3})	1.154

μ (mm^{-1})	0.553
F(000)	20832
Crystal size (mm)	0.15×0.2×0.2
θ range for data collection (°)	2.355-61.268
Index ranges	-25<=h<=19, -43<=k<=43, -71<=l<=53
Reflections collected	183603
Independent reflections	41402 [R_{int} = 0.1124]
Completeness	96.4%
Transmission factors (min/max)	0.6242/0.7516
Data/restraints/parameters	41402/693/2853
$R1$,[a] $wR2$[b] ($I > 2\sigma(I)$)	0.1057, 0.3104
$R1$,[a] $wR2$[b] (all data)	0.1858, 0.3742
Quality-of-fit[c]	1.031
Largest diff. peak and hole	0.4 and -0.4 e.Å^{-3}

$R_{int} = \Sigma|F_o^2 - \langle F_o^2 \rangle| / \Sigma|F_o^2|$

[a]$R_1 = \Sigma||F_o|-|F_c|| / \Sigma|F_o|$. [b]$wR_2 = [\Sigma[w(F_o^2-F_c^2)^2] / \Sigma[w(F_o^2)^2]]$.

[c]Quality-of-fit = $[\Sigma[w(F_o^2-F_c^2)^2]/(N_{obs}-N_{params})]^{½}$, based on all data.

Crystal data and structure refinement for **5**.

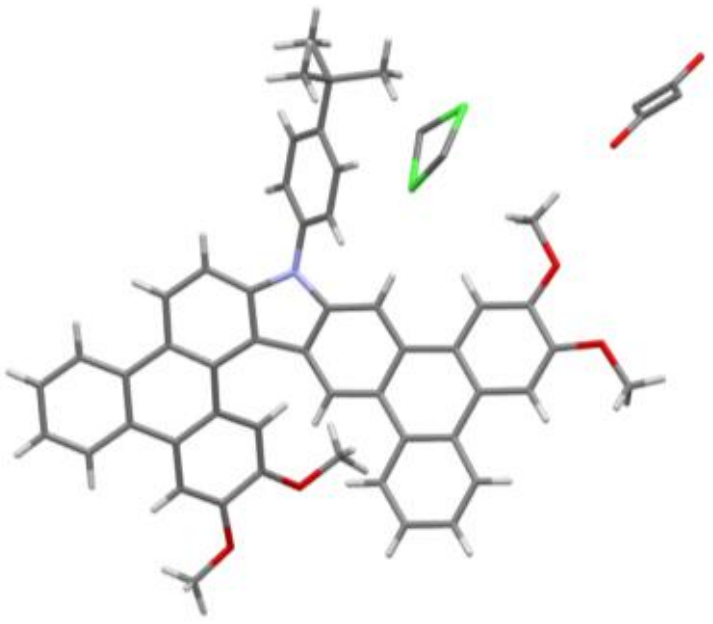

Compound	**5**
Empirical formula	$C_{50}H_{40}NO_4$, CH_2Cl_2, $0.5C_3H_8O$
Formula weight	833.80
Temperature (K)	164(2)
Wavelength (Å)	0.71073
Crystal system	Triclinic
Space group	P-1
a (Å)	10.920(5)
b (Å)	12.348(6)
c (Å)	17.549(7)
α (°)	71.232(12)
β (°)	86.254(13)
γ (°)	67.83(3)
V (Å^3)	2070.5(15)
Z	2
ρ_{calcd} (g·cm^{-3})	1.337

μ (mm^{-1})	0.208
F(000)	876
Crystal size (mm)	0.3×0.4×0.5
θ range for data collection (°)	2.256-28.004
Index ranges	-14<=h<=14, -16<=k<=16, -23<=l<=23
Reflections collected	52592
Independent reflections	9814 [R_{int} = 0.1267]
Completeness	98.9 %
Transmission factors (min/max)	0.6657/0.7456
Data/restraints/parameters	9814/28/560
$R1$,[a] $wR2$[b] ($I > 2\sigma(I)$)	0.1213, 0.2622
$R1$,[a] $wR2$[b] (all data)	0.1892, 0.2987
Quality-of-fit[c]	1.062
Extinction coefficient	0.0123(18)
Largest diff. peak and hole	1.338 and -1.403 e.$Å^{-3}$

$R_{int} = \Sigma|F_o^2 - \langle F_o^2 \rangle| / \Sigma|F_o^2|$

[a]$R_1 = \Sigma||F_o|-|F_c|| / \Sigma|F_o|$. [b]$wR_2 = [\Sigma[w(F_o^2-F_c^2)^2] / \Sigma[w(F_o^2)^2]]$.

[c]Quality-of-fit = $[\Sigma[w(F_o^2-F_c^2)^2]/(N_{obs}-N_{params})]^{½}$, based on all data.

7. Computational studies

All the DFT calculations were done by using Gaussian 16 program.[12] Gibbs free energy of **1** and its constitutional isomers **13a** and **13b** were calculated at the B3LYP /6-31g(d) level of DFT. NICS values of **1** was calculated using the standard GIAO method at the level of B3LYP/6-31G(d,p).[13] ACID plot (B3LYP/6- 31G(d)) was calculated by using the method developed by Herges based on the optimized ground-state geometries and only π-electrons are considered.[14]

Molecule	G (hartree)	Relative G (kcal/mol)
**1**	−3864737.436	0
**13a**	−3864734.911	2.823
**13b**	−3864727.711	9.914

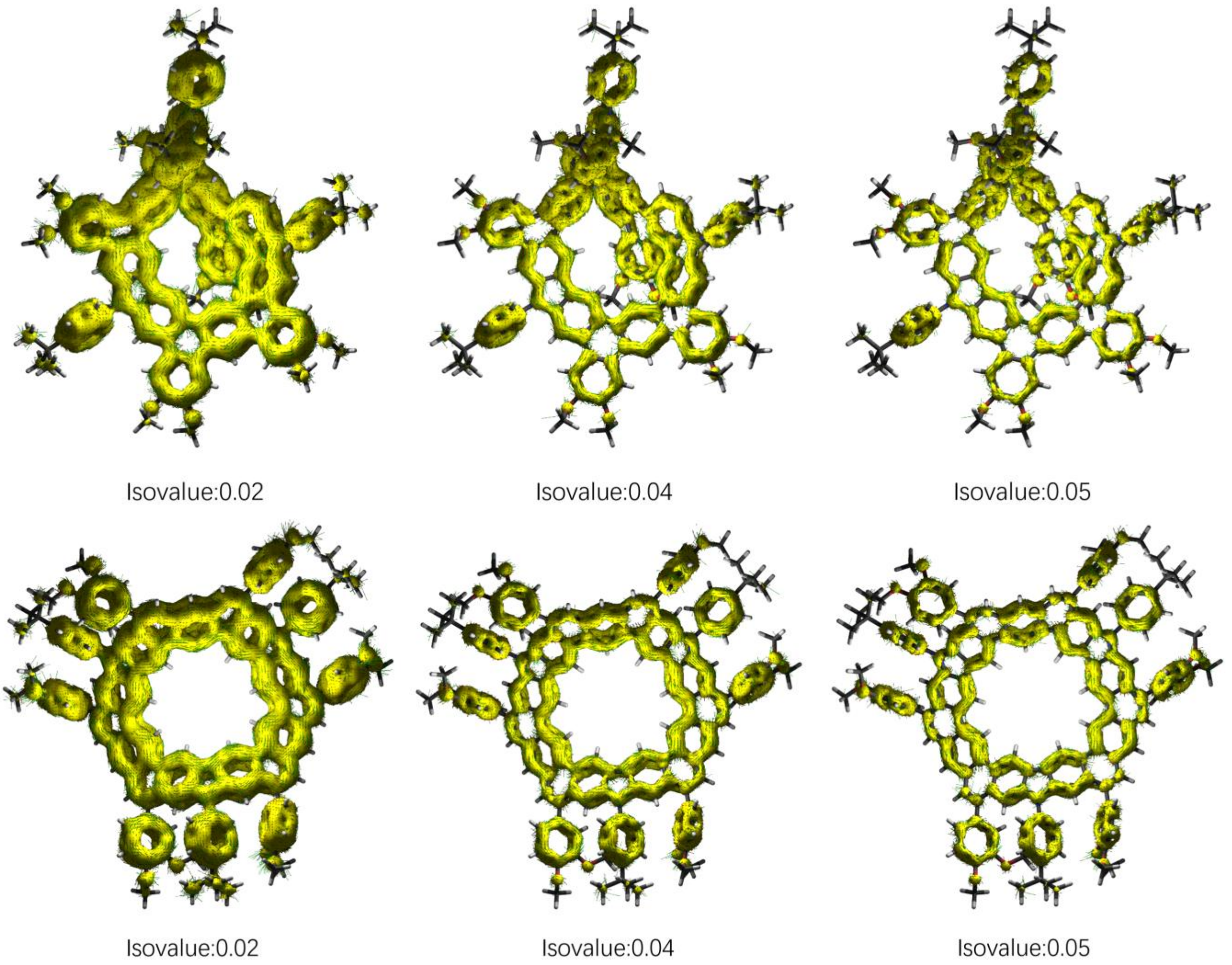

Figure S51. Calculated ACID maps of **1** (top) and **2a** (bottom) with different isovalues based on π-electron contributions under an external magnetic field oriented perpendicular to the plane and pointing outward.

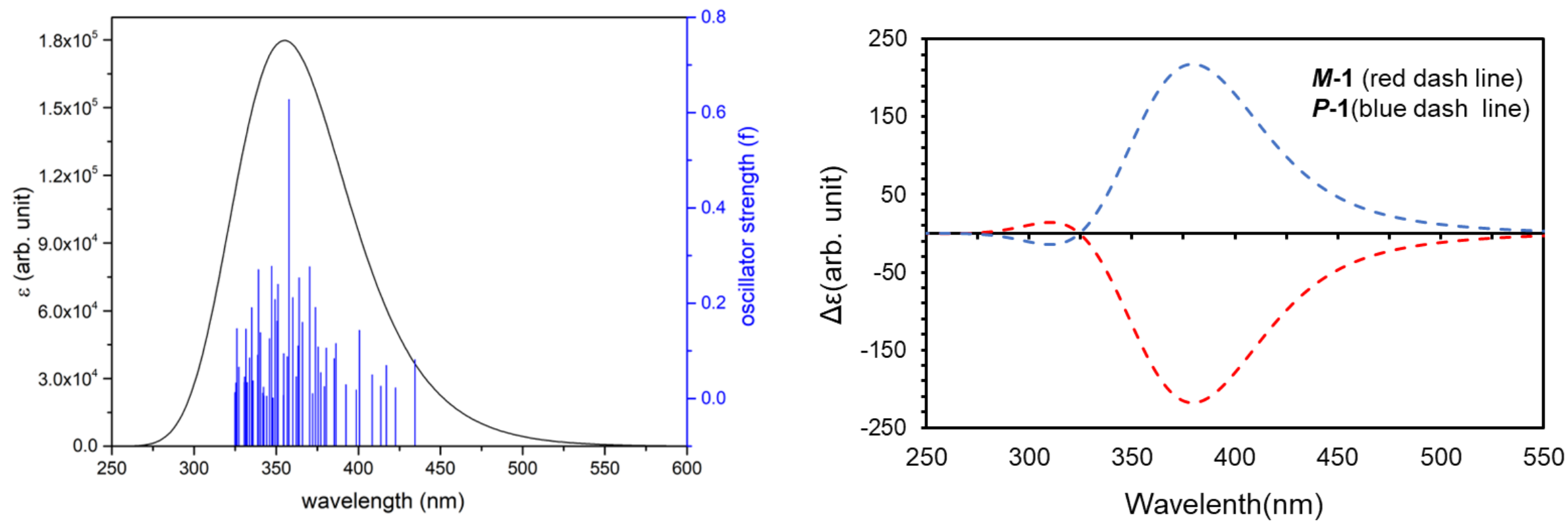

Figure S52. Simulated UV absorption spectra (left) and CD spectra (right) of **1**.

8. NMR titration experiments

Anion binding experiments, monitored using ^{1}H NMR spectroscopy, showed upfield relocations of the inner CH signal when the solution of **2b** in CD_2Cl_2/CS_2 was titrated with tetrabutylammonium tetrafluoroborate. The titration conditions for compound **2b** is summarized in the table below.

[Host] (M)	[Guest] (M)	[Guest]/[Host]	Chemical shift δ (ppm)	Δδ (ppm)
0.002	0	0	9.9340	0
0.002	0.002	1	9.9405	0.0065
0.002	0.004	2	9.9524	0.0184
0.002	0.006	3	9.9664	0.0324
0.002	0.012	6	10.0013	0.0673
0.002	0.02	10	10.0404	0.1064
0.002	0.024	12	10.0663	0.1323
0.002	0.028	14	10.0807	0.1467
0.002	0.032	16	10.1037	0.1697
0.002	0.036	18	10.1223	0.1883
0.002	0.04	20	10.1324	0.1984
0.002	0.044	22	10.1434	0.2094
0.002	0.05	25	10.1519	0.2179
0.002	0.056	28	10.1649	0.2309

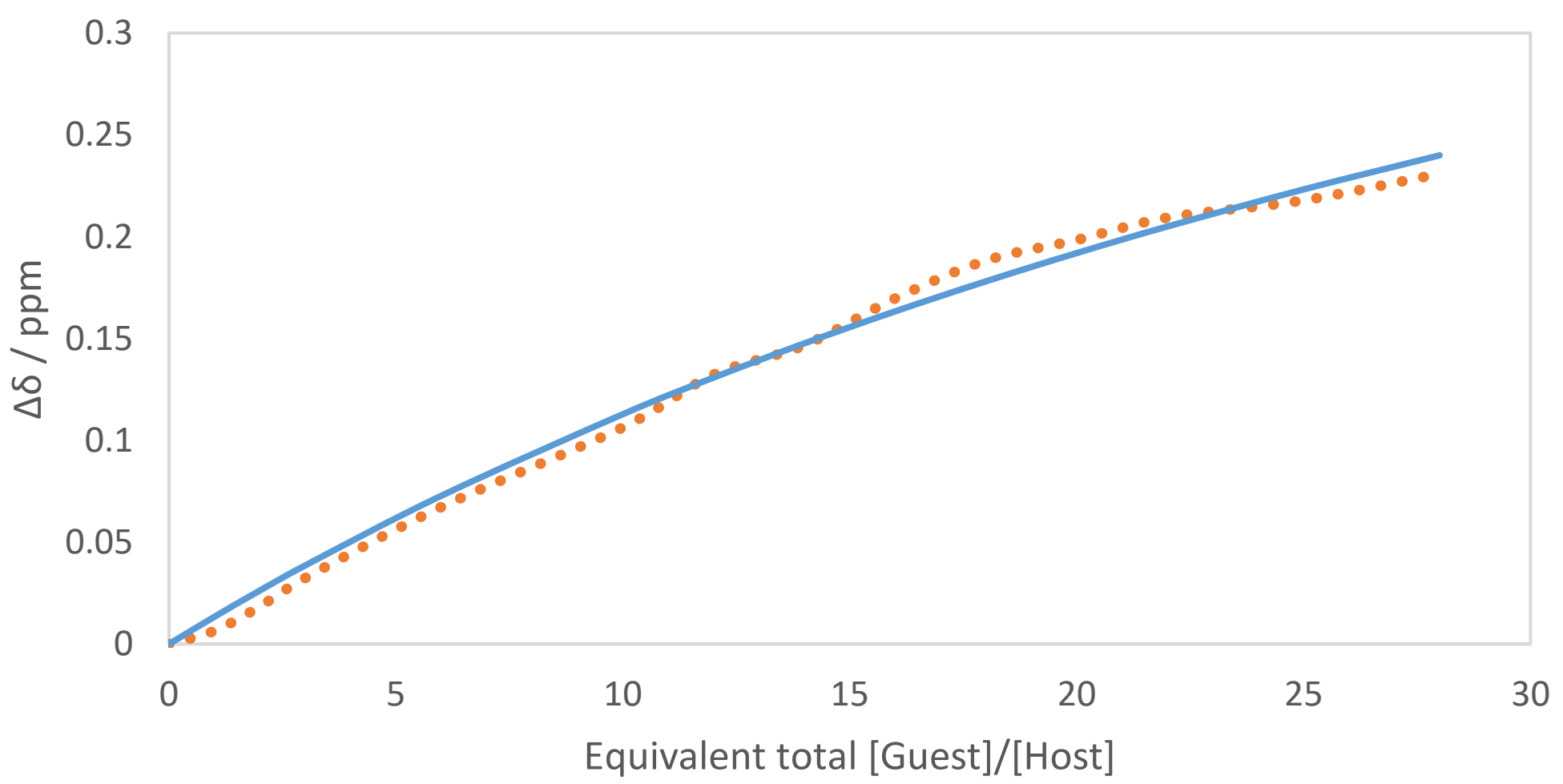

Figure S53. Determination of the binding constant of **2b⊃BF₄⁻**. Changes in the ^{1}H NMR chemical shift of **2b** (proton *b*) during titration with tetrabutylammonium tetrafluoroborate are shown with orange dots, which are fitted with a theoretical curve (blue line) for 1:1 binding using BindFit.[15]

9. References

[1] L.-J. Wei, J. Li, K. Xue, S.-H. Ye, H.-J. Jiang, Synthesis and structure-property correlation of blue fluorescence isomer emitters based on rigid pyrazine-bridged carbazole frameworks *New J. Chem.* **43** (2019): 16629.

[2] S. Maddala, A. Panua, P. Venkatakrishnan, Steering Scholl Oxidative Heterocoupling by Tuning Topology and Electronics for Building Thiananographenes and Their Functional N－/C－Congeners *Chem. Eur. J.* **27** (2021): 16013.

[3] S. Arndt, M.-M. Hansmann, F. Rominger, M. Rudolph, A. S. K. Hashmi, Direct Access to π-Extended Phosphindolium Salts by SimpleProton-Induced Cyclization of (o-Alkynylphenyl)phosphanes. *Chem. Eur. J.* **23** (2017): 5429.

[4] K. Thakur, D. Wang, S.-V. Lindeman, R. Rathore, Synthesis of Doubly Annulated m-Terphenyl-Based Molecular Tweezers and Their Charge-Transfer Complexes with DDQ as a Guest. *Chem. Eur. J.* **24** (2018): 13106.

[5] S.-P. Khandare, A. Mandal, A. Patra, R.-W. Bates, Donor acceptor phenanthrenes and benzoquinolines: synthesis by C-H activation and their photophysical properties. *Org. Biomol. Chem.* **23** (2025): 9424.

[6] J.-R. Flores, G.-C. León, G. Turlakov, E. Arias, I. Moggio, S.-M. Montemayor, R. Torres, R. Ledezma, R.-F. Ziolo, J. González-Torres, Dual Emission of meso-Phenyleneethynylene-BODIPY Oligomers: Synthesis, Photophysics, and Theoretical Optoelectronic Study. *Chem. Eur. J.* **27** (2021): 2493.

[7] SAINT; part of Bruker APEX5 software package (version 2023.9-4): Bruker AXS. 2023.

[8] SADABS; part of Bruker APEX5 software package (version 2023.9-4): Bruker AXS. 2023.

[9] G. M. Sheldrick, SHELXT - Integrated Space-Group and Crystal-Structure Determination. *Acta Crystallogr.* **A71** (2015): 3.

[10] G. M. Sheldrick, Crystal Structure Refinement with SHELXL. *Acta Crystallogr.* **C71**, (2015): 3.

[11] O. V. Dolomanov, L. J. Bourhis, R. J. Gildea, J. A. K. Howard, H. Puschmann, OLEX2: A Complete Structure Solution, Refinement and Analysis Program. *J. Appl. Crystallogr.* **42** (2009): 339.

[12] M. J. Frisch, G. W. Trucks, H. B. Schlegel, G. E. Scuseria, M. A. Robb, J. R. Cheeseman, G. Scalmani, V. Barone, G. A. Petersson, H. Nakatsuji, X. Li, M. Caricato, A. Marenich, J. Bloino, B. G. Janesko, R. Gomperts, B. Mennucci, H. P. Hratchian, J. V. Ortiz, A. F. Izmaylov, J. L. Sonnenberg, D. Williams-Young, F. Ding, F. Lipparini, F. Egidi, J. Goings, B. Peng, A. Petrone, T. Henderson, D. Ranasinghe, V. G. Zakrzewski, J. Gao, N. Rega, G. Zheng, W. Liang, M. Hada,

M. Ehara, K. Toyota, R. Fukuda, J. Hasegawa, M. Ishida, T. Nakajima, Y. Honda, O. Kitao, H. Nakai, T. Vreven, K. Throssell, J. A. Montgomery, Jr., J. E. Peralta, F. Ogliaro, M. Bearpark, J. J. Heyd, E. Brothers, K. N. Kudin, V. N. Staroverov, T. Keith, R. Kobayashi, J. Normand, K. Raghavachari, A. Rendell, J. C. Burant, S. S. Iyengar, J. Tomasi, M. Cossi, J. M. Millam, M. Klene, C. Adamo, R. Cammi, J. W. Ochterski, R. L. Martin, K. Morokuma, O. Farkas, J. B. Foresman, D. J. Fox, *Gaussian 16 Rev. B.01,* Wallingford, CT, 2016.

[13] Z. Chen, C. S. Wannere, C. Corminboeuf, R. Puchta, Nucleus-Independent Chemical Shifts (NICS) as an Aromaticity Criterion. *Chem. Rev.* **105** (2005): 3842.

[14] D. Geuenich, K. Hess, F. Köhler, R. Herges, Anisotropy of the Induced Current Density (ACID), a General Method to Quantify and Visualize Electronic Delocalization. *Chem. Rev.* **105** (2005): 3758.

[15] https://app.supramolecular.org
